# Asymmetric Synthesis of 2,4,5-Trisubstituted Δ^2^-Thiazolines

**DOI:** 10.1002/chem.201301120

**Published:** 2013-06-17

**Authors:** Christoffer Bengtsson, Hanna Nelander, Fredrik Almqvist

**Affiliations:** [a] Department of Chemistry, Umeå University90187 Umeå (Sweden); [b] AstraZeneca R&D MölndalMedicinal Chemistry, 43183 Mölndal (Sweden); [c] Laboratories for Chemical Biology UmeåLCBU, Umeå University, 90187 Umeå (Sweden); [d] Umeå Center for Microbial Research, UCMR, Umeå University90187 Umeå (Sweden)

**Keywords:** acyl migration, asymmetric synthesis, heterocycles, unnatural amino acids, thiazolines

## Abstract

Δ^2^-Thiazolines are interesting heterocycles that display a wide variety of biological characteristics. They are also common in chiral ligands used for asymmetric syntheses and as synthetic intermediates. Herein, we present asymmetric routes to 2,4,5-trisubstituted Δ^2^-thiazolines. These Δ^2^-thiazolines were synthesized from readily accessible/commercially available α,β-unsaturated methyl esters through a Sharpless asymmetric dihydroxylation and an O→N acyl migration reaction as key steps. The final products were obtained in good yields with up to 97% enantiomeric excess.

## Introduction

**Figure 1:**
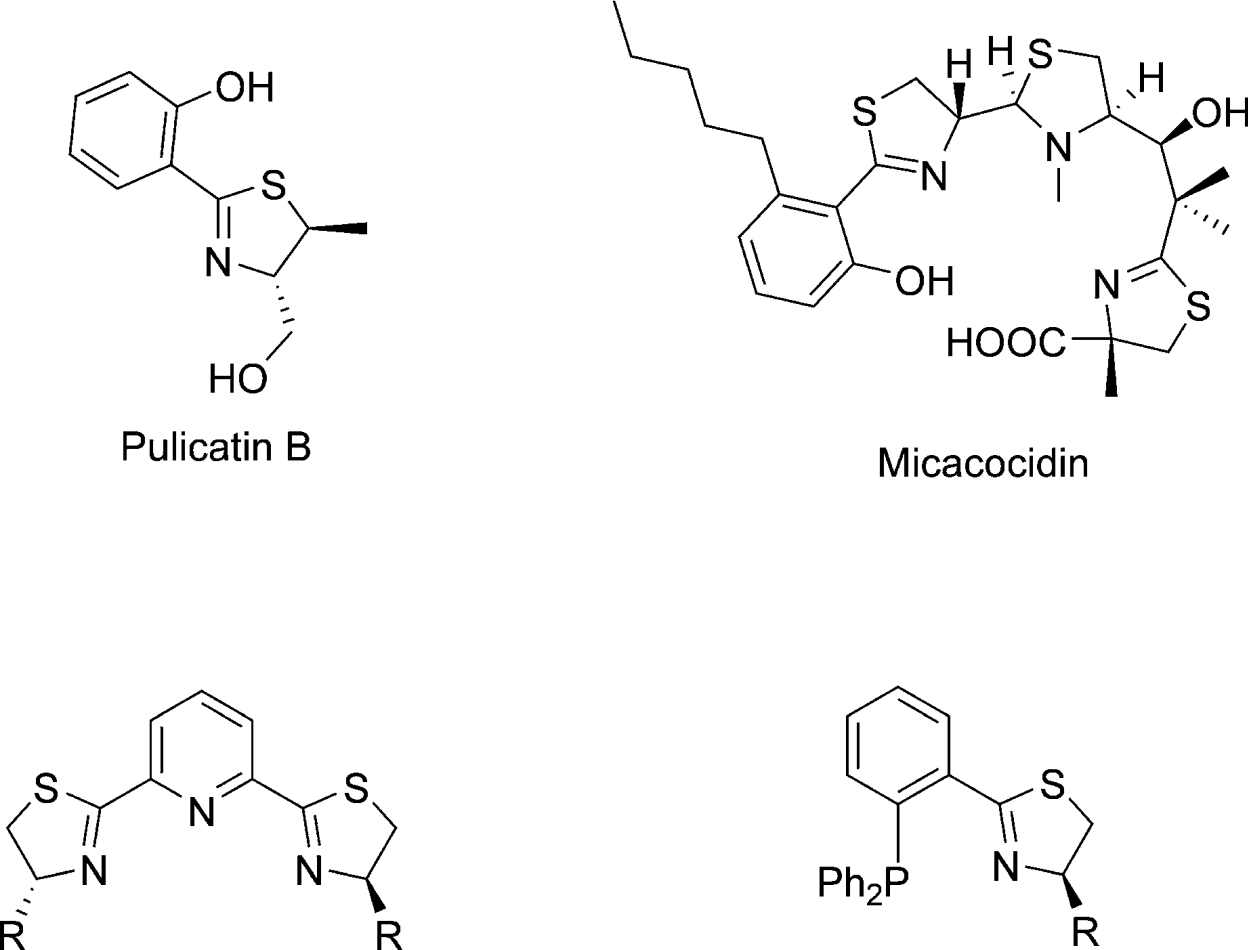
Examples of Δ^2^-thiazoline-containing natural products and chiral ligands.

Δ^2^-Thiazolines constitute a class of compounds with diverse applications. They possess interesting properties as flavoring agents^1^ and pheromones,^2^ are present in the structures of many natural products (e.g., Pulicatin B^3^ and micacocidin^4^), and are useful as chiral ligands in asymmetric synthesis^5^ (Figure 1). As ligands, they have proven successful in a variety of reactions, including Pd-catalyzed allylic substitutions,5a,^6^ Diels–Alder reactions,^7^ Friedel–Crafts alkylations of indoles^8^ and alkylzinc additions to aldehydes.^9^ Many ways to construct the Δ^2^-thiazoline heterocycles have been developed, and they normally involve the use of a dehydrating reagent. Among the known methods are reactions with triethylamine (TEA)/MsCl (Ms=mesityl),5a,^6^ SOCl_2_/pyridine,^10^ the Lawesson reagent,7b,^11^ the Burgess reagent,4c,^10^ the Hendrickson reagent,^12^ TiCl_4_,^13^ PCl_5_,4a P_2_S_5_,^14^ diethylaminosulfur trifluoride (DAST),^15^ [bis(2-methoxyethyl)amino]sulfur trifluoride (Deoxo-Fluor),9a,^16^ PPh_3_/diisopropyl azodicharboxylate (DIAD),^17^ 3-nitrophenylboronic acid,^18^ and Ru/*tert*-butyl hydroperoxide (TBHP).^19^ These processes normally lead to 2,4-disubstituted Δ^2^-thiazolines, with less attention given to the asymmetric synthesis of their 2,4,5-trisubstituted analogues.

**Figure 2:**
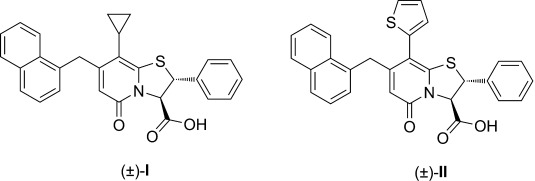
Previously synthesized racemic 2-substituted thiazolino ring-fused 2-pyridones.

In our research group, we are interested in Δ^2^-thiazolines as intermediates in the synthesis of thiazolo/thiazolino ring-fused 2-pyridones,^20^,^21^ which are constructed through a ketene/imine cyclocondensation reaction between a Δ^2^-thiazoline and an acyl Meldrum’s acid derivative.^22^ In the case of 2-substituted thiazolino ring-fused 2-pyridones, only racemates have previously been synthesized (Figure 2), through a conjugate addition reaction with a higher order cuprate onto the corresponding α,β-unsaturated methyl esters.20b The intriguing biological activity demonstrated by these compounds as novel antibacterial agents20c,^23^ encouraged us to develop asymmetric synthetic pathways to the enantiomers of these compounds. The previously developed cuprate addition has proven hard to perform on a larger scale, and also required very fresh organolithium reagents to proceed in satisfactory yields. Consequently, development of an asymmetric version of that method was not an attractive option. Initially, we also considered the Sharpless amino hydroxylation reaction in the synthesis of (±)-**5**. However, that route would give the *syn*-diastereomer of (±)-**5** when performed on the commercially available *trans*-methyl cinnamate **1** and eventually give the *cis*-configured Δ^2^-thiazoline. Additionally, this reaction is known to give mixtures of regioisomers and, in some cases, poor conversions.^24^

**Figure 3:**
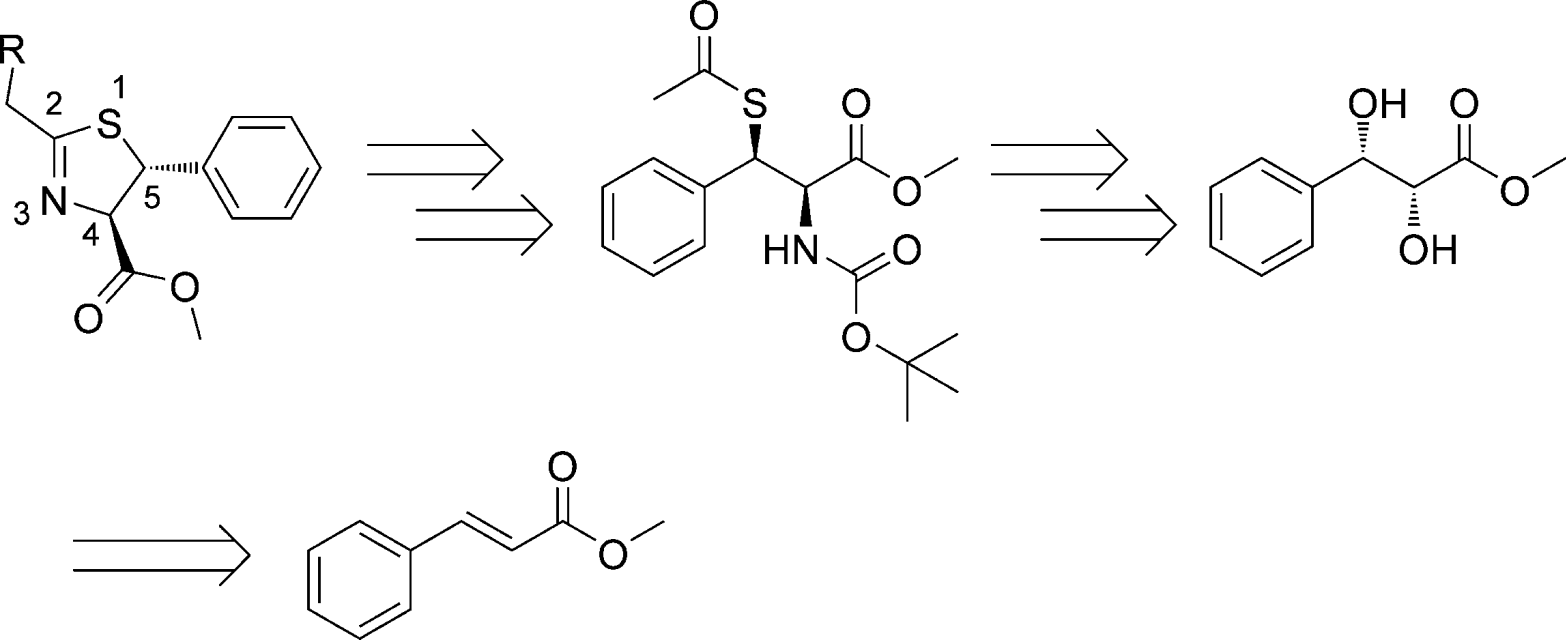
Retrosynthetic analysis of the 5-phenyl-substituted Δ^2^-thiazolines.

We envisioned instead a synthetic pathway starting with a Sharpless asymmetric dihydroxylation of commercially available methyl cinnamate, followed by the selective conversion of the α-hydroxy group into an azide. Subsequently, this azide could be reduced and protected in a two-step one-pot reaction to give the *tert*-butoxycarbonyl (Boc)-protected amino alcohol,15b and the remaining benzylic hydroxy group could then be converted to a thiol through simple functional-group interconversion (FGI). Finally, we envisioned that deprotection followed by a thioamine/imino ether condensation^22^ would give the 5-substituted Δ^2^-thiazoline (Figure 3).

## Results and Discussion

**Scheme 1:**
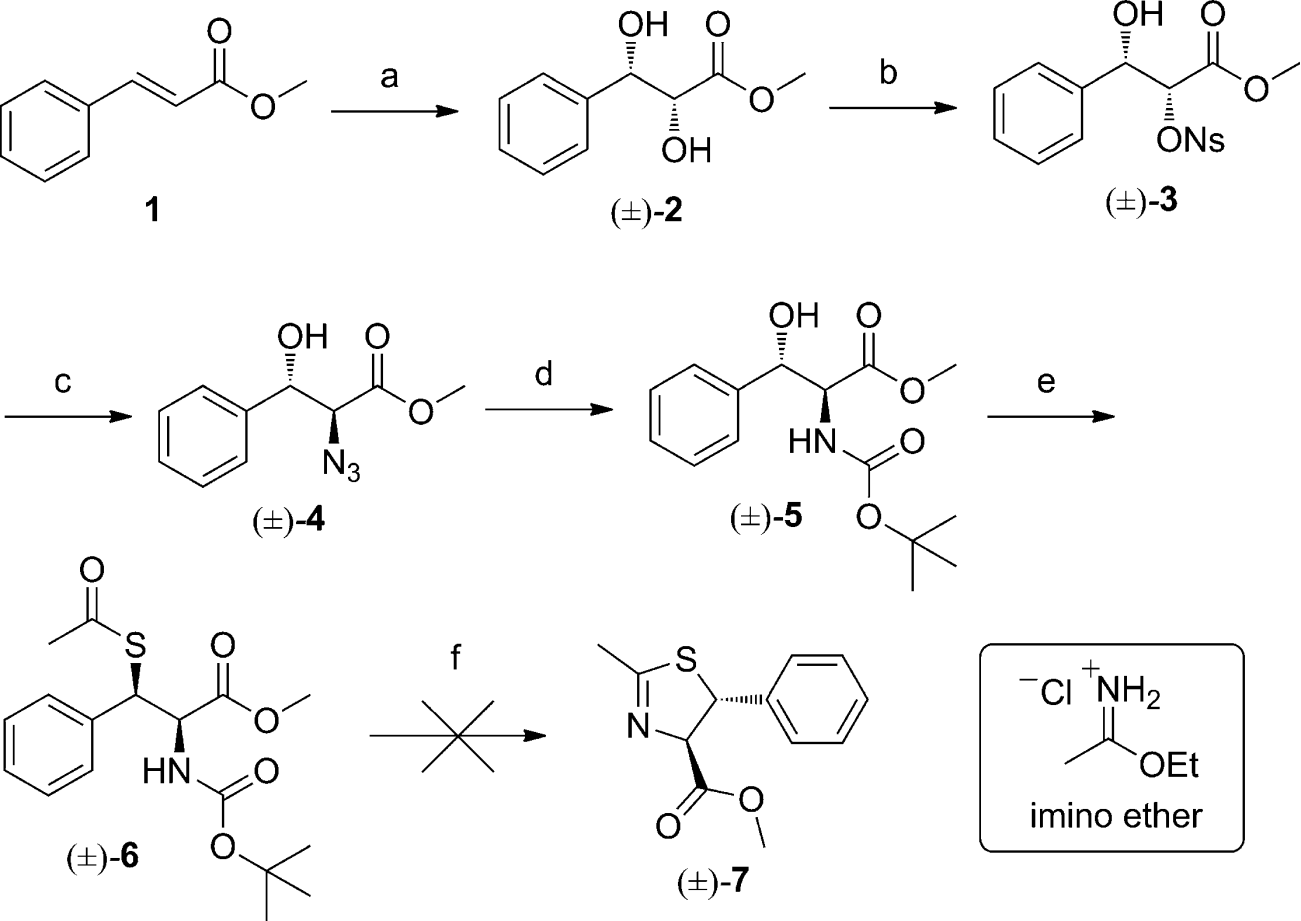
a) K_2_OsO_4_⋅2H_2_O, NMO, MeCN/acetone/H_2_O (1:1:1), RT, 81%; b) NsCl, TEA, CH_2_Cl_2_, 0°C, 75%; c) NaN_3_, DMF, 40°C, 70%; d) i) SnCl_2_⋅2H_2_O, RT; ii) NaHCO_3_, Boc_2_O, 1,4-dioxane/H_2_O, RT, 93%; e) i) Ms_2_O, TEA, CH_2_Cl_2_; ii) KSAc, DMF, RT, 60%; f) i) K_2_CO_3_ or NaOMe, MeOH; ii) TFA or HCl; iii) Imino ether, TEA, CH_2_Cl_2_.

To test our synthetic strategy, we initially performed the dihydroxylation reaction under racemic conditions. Commercially available methyl cinnamate **1** was converted into the corresponding Boc-protected amino alcohol (±)-**5** via diol (±)-**2**, nosylate (±)-**3**, and azide (±)-**4** by following to published procedures for the corresponding ethyl ester (Scheme 1).15b The excellent selectivity for the α-hydroxy group in the nosylation reaction can be attributed to the difference in p*K*_a_ value between the α- and β-hydroxyl groups.^25^ The conversion of the Boc-protected amino alcohol (±)-**5** into the S-acyl/N-Boc-protected amino thiol (±)-**6** went smoothly with mesyl anhydride and potassium thioacetate in 60% yield over two steps. Mesyl anhydride was used instead of MsCl to prevent possible scrambling of the sterogenic center due to displacement of the mesyl sulfonate by the nucleophilic chloride ion. However, all attempts to deprotect and then cyclize this compound with the imino ether to generate the 5-substituted Δ^2^-thiazoline (±)-**7** failed to work and afforded complex mixtures (Scheme 1).

Amido alcohols are known to be suitable precursors for the synthesis of Δ^2^-thiazolines,^11^ and consequently our next method was to prepare the amido alcohol of (±)-**5**. Unfortunately, neither Boc-deprotection followed by *N*,*N*,*N*′,*N*′-tetramethyl-*O*-(benzotriazol-1-yl)uronium tetrafluoroborate (TBTU)/*N*,*N*-diisopropylethylamine (DIPEA)-mediated coupling nor direct coupling of the amine with acid chloride/TEA proved successful.

In our next attempt to synthesize the amido alcohols, we envisioned making the ester of azido alcohol (±)-**4**, which could then undergo a tandem azide reduction/O→N acyl migration reaction. Of the esterification conditions examined for (±)-**4** (TBTU/acid chloride or *N*,*N*-dicyclohexylcarbodiimide (DCC)/4-dimethylaminopyridine (DMAP; Steglich conditions)), DCC/DMAP proved more effective.^26^ To our delight, the SnCl_2_**⋅**2H_2_O-induced azide reduction/O→N acyl migration reaction worked well (Table 1) for all substituted (i.e., R≠H) acyl azido esters (±)-**8** (Table 1, entries 2–5), with the corresponding amido alcohols (±)-**9** isolated in 70–84% yields (Table 1). In addition, this synthetic route remained efficient when performed on a gram scale.

**Table 1:** Synthesis of (*±*)-**9**: a) RCH_2_COOH, DCC, DMAP, CH_2_Cl_2_, RT; b) i) SnCl_2_⋅2H_2_O, RT; ii) NaHCO_3_, MeOH/H_2_O or 1,4-dioxane/H_2_O, RT.
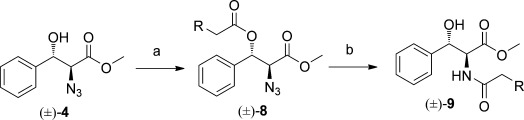

Entry	R	**8**	Yield [%]	**9**	Yield [%]
1	H	(±)-**8a**^a^	96	(±)-**9a**	–
2	*c*-Pr	(±)-**8b**	95	(±)-**9b**^b^	71
3	Ph	(±)-**8c**	95	(±)-**9c**^b^	70
4	*m-*CF_3_Ph	(±)-**8d**	90	(±)-**9d**^b^	80
5	2-thienyl	(±)-**8e**	91	(±)-**9e**^c^	84

[a] Ac_2_O/DMAP was used.

[b] Use of 1,4-dioxane/H_2_O instead of MeOH/H_2_O gave a lower yield

[c] 1,4-Dioxane/H_2_O was used, MeOH/H_2_O gave only a 50% yield.

An initial attempt to prepare the corresponding thiol amide of (±)-**9c** was performed with 0.6 equivalents of the Lawesson reagent (LR) in toluene at reflux (Table 2). Gratifyingly, we observed only thiazoline formation, with (±)-**10b** isolated in 80% yield.

**Table 2:** Synthesis of (*±*)-**10**: a) The Lawesson reagent (0.6 equiv), toluene, reflux.
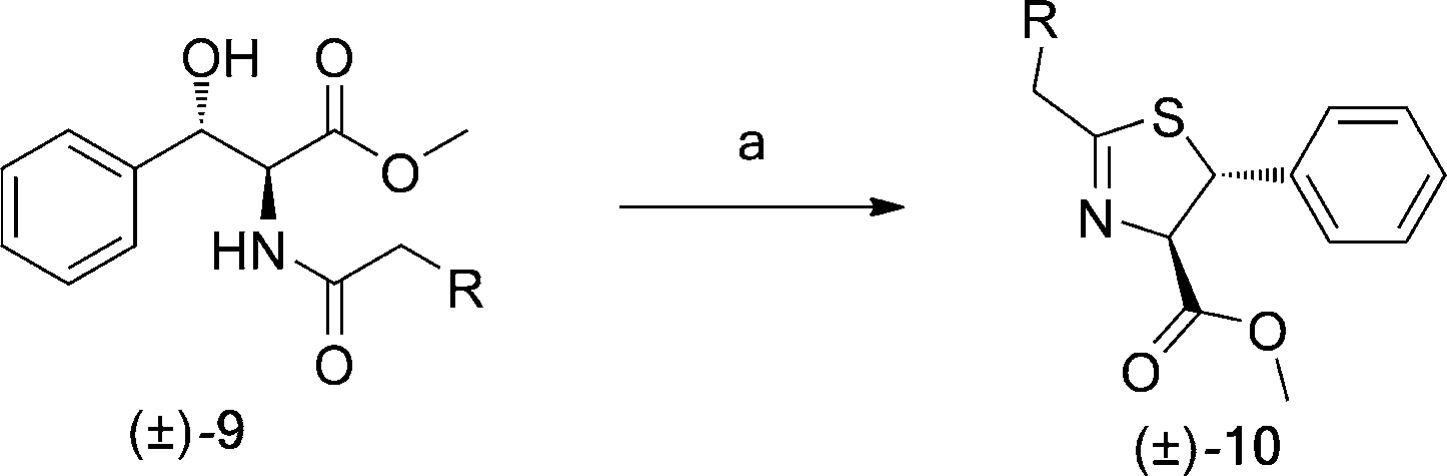

Entry	R	9	**10**	Yield [%]
1	*c*-Pr	(±)-**9b**	(±)-**10a**	71
2	Ph	(±)-**9c**	(±)-**10b**	80
3	*m*-CF_3_Ph	(±)-**9d**	(±)-**10c**	72
4	2-thienyl	(±)-**9e**	(±)-**10d**	74

To investigate the scope and limitations of this reaction, a series of cinnamate analogues (**11**) were synthesized by the Horner–Wadsworth–Emmons olefination reaction in excellent yields and *trans* selectivities (Table 3). The cinnamate analogues (**11**) were subsequently converted into the corresponding azido alcohols (±)-**14** through K_2_OsO_4_**⋅**2H_2_O-catalyzed dihydroxylation and selective α-hydroxy nosylation ((±)-**13**). The nosylates (±)-**13** were then substituted with NaN_3_ to give the corresponding azido alcohols (±)-**14**.

**Table 3:** Synthesis of (*±*)-14: a) NaH, THF, RT; b) K_2_OsO_4_⋅2H_2_O, *N*-methylmorpholine *N*-oxide (NMO), MeCN/acetone/H_2_O (1:1:1), RT; c) NsCl, TEA, CH_2_Cl_2_, 0°C; d) NaN_3_, DMF, 40°C.
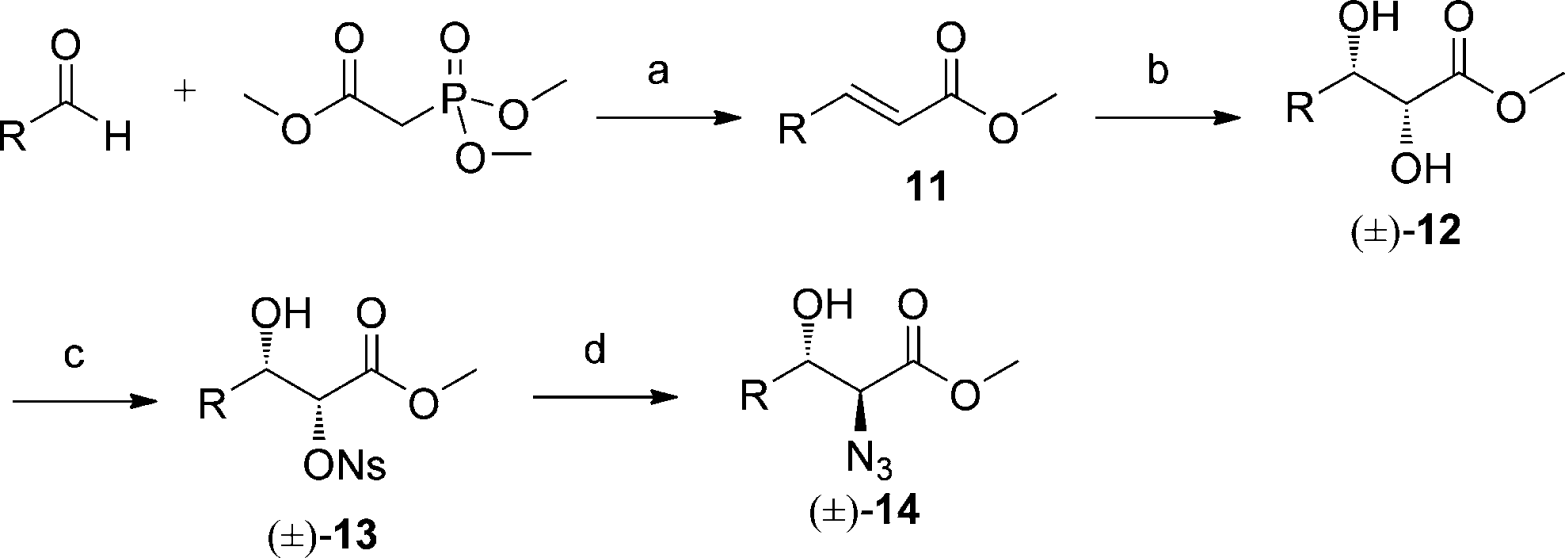

Entry	R	**11**	Yield [%]	**12**	Yield [%]	**13**	Yield [%]	**14**	Yield [%]
1	*p*-FPh	**11a**	98	(±)-**12a**	65	(±)-**13a**	63	(±)-**14a**	65
2	*m*-MeOPh	**11b**	96	(±)-**12b**	75	(±)-**13b**	70	(±)-**14b**	82
3	Bn	**11c**^a^	62	(±)-**12c**	80	(±)-**13c**	62	(±)-**14c**	79
4	2-thienyl	**11d**	98	(±)-**12d**	73	(±)-**13d**	54	(±)-**14d**	78

[a] The aldehyde was freshly distilled prior to use.

The azido alcohols (±)-**14** were then reacted with cyclopropylacetic acid under Steglich esterification conditions to furnish the azido esters (±)-**15** in 75–94% yields (Table 4). The azide reduction/O→N acyl migration reaction was effective for these azido esters, and the corresponding amido alcohols (±)-**16** were isolated in 77–83% yield (Table 4). Treatment of aryl/heteroaryl-substituted amido alcohols (±)-**16a**,**b**,**d** with LR proceeded efficiently to form the desired Δ^2^-thiazolines (±)-**17** (Table 4, entries 1, 2, and 4). However, the reaction to form benzyl substituted (±)-**17c**, which was only isolated in 24% yield (Table 4, entry 3), was less effective.

**Table 4:** Synthesis of (*±*)-**17**: a) Cyclopropylacetic acid, DCC, DMAP, CH_2_Cl_2_, RT; b) i) SnCl_2_⋅2H_2_O, RT; ii) NaHCO_3_, MeOH/H_2_O; c) the Lawesson reagent, toluene, reflux.
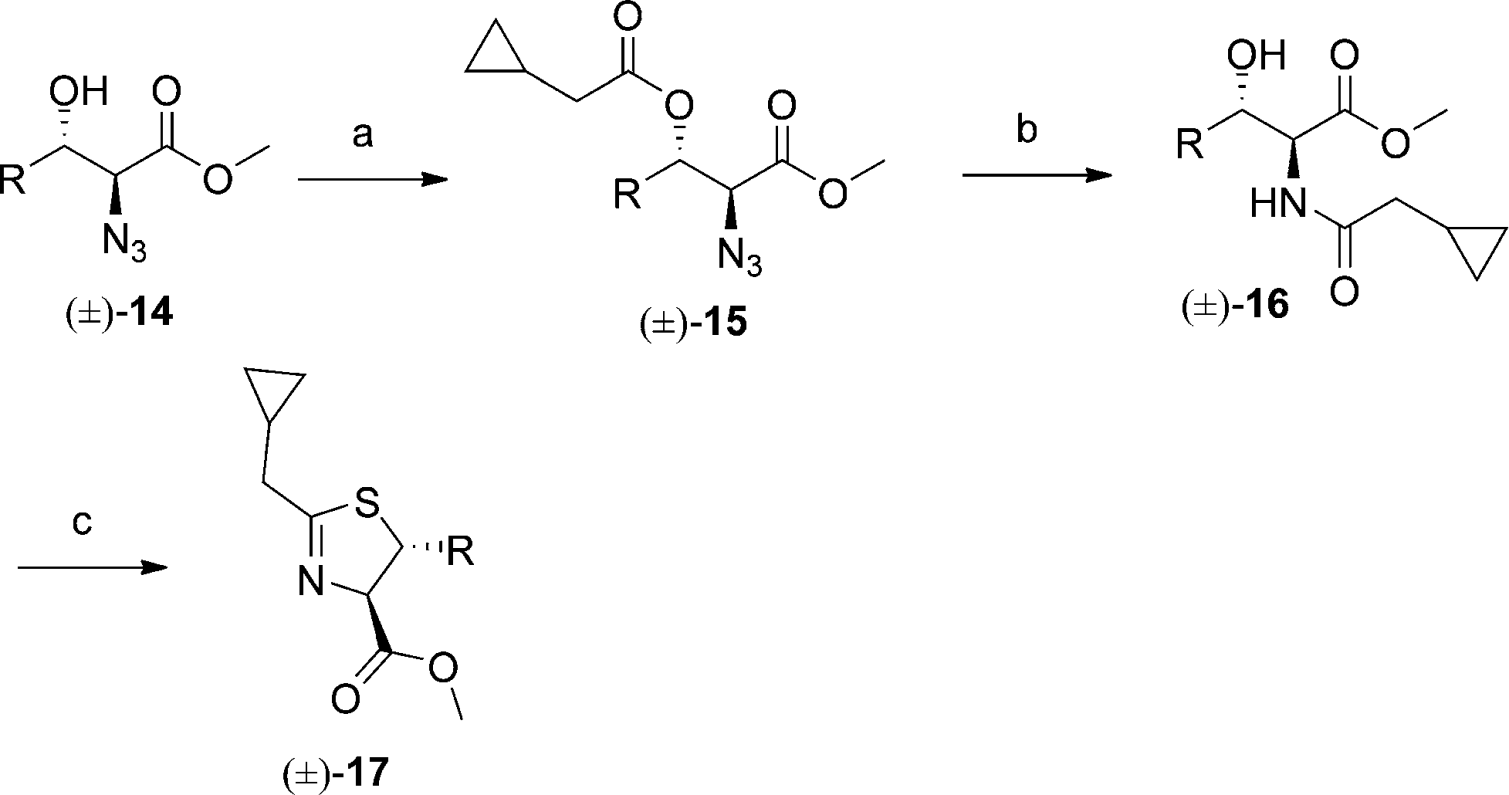

Entry	R	15	Yield [%]	**16**	Yield [%]	**17**	Yield [%]
1	*p*-FPh	(±)-**15a**	85	(±)-**16a**	83	(±)-**17a**	71
2	*m*-MeOPh	(±)-**15b**	94	(±)-**16b**	80	(±)-**17b**	80
3	Bn	(±)-**15c**	75	(±)-**16c**	77	(±)-**17c**	24
4	2-thienyl	(±)-**15d**	81	(±)-**16d**	78	(±)-**17d**	65

**Scheme 2:**
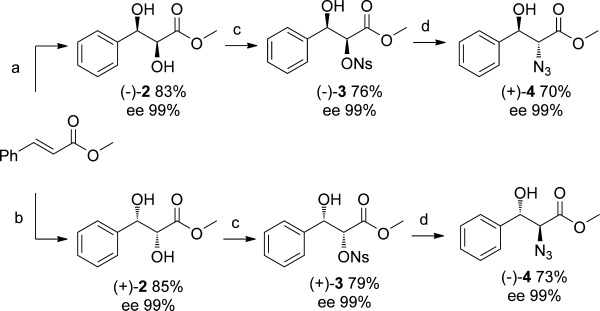
a) AD-mix-β, MeSO_2_NH_2_, *t*BuOH/H_2_O (1:1), RT; b) AD-mix-α, MeSO_2_NH_2_, *t*BuOH/H_2_O (1:1), RT; c) NsCl, TEA, CH_2_Cl_2_, 0°C; d) NaN_3_, DMF, 40°C.

After establishing the synthetic route required to access the target Δ^2^-thiazolines racemically, we prepared the enantioselective synthesis. Methyl cinnamate was first oxidized by Sharpless asymmetric dihydroxylation using commercially available AD α- or β-mixes (Scheme 2). Treatment of the enatiomerically pure dihydroxy compounds (−)-**2** and (+)-**2** with NsCl and TEA followed by NaN_3_ displacement gave the enantiomerically pure azido alcohols (+)-**4** and (−)-**4**, respectively (Scheme 2).

**Scheme 3:**
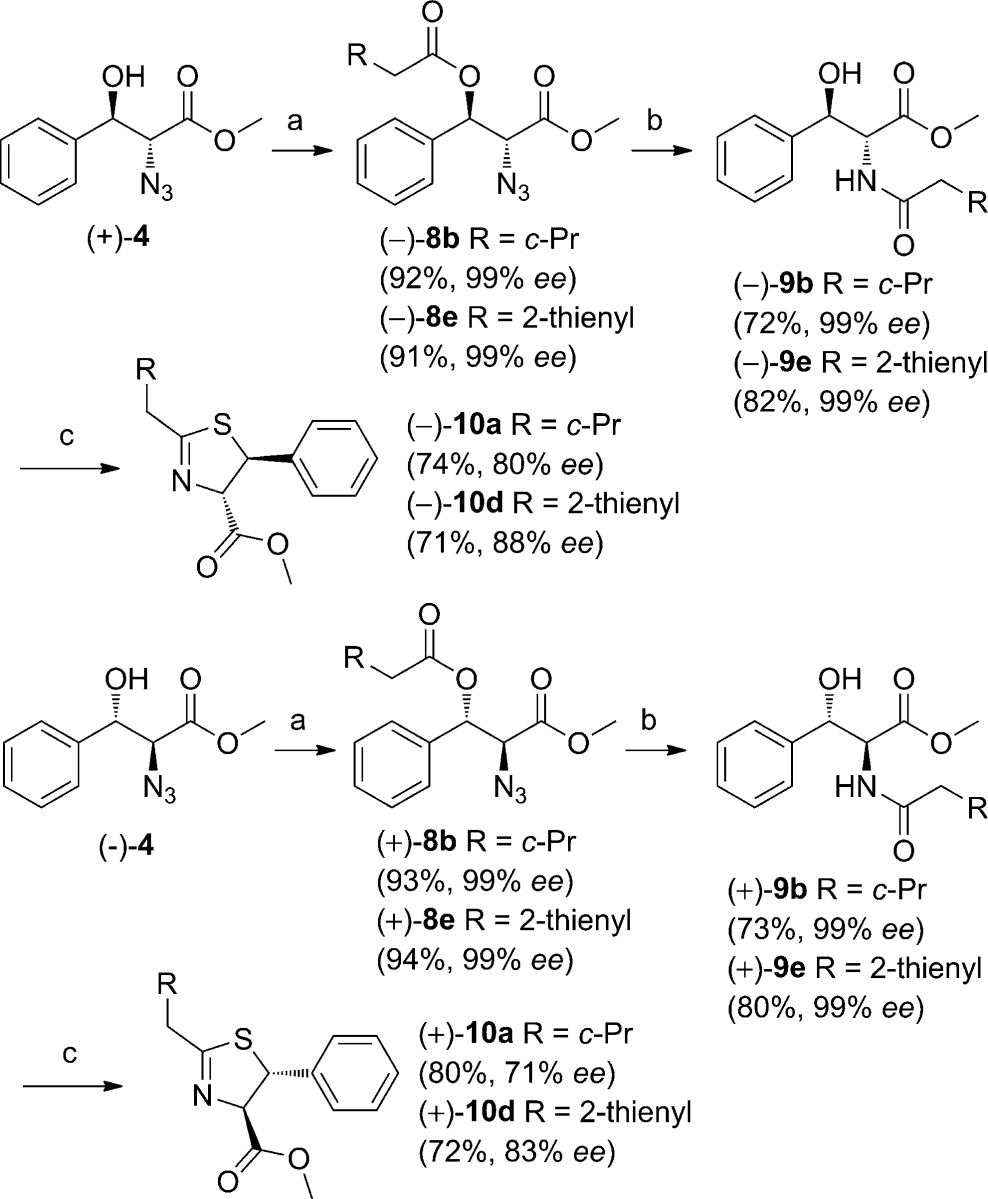
a) RCH_2_COOH, DCC, DMAP, CH_2_Cl_2_, RT; b) i) SnCl_2_⋅2H_2_O, RT, 2 h; ii) NaHCO_3_, MeOH/H_2_O or 1,4-dioxane/H_2_O, RT, 18 h; c) the Lawesson reagent, toluene, reflux; the *ee* was determined by chiral chromatography.

Azido alcohols (+)-**4** and (−)-**4** were converted into the azido esters (−)-**8b**,**e** and (+)-**8b**,**e**, respectively, by the Steglich esterification procedure. This was followed by a SnCl_2_**⋅**2H_2_O-induced azide reduction/O→N acyl migration reaction to give amido alcohols (−)-**9b**,**e** and (+)-**9b**,**e** in 72–82% yield and excellent enantiomeric excess (99%; Scheme 3). After LR-induced ring-closure, the 5-phenyl-substituted Δ^2^-thiazolines (−)-**10a**,**d** and (+)-**10a**,**d** were isolated in 71–80% yields and 71–88% *ee* (Scheme 3).

**Scheme 4:**
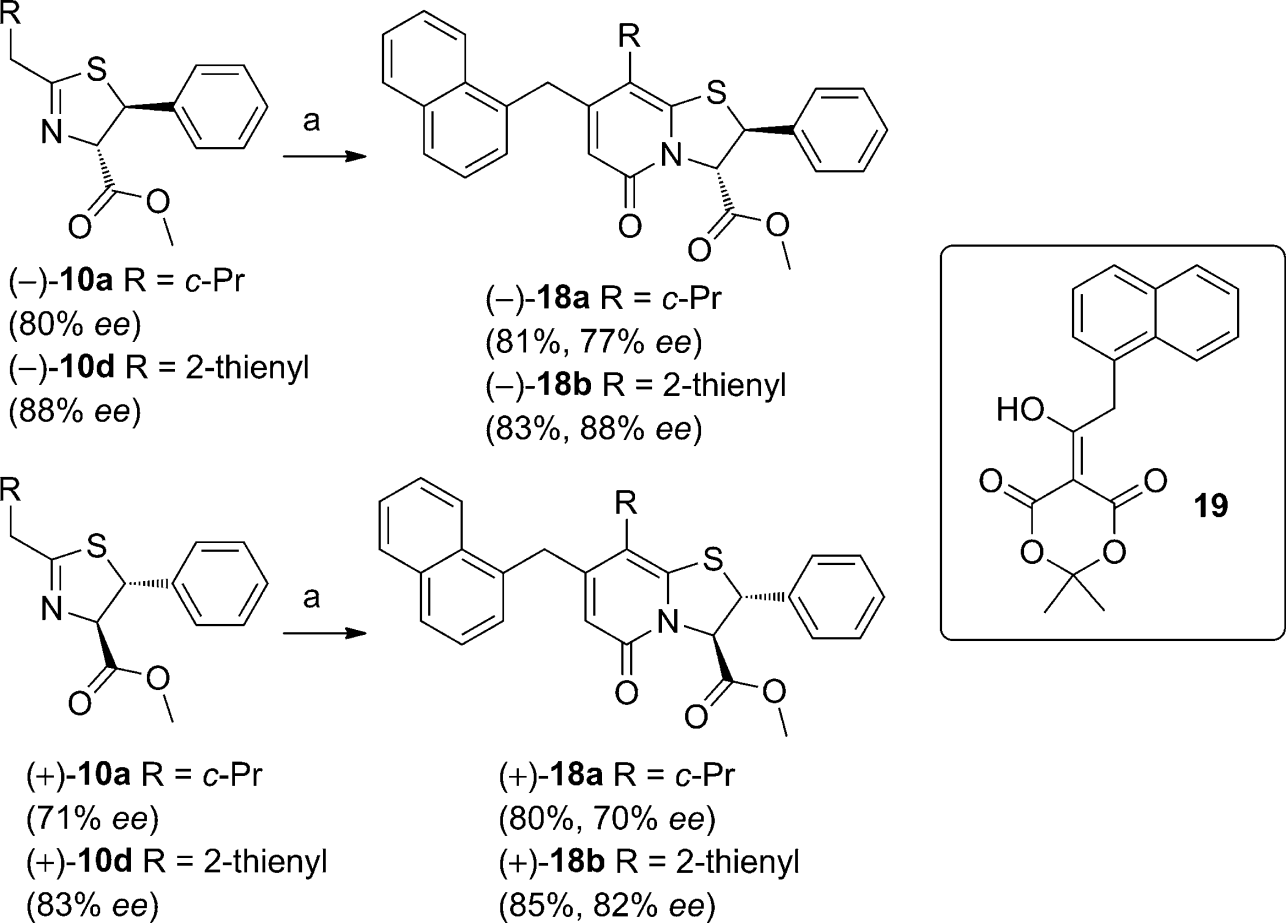
a) Trifluoroacetic acid (TFA), 19, microwave irradiation, 140°C, 2 min, dichloroethene (DCE); the *ee* was determined by chiral chromatography.

We used the enantiomerically enriched thiazolines (+)-**10a**,**d** and (−)-**10a**,**d** to synthesize the previously mentioned biologically active 2-pyridones (Structures **I** and **II**, Figure 2) by an acyl/ketene cyclocondensation reaction with the acyl Meldrum’s acid derivative **19**^22^ (Scheme 4). This gave the enantiomerically enriched 2-phenyl-substituted thiazolino ring-fused 2-pyridones (+)-**18a**,**b** and (−)-**18a**,**b** in 80–85% yields and 70–88% *ee* (Scheme 4). Hydrolyses of compounds **18a**,**b** have previously been reported by our group.20c,^23^

Epimerization of the stereochemistry occurred in the ring-closing reaction with LR (e.g., Scheme 3, (−)-**9b**, 99 % *ee*→(−)-**10a**, 80 % *ee*). A series of experiments to investigate the influence of LR stoichiometry on the reaction yield and *ee* was therefore performed. Use of less LR resulted in a decreased yield and *ee* of the product (Table 5, entry 1). Conversely, increasing the amount of LR to 1 equivalent significantly improved both the yield (90 %) and the *ee* of the reaction (Table 5, entry 4). Reports on this type of 2,4,5-trisubstituted Δ^2^-thiazoline containing an ester substituent in the 4-position are scarce in the literature.11c, 15a, 17 To the best of our knowledge, this is the first reported direct cyclization of amido alcohols to Δ^2^-thiazolines containing an ester in the 4-position and an aryl/heteroaryl in the 5-position with LR.

**Table 5:** Effect of varying the amount of the Lawesson reagent.
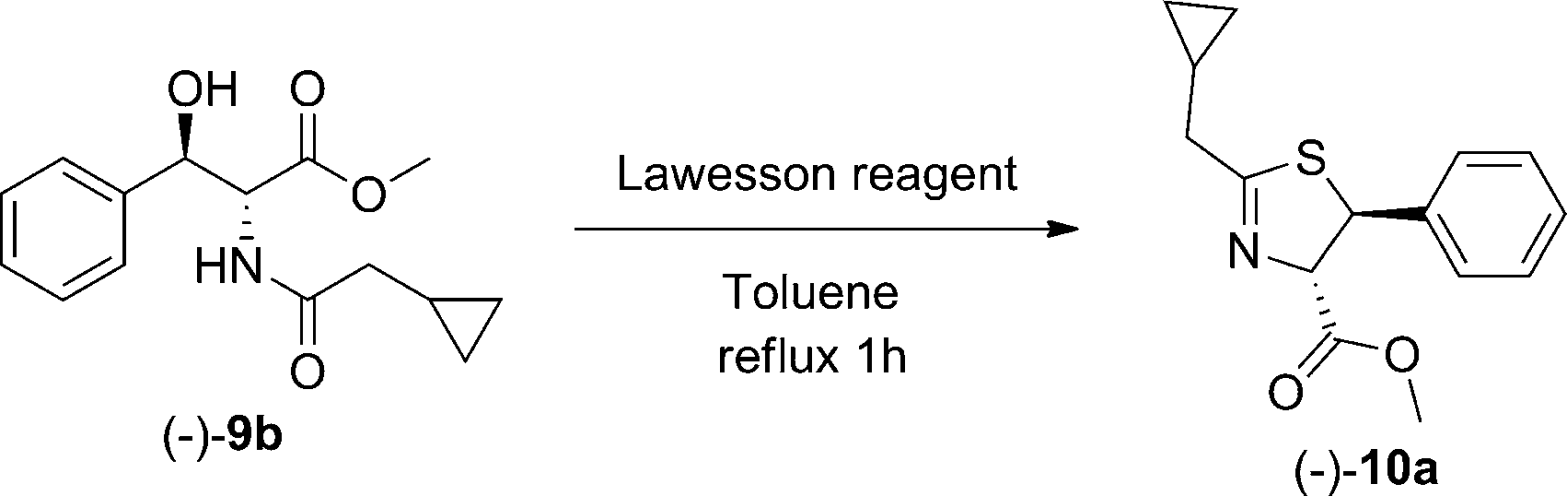

Entry	Amount LR [equiv]	*T*	Yield [%]	*ee* [%]^a^
1	0.5	reflux^b^	50	65
2	0.6	RT–reflux^c^	75	73
3	0.6	reflux^b^	78	75
4	1.0	reflux^b^	90	97

[a] Determined by chiral HPLC on a Whelk-O1 column.

[b] The oil bath was preheated before the reaction was started.

[c] The reaction was heated to reflux starting from RT; the reflux was then continued for 1 h.

**Scheme 5:**
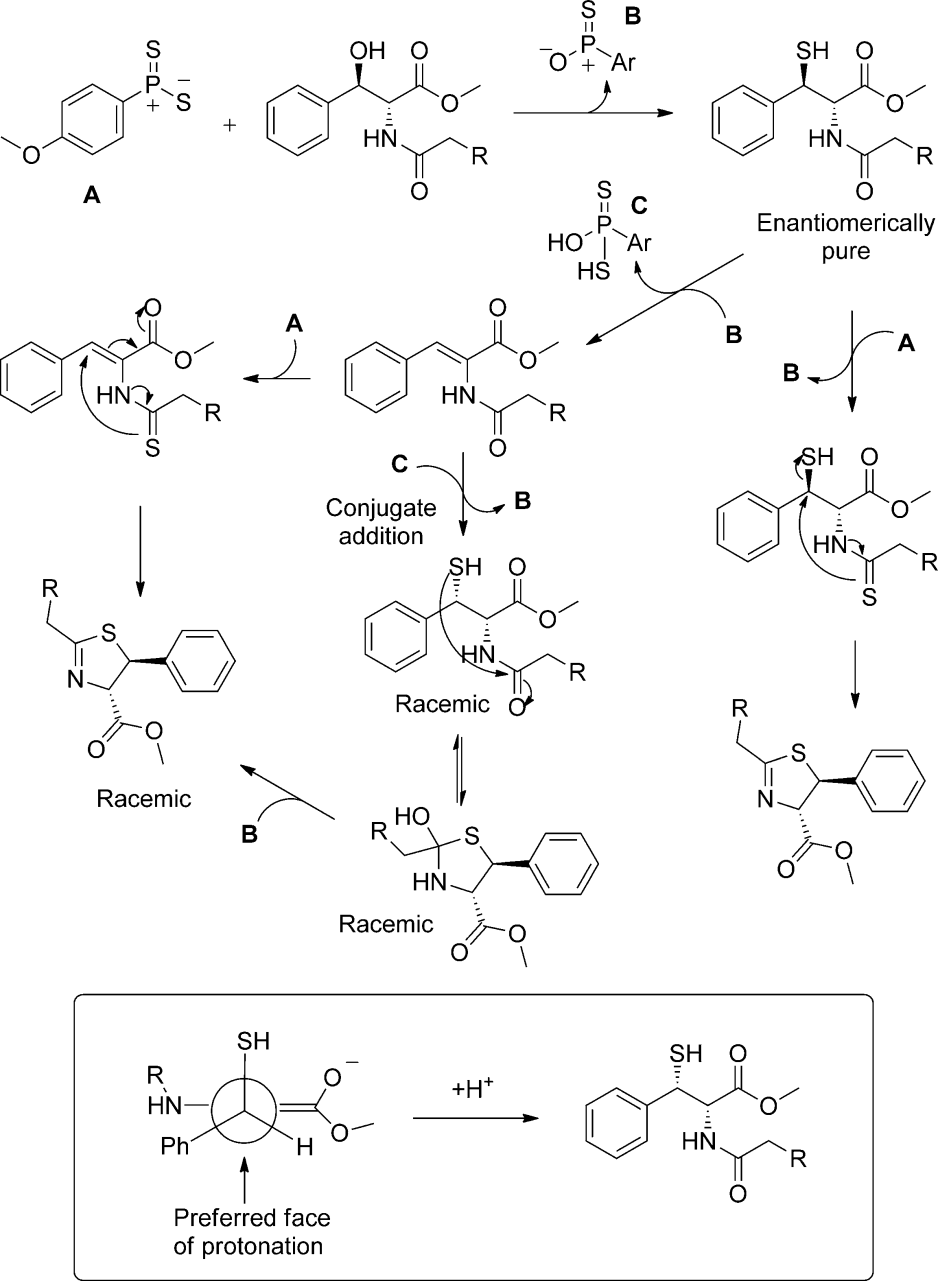
Possible mechanism for Δ^2^-thiazoline formation with the Lawesson reagent.

In addition, these data support the proposed mechanism for the formation of Δ^2^-thiazolines with LR published by Nishio,11b in which the initial thiolation of the hydroxy compound is known to occur with retention of configuration (Scheme 5).^27^ The resultant thiol amide can either undergo a second thiolation reaction of the amide with another equivalent of LR (to form the enantiomerically pure thiol thio amide) or a *syn*-elimination facilitated by compound **B** (Scheme 5). In the latter case, a Michael acceptor is formed that can react further with a sulfur nucleophile (i.e., compound **C**, Scheme 5) through conjugate addition and form the racemic thiol amide. Conjugate addition reactions of thiols to trisubstituted α,β-unsaturated carbonyl compounds are known to precede in high diastereoselectivities with (*E*)-olefins even at elevated temperatures.^28^ This selectivity is explained by the selective protonation of the enolate intermediate (shown in the inset in Scheme 5) due to stereoelectronic effects of the sulfur substituent.^28^ The α,β-unsaturated ester formed may also undergo a thiolation reaction with LR, and subsequent cyclization to the racemic *trans*-5-substituted 2-thiazoline. Although this 5-*endo*-*trig* cyclization is disfavored for oxygen and nitrogen nucleophiles, it is known to proceed for sulfur-based nucleophiles.^29^ Carbocationic intermediates have also been reported with LR and amido alcohols, but only in the presence of the strongly cation-stabilizing ferrocene group.11c,^30^

## Conclusion

We have developed scalable and robust synthetic pathways to highly enantiomerically enriched 5-substituted Δ^2^-thiazolines, which can be used in a ketene/imine cyclocondensation reaction to yield 2-substituted ring-fused 2-pyridones. The absolute stereochemistry was initially set through a Sharpless asymmetric dihydroxylation of methyl cinnamate. The subsequent nosylation, NaN_3_ displacement, esterification, and azide reduction/O→N acyl migration reactions all proceeded in good yields and without detectable epimerization on a gram scale. The final ring-closure of the amido alcohols proceeded in good yields with only 0.6 equivalents of LR, albeit with some epimerization. Increasing the amount of LR to 1 equivalent in this reaction ameliorated this problem, and afforded Δ^2^-thiazolines in excellent yield and stereochemical purity. The established route proved effective in the synthesis of aryl/heteroaryl 5-substituted Δ^2^-thiazolines and provides facile access to interesting unnatural analogues of cysteine and serine. Ultimately, this methodology also opens up synthetic possibilities for making asymmetric 5-substituted Δ^2^-thiazoline-containing ligands, as well as analogues of Δ^2^-thiazoline based natural products.

## Experimental Section

**General**: Unless stated otherwise, all reagents and solvents were used as received from commercial suppliers. DMF and THF were dried in a solvent drying system (THF drying agent: neutral alumina; DMF drying agent: activated molecular sieves, also equipped with an isocyanate scrubber) and were collected fresh prior to every reaction. NaH was prewashed with pentane, dried under vacuum, and stored in a dessicator. Preparatory HPLC was performed on a C18 reversed-phase column (25 cm×21.2 mm, 5 μm) with H_2_O/MeCN mixtures as the eluent. Chiral chromatography was performed on either an (*S*,*S*)-Whelk-O1 chiral column with hexane/CH_2_Cl_2_/alcohol as the eluents or with supercritical fluid chromatography on a Chiralpak-AD or Lux C4 chiral column with CO_2_/MeOH mixtures as the eluents. Microwave reactions were performed in a microwave reactor; temperatures were monitored with an IR probe. TLC was performed on silica gel and detected with UV light. Column chromatography was employed on normal phase silica gel (eluents given in brackets). Optical rotation was measured with a polarimeter at 20°C and 589 nm. IR was recorded on a spectrometer equipped with an ATR device. ^1^H and ^13^C NMR spectra were recorded on a 400 MHz spectrometer at 298 K and calibrated by using the residual peak of the solvent as the internal standard (CDCl_3_: CHCl_3_ *δ*_H_=7.26 ppm, CDCl_3_ *δ*_C_=77.16 ppm; [D_6_]DMSO: [D_5_]DMSO *δ*_H_=2.5 ppm, [D_6_]DMSO *δ*_C_=39.5 ppm). HRMS was performed by using a mass spectrometer with electrospray ionization (ES^+^); sodium formate was used as the calibration chemical.

### Representative procedures

*(±)-(2*S*,3*R*)-2,3-Dihydroxy-3-phenylpropionic acid methyl ester ((±)-**2**)*: Methyl cinnamate (30.83 mmol, 5 g), K_2_OsO_4_**⋅**2H_2_O (0.62 mmol, 228 mg), and 4-methylmorpholine *N*-oxide (46.25 mmol, 5.42 g) were mixed in acetone/MeCN/H_2_O (1:1:1, 140 mL) and the reaction was stirred at RT for 16 h. The volatile solvents were removed by evaporation and the remaining mixture was diluted with saturated aqueous NaHCO_3_ and extracted with EtOAc. The organic phase was dried (Na_2_SO_4_), filtered, and concentrated. The crude material was purified by column chromatography on silica gel (heptane/EtOAc 80:20→50:50) to give (±)-**2** as a colorless oil (4.9 g, 81%). Spectral data agreed with published results.^31^

*(±)-(2*S*,3*R*)-3-Hydroxy-2-(4-nitrobenzenesulfonyloxy)-3-phenylpropionic acid methyl ester ((±)-**3**)*: Compound (±)-**2** (26.25 mmol, 5.15 g) was dissolved in CH_2_Cl_2_ (250 mL) and cooled to 0°C in an ice bath. 4-Nitrobenzenesulfonyl chloride (26.25 mmol, 5.82 g) followed by TEA (26.25 mmol, 3.66 mL) were added and the reaction was stirred at 0°C for 1 h. The reaction mixture was acidified and then washed with 1m aqueous HCl (pH≈1), dried (Na_2_SO_4_), filtered, and concentrated. The crude material was purified by column chromatography on silica gel (heptane/EtOAc 90:10→60:40) to give (±)-**3** as a colorless solid (7.5 g, 75%). Spectral data agreed with published results.^25^

*(±)-(2*R*,3*R*)-2-Azido-3-phenyl-3-hydroxypropionic acid methyl ester ((±)-**4**)*: Compound (±)-**3** (7.55 mmol, 2.88 g) and NaN_3_ (45.3 mmol, 2.94 g) were mixed in dry DMF (13 mL) and the reaction was heated to 40°C for 48 h. The reaction mixture was diluted with brine and extracted with EtOAc, the organic phase was dried (Na_2_SO_4_), filtered, and concentrated. The crude material was purified by column chromatography on silica gel (heptane/EtOAc 90:10→60:40) to give (±)-**4** as a colorless noncrystalline solid (1.17 g, 70%). ^1^H NMR (400 MHz, CDCl_3_): *δ*=7.42–7.31 (m, 5H), 5.00 (d, *J*=7.2 Hz, 1H), 4.10 (d, *J*=7.2 Hz, 1H), 3.77 (s, 3H), 3.02 ppm (brs, 1H); ^13^C NMR (100 MHz, CDCl_3_): *δ*=169.5, 139.0, 128.9, 128.7 (2C), 126.7 (2C), 74.2, 66.9, 52.9 ppm; IR: 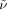
=3426, 2117, 1738 cm^−1^; HRMS (ES) calcd for C_10_H_11_N_3_NaO_3_: 244.0698 [*M*+Na]^+^; found: 224.0701.

*(±)-(2*R*,3*R*)-2-*tert*-Butoxycarbonylamino-3-hydroxy-3-phenylpropionic acid methyl ester ((±)-**5**)*: SnCl_2_**⋅**2H_2_O (22.6 mmol, 5.1 g) was dissolved in 1,4-dioxane/H_2_O (1:4, 20 mL) and (±)-**4** (4.52 mmol, 1 g) dissolved in 1,4-dioxane (20 mL) was added. The reaction was stirred at RT for 4 h. NaHCO_3_ (4.6 mmol, 386 mg) was added, followed by Boc_2_O (0.35 mmol, 76 mg), and the reaction was stirred at RT for an additional 16 h. The reaction mixture was filtered through Celite, and washed with EtOAc. The organic phase was washed with saturated NaHCO_3_, dried (Na_2_SO_4_), filtered, and concentrated. The crude material was purified by column chromatography on silica gel (heptane/EtOAc 90:10→60:40) to give (±)-**5** as a colorless noncrystalline solid (1.24 g, 93%). ^1^H NMR (400 MHz, CDCl_3_): *δ*=7.39–7.24 (m, 5H), 5.34 (d, *J*=7.2 Hz, 1H), 5.18 (d, *J*=2.8 Hz, 1H), 4.77–4.66 (m, 1H), 3.70 (s, 3H), 1.45 ppm (s, 9H); ^13^C NMR (100 MHz, CDCl_3_): *δ*=170.5, 156.4, 139.3, 128.4 (2 C), 128.1, 126.1 (2C), 80.7, 75.1, 59.8, 52.5, 28.3 ppm (3 C); IR: 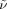
=3425, 1744, 1713, 1502, 1368 cm^−1^; HRMS (ES) calcd for C_15_H_21_NNaO_5_: 318.1317 [*M*+Na]^+^; found: 318.1318.

*(±)-(2*S*,3*S*)-3-Acetylsulfanyl-2-*tert*-butoxycarbonylamino-3-phenylpropionic acid methyl ester ((±)-**6**)*: Compound (±)-**5** (0.68 mmol, 200 mg) was dissolved in CH_2_Cl_2_ (8 mL) and cooled to 0°C in an ice bath. Ms_2_O (0.75 mmol, 131 mg) followed by TEA (1.02 mmol, 0.14 mL) were added and the reaction was stirred at 0°C for 2 h. The reaction mixture was diluted with CH_2_Cl_2_ and washed with saturated aqueous NH_4_Cl. The organic phase was dried (Na_2_SO_4_), filtered, and concentrated. The crude material was dissolved in dry DMF (8 mL) and potassium thioacetate (3.4 mmol, 388 mg) was added; the reaction was stirred at RT for 15 h. The reaction mixture was quenched with 1m aqueous HCl and acidified to pH≈1, then extracted with EtOAc. The organic phase was dried (Na_2_SO_4_), filtered, and concentrated. The crude material was purified by column chromatography on silica gel (heptane/EtOAc 95:5→80:20) to give (±)-**6** as a yellow noncrystalline solid (145 mg, 60 %). ^1^H NMR (400 MHz, CDCl_3_): *δ*=7.34–7.22 (m, 5H), 5.27 (d, *J*=9.2 Hz, 1H), 5.00 (d, *J*=7.2 Hz, 1H), 4.75 (dd, *J*=9.2, 7.2 Hz, 1H), 3.59 (s, 3H), 2.33 (s, 3H), 1.41 ppm (s, 9H); ^13^C NMR (100 MHz, CDCl_3_): *δ*=194.0, 170.8, 155.2, 137.6, 128.8 (2C), 128.3 (2C), 128.2, 80.4, 58.2, 52.5, 50.4, 30.5, 28.4 ppm (3C); IR: 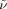
=3368, 1747, 1718, 1498, 1164 cm^−1^; HRMS (ES) calcd for C_17_H_23_NNaO_5_S: 376.1195 [*M*+Na]^+^; found: 376.1196.

*(±)-(2*R*,3*R*)-2-Azido-3-(2-cyclopropylacetoxy)-3-phenylpropionic acid methyl ester ((±)-**8b**)*: Compound (±)-**4** (0.90 mmol, 200 mg), cyclopropylacetic acid (1.18 mmol, 0.11 mL), and dicyclohexylcarbodiimide (1.18 mmol, 242 mg) were mixed in CH_2_Cl_2_ (8 mL) and DMAP (1.18 mmol, 144 mg) was added; the reaction was stirred at RT for 1 h. The reaction mixture was diluted with CH_2_Cl_2_ and washed with 2% aqueous KHSO_4_. The organic phase was dried (Na_2_SO_4_), filtered, and concentrated. The crude material was purified by column chromatography on silica gel (heptane/EtOAc 95:5→85:15) to give (±)-**8b** as a colorless oil (258 mg, 95%). ^1^H NMR (400 MHz, CDCl_3_): *δ*=7.41–7.32 (m, 5H), 6.16 (d, *J*=6.4 Hz, 1H), 4.35 (d, *J*=6.4 Hz, 1H), 3.76 (s, 3H), 2.28 (d, *J*=7.2 Hz, 2H), 1.11–0.98 (m, 1H), 0.59–0.51 (m, 2H), 0.20–0.13 ppm (m, 2H); ^13^C NMR (100 MHz, CDCl_3_): *δ*=171.5, 167.8, 135.4, 129.2, 128.7 (2C), 127.2 (2C), 74.5, 65.4, 52.8, 39.4, 6.8, 4.5 ppm (2C); IR: 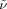
=2118, 1750 cm^−1^: HRMS (ES) calcd for C_15_H_17_N_3_NaO_4_: 326.1117 [*M*+Na]^+^; found: 326.1115.

*(±)-(2*R*,3*R*)-2-(2-Cyclopropylacetylamino)-3-hydroxy-3-phenylpropionic acid methyl ester ((±)-**9b**)*: SnCl_2_**⋅**2H_2_O (2.25 mmol, 508 mg) was dissolved in MeOH/H_2_O 1:4 (2.5 mL) and (±)-**8b** (0.45 mmol, 135 mg) dissolved in MeOH (2.5 mL) was added and the reaction was stirred at RT for 2 h. NaHCO_3_ (9 mmol, 756 mg) was added in portions to the reaction mixture and the reaction was stirred at RT for an additional 18 h. The reaction mixture was filtered through Celite and the Celite was carefully washed with EtOAc. The organic phase was washed with saturated NaHCO_3_, dried (Na_2_SO_4_), filtered, and concentrated. The crude material was purified by column chromatography on silica gel (heptane/EtOAc 80:20→50:50) to give (±)-**9b** as a colorless noncrystalline solid (89 mg, 71%). ^1^H NMR (400 MHz, CDCl_3_): *δ*=7.27–7.19 (m, 3H), 7.18–7.13 (m, 2H), 6.66–6.58 (m, 1H), 5.18 (d, *J*=3.6 Hz, 1H), 4.97–4.92 (m, 1H), 4.45 (brs, 1H), 3.65 (s, 3H), 2.07 (d, *J*=7.6 Hz, 1H), 0.86–0.76 (m, 1H), 0.52–0.42 (m, 2H), 0.09–0.00 ppm (m, 2H): ^13^C NMR (100 MHz, CDCl_3_): *δ*=174.2, 170.1, 139.2, 128.3 (2C), 128.1, 126.0 (2C), 75.1, 59.1, 52.6, 41.0, 6.9, 4.6 ppm (split, 2C); IR: 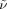
=3419, 1743, 1656, 1521 cm^−1^: HRMS (ES) calcd for C_15_H_19_NNaO_4_: 300.1212 [*M*+Na]^+^; found: 300.1211.

*(±)-(4*S*,5*S*)-2-Cyclopropylmethyl-5-phenyl-4,5-dihydrothiazole-4-carboxylic acid methyl ester ((±)-**10a**)*: Compound (±)-**9b** (0.46 mmol, 126 mg) and 0.6 (0.28 mmol, 113 mg) or 1 equivalent (0.46 mmol, 186 mg) of the Lawesson reagent were mixed in toluene (3 mL) and the reaction was heated at reflux with an oil bath for 1 h. The reaction mixture was diluted with EtOAc and washed with saturated aqueous NaHCO_3_. The organic phase was dried (Na_2_SO_4_), filtered, and concentrated. The crude material was purified by column chromatography on silica gel (heptane/EtOAc 80:20→50:50) to give (±)-**10a** as a colorless oil (with 0.6 equivalents of the Lawesson reagent: 90 mg, 71%; with 1 equivalent of the Lawesson reagent: 114 mg, 90%). ^1^H NMR (400 MHz, CDCl_3_): *δ*=7.26–7.13 (m, 5H), 5.21 (d, *J*=6.8 Hz, 1H), 5.05–5.01 (m, 1H), 3.68 (s, 3H), 2.45–2.40 (m, 2H), 1.00–0.88 (m, 1H), 0.53–0.45 (m, 2H), 0.20–0.12 ppm (m, 2H); ^13^C NMR (100 MHz, CDCl_3_): *δ*=174.9, 170.9, 140.6, 129.0 (2C), 128.2, 127.6 (2C), 85.9, 56.6, 52.9, 39.4, 9.4, 5.2, 5.1 ppm; IR: 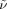
=1744, 1619, 1266 cm^−1^; HRMS (ES) calcd for C_15_H_17_NNaO_2_S: 298.0878 [*M*+Na]^+^; found: 298.0875.

trans*-3-(3-Methoxyphenyl)acrylic acid methyl ester (**11b**)*: Trimethyl phosphonoacetate (8.20 mmol, 1.33 mL) was dissolved in dry THF (40 mL) and NaH (9.02 mmol, 216 mg) was added; the reaction was stirred at RT for 5 min before 3-methoxybenzaldehyde (4.10 mmol, 0.5 mL) was added. The stirring continued at RT for a further 15 h. THF was evaporated and the crude mixture was diluted with saturated aqueous NaHCO_3_ and extracted with EtOAc. The organic phase was dried (Na_2_SO_4_), filtered, and concentrated. The crude material was purified by column chromatography on silica gel (heptane/EtOAc 95:5→70:30) to give **11b** as a colorless oil (755 mg, 96%). Spectral data agreed with published results.^32^

*(2*S*,3*R*)-2,3-Dihydroxy-3-phenylpropionic acid methyl ester ((−)-**2**)*: Methyl cinnamate (12.32 mmol, 2 g), AD-mix-β (16.48 g) and methylsulfonamide (12.32 mmol, 1.17 g) were mixed in *t*BuOH/H_2_O (1:1, 40 mL) and the reaction was stirred at RT for 18 h. The reaction mixture was diluted with water, extracted with EtOAc, and the combined organic phases were dried (Na_2_SO_4_), filtered, and concentrated. The crude material was purified by column chromatography on silica gel (heptane/EtOAc 80:20→50:50) to give (−)-**2** as a colorless solid (2 g, 83%). [*α*] 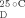 (*c*=4.0 in CHCl_3_); spectral data agreed with published results.^31^

*(2*S*,3*S*)-7-Naphthalen-1-ylmethyl-5-oxo-2-phenyl-8-thiophen-2-yl-2,3-dihydro-5*H*-thiazolo[3,2-*a*]pyridine-3-carboxylic acid methyl ester ((−)-**18b**)*: Compound (−)-**10d** (0.23 mmol, 72 mg), **19** (0.58 mmol, 181 mg), and trifluoroacetic acid (TFA, 0.23 mmol, 18 μL) were mixed in 1,2-dichloroethane (1 mL) and the reaction was heated in a microwave oven at 140°C for 2 min. The reaction mixture was diluted with CH_2_Cl_2_ and washed with saturated aqueous NaHCO_3_. The organic phase was dried (Na_2_SO_4_), filtered, and concentrated. The crude material was purified by column chromatography on silica gel (heptane/EtOAc 90:10→50:50) to give (−)-**18b** as a yellow foam (97 mg, 83%). [*α*] 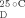 =−113 (*c*=2.0 in CHCl_3_); ^1^H NMR (400 MHz, CDCl_3_): *δ*=7.88–7.82 (m, 1H), 7.79–7.69 (m, 2H), 7.49–7.43 (m 2H), 7.43–7.30 (m, 7H), 7.28–7.23 (m, 1H), 7.09–7.05 (m, 2H), 5.82 (s, 1H), 5.60 (d, *J*=3.2 Hz, 1H), 5.00 (d, *J*=3.2 Hz, 1H), 4.19–4.07 (m, 2H), 3.84 ppm (s, 3H); ^13^C NMR (100 MHz, CDCl_3_): *δ*=168.2, 161.2, 155.6, 149.0, 138.4, 136.5, 134.0, 133.7, 131.9, 129.4 (2C), 129.3, 129.0, 128.8, 128.0, 127.9, 127.5 (2C), 126.8 (2C), 126.3, 125.8, 125.6, 123.9, 115.5, 108.1, 71.2, 53.5, 50.9, 37.0 ppm; IR: 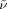 =1752, 1656, 1265 cm^−1^; HRMS (ES) calcd for C_30_H_23_NNaO_3_S_2_: 532.1017 [*M*+Na]^+^; found: 532.1020.

## Notes

As a service to our authors and readers, this journal provides supporting information supplied by the authors. Such materials are peer reviewed and may be re-organized for online delivery, but are not copy-edited or typeset. Technical support issues arising from supporting information (other than missing files) should be addressed to the authors.
