# Supplementary material for: Asymmetric Synthesis of 2,4,5-Trisubstituted Δ2-Thiazolines
Source: Chemistry. 2013 Jun 17;19(30):9916–22. doi: 10.1002/chem.201301120 (PMC3784040; doi:10.1002/chem.201301120)
Supplement: Supplementary file 1 [file chem0019-9916-SD1.pdf]

CHEMISTRY
A EUROPEAN JOURNAL

Supporting Information

© Copyright Wiley-VCH Verlag GmbH & Co. KGaA, 69451 Weinheim, 2013

Asymmetric Synthesis of 2,4,5-Trisubstituted Δ^2-Thiazolines

Christoffer Bengtsson,[a] Hanna Nelander,[b] and Fredrik Almqvist*[a, c, d]

chem_201301120_sm_miscellaneous_information.pdf

Supporting information

Table of Contents

Synthesis and spectral data of new compounds……………………….p2-18

^{1}H- and ^{13}C-NMR of new compounds……………………..…...p19-57

Chiral chromatograms……………….………………………...…p58-81

Chiral chromatogram, ^{1}H-, ^{13}C-NMR of (-)-**10a**
when using 1 eq of Lawessons reagent……………………….…p82-83

References……………………………………………………….p84

Compounds **11a**[1], **11b**[2], **11c**[3], **11d**[4], *rac*-**12a**[5], *rac*-**12c**[6] were made according to their respective general sythesis procedure, data in agreement with previously published results

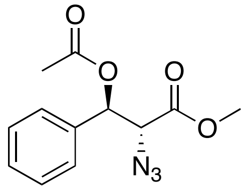

(±)-(2R, 3R)-3-Acetoxy-2-azido-3-phenyl-propionic acid methyl ester (8a): (±)-**4** (0.45mmol, 100mg) and Ac_2O (0.59mmol, 56 µl) were dissolved in DCM and DMAP (0.59mmol, 72mg) was added and the reaction was stirred att rt for 1 h. The reaction mixture was diluted with DCM and washed with 2% $KHSO_4$ (aq). The organic phase was dried (Na_2SO_4), filtered and concentrated. The crude material was purified by column chromatography on silica gel (heptane:EtOAc 95:5 → 85:15) to give **8a** as a colorless oil (114mg, 96%) ^{1}H-NMR (400 MHz, $CDCl_3$) δ 7.42-7.32 (m, 5H), 6.13 (d, ***J*** = 6.4 Hz, 1H), 4.35 (d, ***J*** = 6.4 Hz, 1H), 3.76 (s, 3H), 2.12 (s, 3H) ^{13}C-NMR (100 MHz, $CDCl_3$) δ 169.3, 167.8, 135.3, 129.2, 128.7 (2C), 127.3 (2C), 74.5, 65.2, 52.9, 20.9 IR λ 2119, 1752 cm^{-1} HRMS (ES) calcd [M+Na] for $C_{12}H_{13}N_3NaO_4$ 286.0804, obsd 286.0802

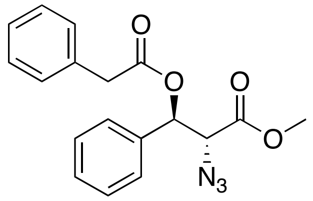

(±)-(2R, 3R)-2-Azido-3-phenyl-3-phenylacetoxy-propionic acid methyl ester ((±)-8c): Following the general procedure starting from 100mg of (±)-**4** gave 145mg of (±)-**8c** as a colorless oil (95% yield) ^{1}H-NMR (400 MHz, $CDCl_3$) δ 7.31-7.14 (m, 10H), 6.03 (d, ***J*** = 6.8 Hz, 1H), 4.22 (d, ***J*** = 6.8 Hz, 1H), 3.61 (s, 2H), 3.58 (s, 3H) ^{13}C-NMR (100 MHz, $CDCl_3$) δ 169.8, 167.7, 135.2, 133.3, 129.4 (2C), 129.3, 128.8 (2C), 128.7 (2C), 127.4, 127.3 (2C), 74.9, 65.3, 52.9, 41.4 IR λ 2118, 1750 cm^{-1} HRMS (ES) calcd [M+Na] for $C_{18}H_{17}N_3NaO_4$ 362.1117, obsd 362.1119

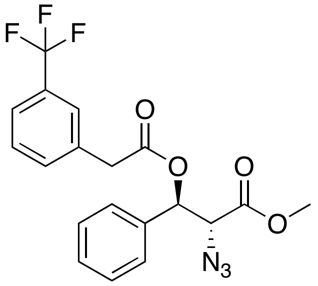

(±)-(2R, 3R)-2-Azido-3-phenyl-3-[2-(3-trifluoromethyl-phenyl)-acetoxy]-propionic acid methyl ester ((±)-8d): Following the general procedure starting from 100mg of (±)-**4** gave 168mg of (±)-**8c** as a colorless oil (90% yield) ^{1}H-NMR (400 MHz, $CDCl_3$) δ 7.58-7.53 (m, 2H), 7.48-7.44 (m, 2H), 7.37-7.33 (m, 3H), 7.33-7.27 (m, 2H), 6.13 (d, ***J*** = 6.8 Hz, 1H), 4.31 (d, ***J*** = 6.8 Hz, 1H), 3.75 (s, 2H), 3.68 (s, 3H) ^{13}C-NMR (100 MHz, $CDCl_3$) δ 169.1, 167.6, 135.0, 134.2, 132.9, 131.1 (q, ***J*** =

32 Hz, 1C), 129.4, 129.2, 128.8 (2C), 127.2 (2C), 126.3 (split, 1C), 124.3 (split, 1C), 124.1 (q, ***J*** = 271 Hz, 1C), 75.2, 65.1, 52.8, 41.0 IR λ 2118, 1751 cm^{-1} HRMS (ES) calcd [M+Na] for $C_{19}H_{16}F_3N_3NaO_4$ 430.0991, obsd 430.0994

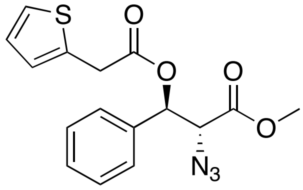

(±)-(2R, 3R)-2-Azido-3-phenyl-3-(2-thiophen-2-yl-acetoxy)-propionic acid methyl ester ((±)-**8e)**: Following the general procedure starting from 100mg of *rac*-**4** gave 142mg of (±)-**8c** as a colorless oil (91% yield) ^{1}H-NMR (400 MHz, $CDCl_3$) δ 7.39-7.31 (m, 5H), 7.24-7.21 (m, 1H), 6.98-6.93 (m, 2H), 6.14 (d, ***J*** = 6.8 Hz, 1H), 4.34 (d, ***J*** = 6.8 Hz, 1H), 3.95-3.85 (m, 2H), 3.71 (s, 3H) ^{13}C-NMR (100 MHz, $CDCl_3$) δ 168.8, 167.7, 135.0, 134.2, 129.4, 128.8 (2C), 127.4 (2C), 127.3, 127.0, 125.4, 75.2, 65.2, 53.0, 35.5 IR λ 2118, 1751 cm^{-1} HRMS (ES) calcd [M+Na] for $C_{16}H_{15}N_3NaO_4S$ 368.0681, obsd 368.0686

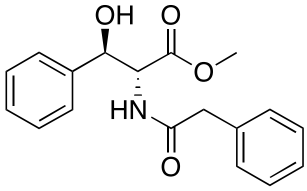

(±)-(2R, 3R)-3-Hydroxy-3-phenyl-2-phenylacetylamino-propionic acid methyl ester ((±)-**9c)**: Following the general procedure starting from 110mg of (±)-**8c** gave 71mg of (±)-**9c** as a colorless non crystalline solid (70% yield) ^{1}H-NMR (400 MHz, $CDCl_3$) δ 7.35-7.27 (m, 3H), 7.27-7.23 (m, 3H), 7.19-7.14 (m, 2H), 7.11-7.06 (m, 2H), 6.22-6.15 (m, 1H), 5.22-5.16 (m, 1H), 5.00-4.95 (m, 1H), 4.38 (brs, 1H), 3.69 (s, 3H), 3.61-3.51 (m, 2H) ^{13}C-NMR (100 MHz, $CDCl_3$) δ 172.6, 169.9, 138.9, 134.1, 129.4 (2C), 129.1 (2C), 128.4 (2C), 128.2, 127.6, 125.9 (2C), 75.0, 59.2, 52.7, 43.4 IR λ 3409, 1744, 1658, 1513 cm^{-1} HRMS (ES) calcd [M+Na] for $C_{18}H_{19}NNaO_4$ 336.1212, obsd 336.1212

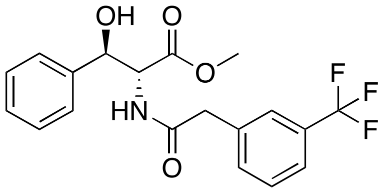

(±)-(2R, 3R)-3-Hydroxy-3-phenyl-2-[2-(3-trifluoromethyl-phenyl)-acetylamino]-propionic acid methyl ester ((±)-**9d)**: Following the general procedure starting from 160mg of (±)-**8d** gave 119mg of (±)-**9d** as a colorless non crystalline solid (80% yield) ^{1}H-NMR (400 MHz, $CDCl_3$) δ 7.58-7.52 (m, 1H), 7.48-7.41 (m, 2H), 7.41-7.35 (m, 1H), 7.28-7.22 (m, 3H), 7.13-7.06 (m, 2H), 6.26-

6.18 (m, 1H), 5.21 (d, ***J*** = 4.0 Hz, 1H), 5.00-4.96 (m, 1H), 3.71 (s, 3H), 3.64-3.54 (m, 2H) ^{13}C-NMR (100 MHz, $CDCl_3$) δ 171.4, 169.9, 138.8, 135.1, 132.9, 131.3 (q, ***J*** = 32 Hz, 1C), 129.5, 128.5 (2C), 128.3, 126.2 (split, 1C), 125.8 (2C), 124.4 (split, 1C), 124.0 (q, ***J*** = 271 Hz, 1C), 75.0, 59.3, 52.8, 42.9 IR λ 3307, 1743, 1654, 1544 cm^{-1} HRMS (ES) calcd [M+Na] for $C_{19}H_{18}F_3NNaO_4$ 404.1086, obsd 404.1089

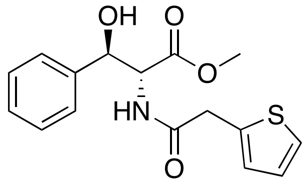

(±)-(2R, 3R)-3-Hydroxy-3-phenyl-2-(2-thiophen-2-yl-acetylamino)-propionic acid methyl ester ((±)-9e): Following the general procedure using 1,4-dioxane:H_2O as solvent starting from 140mg of (±)-**8e** gave 110mg of (±)-**9e** as a colorless oil (84% yield) ^{1}H-NMR (400 MHz, $CDCl_3$) δ 7.32-7.19 (m, 4H), 7.17-7.09 (m, 2H), 6.99-6.92 (m, 1H), 6.89-6.82 (m, 1H), 6.43-6.34 (m, 1H), 5.19 (d, ***J*** = 3.6 Hz, 1H), 5.00-4.94 (m, 1H), 3.76 (s, 2H), 3.68 (s, 3H) ^{13}C-NMR (100 MHz, $CDCl_3$) δ 171.2, 169.8, 138.9, 135.2, 128.4 (2C), 128.2, 127.6, 127.5, 125.9 (2C), 125.8, 74.9, 59.1, 52.7, 37.1 IR λ 3404, 1744, 1664, 1517 cm^{-1} HRMS (ES) calcd [M+Na] for $C_{16}H_{17}NNaO_4S$ 342.0776, obsd 342.0776

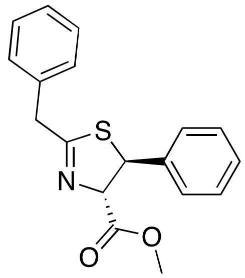

(±)-(4S, 5S)-2-Benzyl-5-phenyl-4,5-dihydro-thiazole-4-carboxylic acid methyl ester ((±)-10b): Following the general procedure starting from 110mg of (±)-**9c** using 0.6 eq of Lawesson's reagent gave 92mg of (±)-**10b** as a colorless oil (80% yield) ^{1}H-NMR (400 MHz, $CDCl_3$) δ 7.38-7.33 (m, 4H), 7.32-7.24 (m, 6H), 5.32 (d, ***J*** = 6.8 Hz, 1H), 5.20-5.16 (m, 1H), 4.02-3.91 (m, 2H), 3.81 (s, 3H) ^{13}C-NMR (100 MHz, $CDCl_3$) δ 173.6, 170.7, 140.2, 135.6, 129.2 (2C), 129.0 (2C), 128.8 (2C), 128.2, 127.5 (2C), 127.4, 86.0, 57.2, 52.9, 41.0 IR λ 1744, 1266 cm^{-1} HRMS (ES) calcd [M+Na] for $C_{18}H_{17}NNaO_2S$ 334.0878, obsd 334.0884

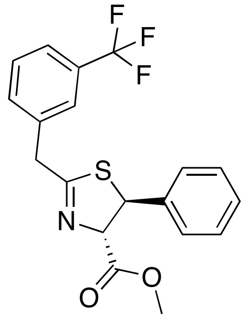

(±)-(4S, 5S)-5-Phenyl-2-(3-trifluoromethyl-benzyl)-4,5-dihydro-thiazole-4-carboxylic acid methyl ester ((±)-10c): Following the general procedure starting from 170 mg of (±)-**9d** using 0.6 eq of Lawesson's reagent gave 123 mg of (±)-**10c** as a colorless oil (72% yield) ^{1}H-NMR (400 MHz, $CDCl_3$) δ 7.66-7.61 (m, 1H), 7.59-7.52 (m, 2H), 7.51-7.44 (m, 1H), 7.35-7.23 (m, 5H), 5.34 (d, ***J*** = 6.4 Hz, 1H), 5.24-5.20 (m, 1H), 4.08-3.96 (m, 2H), 3.82 (s, 3H) ^{13}C-NMR (100 MHz, $CDCl_3$) δ 172.5, 170.6, 140.3, 136.6, 132.6, 131.2 (q, ***J*** = 32 Hz, 1C), 129.3, 129.1 (2C), 128.3, 127.4 (2C), 125.9 (splitted, 1C), 124.4 (splitted, 1C), 124.1 (q, ***J*** = 271 Hz, 1C), 86.1, 57.5, 53.0, 40.5 IR λ 1744, 1621 cm^{-1} HRMS (ES) calcd [M+Na] for $C_{19}H_{16}F_3NNaO_2S$ 402.0752, obsd 402.0753

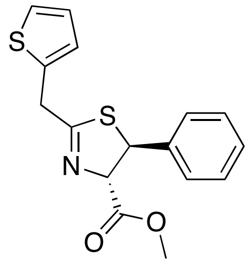

(±)-(4S, 5S)-5-Phenyl-2-thiophen-2-ylmethyl-4,5-dihydro-thiazole-4-carboxylic acid methyl ester ((±)-10d): Following the general procedure starting from 170 mg of (±)-**9e** using 0.6 eq of Lawesson's reagent gave 125 mg of (±)-**10d** as a colorless oil (74% yield) ^{1}H-NMR (400 MHz, $CDCl_3$) δ 7.31-7.19 (m, 6H), 7.00-6.97 (m, 1H), 6.97-6.93 (m, 1H), 5.33 (d, ***J*** = 6.8 Hz, 1H), 5.19-5.15 (m, 1H), 4.19-4.08 (m, 2H), 3.78 (s, 3H) ^{13}C-NMR (100 MHz, $CDCl_3$) δ 172.8, 170.6, 140.2, 137.1, 129.0 (2C), 128.2, 127.5 (2C), 127.2 (2C), 125.4, 86.1, 57.3, 52.9, 35.0 IR λ 1744, 1266 cm^{-1} HRMS (ES) calcd [M+Na] for $C_{16}H_{15}NNaO_2S_2$ 340.0442, obsd 340.0446

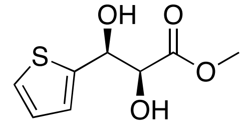

(±)-(2S, 3S)-2,3-Dihydroxy-3-thiophen-2-yl-propionic acid methyl ester ((±)-12d): Following the general procedure starting from 800 mg of (±)-**11d** gave 700 mg of (±)-**12d** as a colorless non crystalline solid (73% yield) ^{1}H-NMR

(400 MHz, $CDCl_3$) δ 7.31-7.27 (m, 1H), 7.11-7.06 (m, 1H), 7.01-6.96 (m, 1H), 5.27-5.24 (m,1H), 4.43 (d, ***J*** = 2.8 Hz, 1H), 3.84 (s, 3H) ^{13}C-NMR (100 MHz, $CDCl_3$) δ 172.8, 143.3, 126.8, 125.9, 125.4, 74.4, 71.0, 53.2 IR λ 3538, 1742 cm^{-1} HRMS (ES) calcd [M+Na] for $C_8H_{10}NaO_4S$ 225.0197, obsd 225.0205

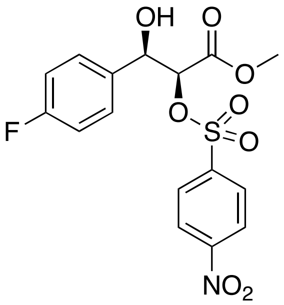

(±)-(2S, 3R)-3-(4-Fluoro-phenyl)-3-hydroxy-2-(4-nitro-benzenesulfonyloxy)-propionic acid methyl ester ((±)-**13a)**: Following the general procedure starting from 400 mg of (±)**-12a** gave 470 mg of (±)-**13a** as a colorless non crystalline solid (63% yield) ^{1}H-NMR (400 MHz, $CDCl_3$) δ 8.31-8.26 (m, 2H), 7.93-7.88 (m, 2H), 7.29-7.21 (m, 2H), 7.00-6.91 (m, 2H), 5.22 (d, ***J*** = 4.0 Hz, 1H), 5.04 (d, ***J*** = 4.0 Hz, 1H), 3.70 (s, 3H) ^{13}C-NMR (100 MHz, $CDCl_3$:d_6-DMSO 1:1) δ 167.1, 162.5 (d, ***J*** = 246 Hz, 1C), 150.6, 141.4, 134.1 (d, ***J*** = 3 Hz, 1C), 129.1 (2C), 127.9 (d, ***J*** = 8 Hz, 1C), 124.1 (2C), 115.2 (d, ***J*** = 22 Hz, 1C), 82.7, 72.3, 52.9 IR λ 3523, 1748, 1538 cm^{-1} HRMS (ES) calcd [M+Na] for $C_{16}H_{14}FNNaO_8S$ 422.0322, obsd 422.0321

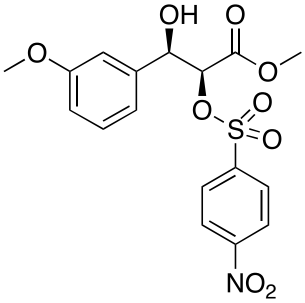

(±)-(2S, 3R)-3-Hydroxy-3-(3-methoxy-phenyl)-2-(4-nitro-benzenesulfonyloxy)-propionic acid methyl ester ((±)-**13b)**: Following the general procedure starting from 500 mg of (±)**-12b** gave 634 mg of (±)-**13b** as a colorless non crystalline solid (70% yield) ^{1}H-NMR (400 MHz, $CDCl_3$) δ 8.20-8.14 (m, 2H), 7.78-7.72 (m, 2H), 7.18-7.10 (m, 1H), 6.81-6.77 (m, 1H), 6.75-6.70 (m, 1H), 6.66-6.64 (m, 1H), 5.23 (d, ***J*** = 3.2 Hz, 1H), 5.02 (d, ***J*** = 3.2 Hz, 1H), 3.81 (s, 3H), 3.69 (s, 3H), 2.47 (brs, 1H) ^{13}C-NMR (100 MHz, d_6-DMSO) δ 167.1, 158.5, 149.9, 140.8, 140.0, 128.8 (split, 3C), 124.2 (2C), 118.3, 113.0, 110.9, 82.7, 71.6, 54.6, 52.7 IR λ 3551, 1777, 1740, 1531 cm^{-1} HRMS (ES) calcd [M+Na] for $C_{17}H_{17}NNaO_9S$ 434.0522, obsd 434.0524

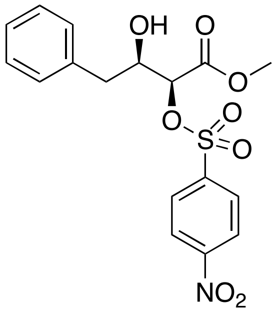

(±)-(2S, 3R)-3-Hydroxy-2-(4-nitro-benzenesulfonyloxy)-4-phenyl-butyric acid methyl ester ((±)-13c): Following the general procedure starting from 800 mg of (±)-**12c** gave 930 mg of (±)-**13c** as a colorless non crystalline solid (62% yield) ^{1}H-NMR (400 MHz, $CDCl_3$) δ 8.44-8.38 (m, 2H), 8.23-8.17 (m, 2H), 7.38-7.21 (m, 5H), 5.07 (d, ***J*** = 2.8 Hz, 1H), 4.42-4.36 (m, 1H), 3.70 (s, 3H), 3.00-2.87 (m, 2H), 2.22 (brs, 1H) ^{13}C-NMR (100 MHz, $CDCl_3$) δ 167.4, 150.9, 142.0, 136.1, 129.7 (2C), 129.4 (2C), 129.0 (2C), 127.3, 124.4 (2C), 80.0, 72.7, 53.1, 39.5 IR λ 3510, 1761, 1535 cm^{-1} HRMS (ES) calcd [M+Na] for $C_{17}H_{17}NNaO_8S$ 418.0573, obsd 418.0570

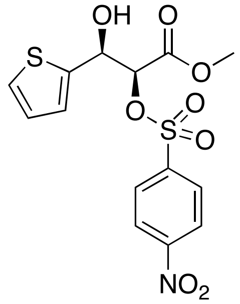

(±)-(2S, 3S)-3-Hydroxy-2-(4-nitro-benzenesulfonyloxy)-3-thiophen-2-yl-propionic acid methyl ester ((±)-13d): Following the general procedure starting from 105 mg of (±)-**12d** gave 109 mg of (±)-**13d** as a colorless non crystalline solid (54% yield) ^{1}H-NMR (400 MHz, $CDCl_3$) δ 8.32-8.27 (m, 2H), 8.00-7.94 (m, 2H), 7.25-7.22 (m, 1H), 6.97-6.94 (m, 1H), 6.92-6.88 (m, 1H), 5.49-5.45 (m, 1H), 5.13 (d, ***J*** = 3.6 Hz, 1H), 3.74 (s, 3H) ^{13}C-NMR (100 MHz, $CDCl_3$) δ 166.7, 150.9, 141.7, 140.8, 129.4 (2C), 127.2, 126.3, 125.4, 124.3 (2C), 81.7, 70.4, 53.4 IR λ 3521, 1739, 1535 cm^{-1} HRMS (ES) calcd [M+Na] for $C_{14}H_{13}NNaO_8S_2$ 409.9980, obsd 409.9980

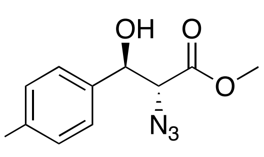

(±)-(2R, 3R)-2-Azido-3-(4-fluoro-phenyl)-3-hydroxy-propionic acid methyl ester ((±)-14a): Following the general procedure starting from 300 mg of (±)-**13a** gave 117 mg of (±)-**14a** as a colorless non crystalline solid after HPLC purification (65% yield) ^{1}H-NMR (400 MHz, $CDCl_3$) δ 7.40-7.33 (m, 2H), 7.11-7.02 (m, 2H), 5.00 (d, ***J*** = 7.2 Hz, 1H), 4.10 (d, ***J*** = 7.2 Hz, 1H), 3.79 (s, 3H), 3.01 (brs, 1H) ^{13}C-NMR (100 MHz, $CDCl_3$) δ 169.4, 163.0 (d, ***J*** = 246 Hz, 1C), 134.8 (d, ***J***

= 3 Hz, 1C), 128.6 (d, ***J*** = 8 Hz, 2C), 115.7 (d, ***J*** = 22 Hz, 2C), 73.5, 67.0, 53.0 IR λ 2116, 1746 cm^{-1} HRMS (ES) calcd [M+Na] for $C_{10}H_{10}FN_3NaO_3$ 262.0604, obsd 262.0605

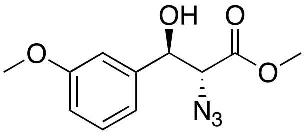

(±)-(2R, 3R)-2-Azido-3-hydroxy-3-(3-methoxy-phenyl)-propionic acid methyl ester ((±)-14b): Following the general procedure starting from 620 mg of (±)**-13b** gave 310 mg of (±)-**14b** as a colorless non crystalline solid (82% yield) ^{1}H-NMR (400 MHz, $CDCl_3$) δ 7.32-7.25 (m, 1H), 6.97-6.91 (m, 2H), 6.90-6.85 (m, 1H), 4.97 (d, ***J*** = 7.2 Hz, 1H), 4.09 (d, ***J*** = 7.2 Hz, 1H), 3.80 (s, 3H), 3.78 (s, 3H), 3.01 (brs, 1H) ^{13}C-NMR (100 MHz, $CDCl_3$) δ 169.5, 159.9, 140.7, 129.8, 118.9, 114.4, 112.2, 74.1, 66.8, 55.4, 52.9 IR λ 3488, 2115, 1745, 1602 cm^{-1} HRMS (ES) calcd [M+Na] for $C_{11}H_{13}N_3NaO_4$ 274.0804, obsd 274.0805

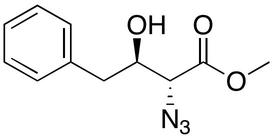

(±)-(2R, 3R)-2-Azido-3-hydroxy-4-phenyl-butyric acid methyl ester ((±)-14c): Following the general procedure starting from 850 mg of (±)**-13c** gave 400 mg of (±)-**14c** as a pale yellow oil (79% yield) ^{1}H-NMR (400 MHz, $CDCl_3$) δ 7.38-7.32 (m, 2H), 7.31-7.24 (m, 3H), 4.25-4.18 (m, 1H), 4.00 (d, ***J*** = 5.6 Hz, 1H), 3.81 (s, 3H), 3.01-2.81 (m, 2H), 2.55 (brs, 1H) ^{13}C-NMR (100 MHz, $CDCl_3$) δ 169.3, 136.9, 129.6 (2C), 128.7 (2C), 126.9, 72.8, 65.4, 52.8, 39.4 IR λ 3568, 2113, 1744 cm^{-1} HRMS (ES) calcd [M+Na] for $C_{11}H_{13}N_3NaO_3$ 258.0855, obsd 258.0858

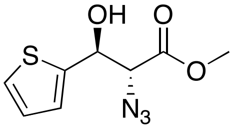

(±)-(2R, 3S)-2-Azido-3-hydroxy-3-thiophen-2-yl-propionic acid methyl ester (14d): Following the general procedure starting from 650 mg of (±)**-13d** gave 297 mg of (±)-**14d** as a colorless oil (78% yield) ^{1}H-NMR (400 MHz, $CDCl_3$) δ 7.35-7.31 (m, 1H), 7.10-7.07 (m, 1H), 7.03-6.99 (m, 1H), 5.31 (d, ***J*** = 6.8 Hz, 1H), 4.24 (d, ***J*** = 6.8 Hz, 1H), 3.81 (s, 3H), 3.02 (brs, 1H) ^{13}C-NMR (100 MHz, $CDCl_3$) δ 169.0, 142.0, 127.0, 126.3, 126.1, 70.5, 67.0, 53.1 IR λ 3482, 2117, 1744 cm^{-1} HRMS (ES) calcd [M+Na] for $C_8H_9N_3NaO_3S$ 250.0262, obsd 250.0263

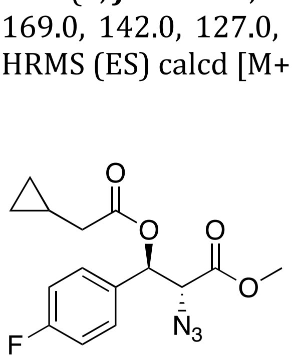

(±)-(2R, 3R)-2-Azido-3-(2-cyclopropyl-acetoxy)-3-(4-fluoro-phenyl)-propionic acid methyl ester ((±)-15a): Following the general procedure starting from 150 mg of (±)**-14a** gave 173 mg of (±)-**15a** as a colorless oil (85%

yield) ^{1}H-NMR (400 MHz, $CDCl_3$) δ 7.41-7.33 (m, 2H), 7.10-7.02 (m, 2H), 6.13 (d, ***J*** = 6.4 Hz, 1H), 4.36 (d, ***J*** = 6.4 Hz, 1H), 3.77 (s, 3H), 2.27 (d, ***J*** = 7.2 Hz, 1H), 1.09-0.97 (m, 1H), 0.59-0.52 (m, 2H), 0.20-0.13 (m, 2H) ^{13}C-NMR (100 MHz, $CDCl_3$) δ 171.5, 167.7, 163.1 (d, ***J*** = 247 Hz, 1C), 131.3 (d, ***J*** = 3 Hz, 1C), 129.3 (d, ***J*** = 8 Hz, 2C), 115.8 (d, ***J*** = 22 Hz, 2C), 73.8, 65.4, 53.0, 39.4, 6.9, 4.6, 4.5 IR λ 2116, 1750 cm^{-1} HRMS (ES) calcd [M+Na] for $C_{15}H_{16}FN_3NaO_4$ 344.1023, obsd 344.1021

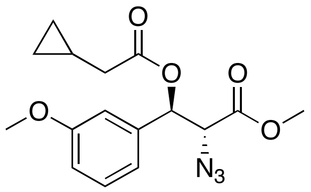

(±)-(2R, 3R)-2-Azido-3-(2-cyclopropyl-acetoxy)-3-(3-methoxy-phenyl)-propionic acid methyl ester ((±)-**15b)**: Following the general procedure starting from 150 mg of (±)**-14b** gave 188 mg of (±)**15b** as a colorless oil (94% yield) ^{1}H-NMR (400 MHz, $CDCl_3$) δ 7.25-7.21 (m, 1H), 6.94-6.81 (m, 3H), 6.08 (d, ***J*** = 6.4 Hz, 1H), 4.28 (d, ***J*** = 6.4 Hz, 1H), 3.76 (s, 3H), 3.73 (s, 3H), 2.24 (d, ***J*** = 7.2 Hz, 1H), 1.07-0.95 (m, 1H), 0.55-0.48 (m, 2H), 0.16-0.10 (m, 2H) ^{13}C-NMR (100 MHz, $CDCl_3$) δ 171.5, 167.8, 159.8, 136.9, 129.8, 119.4, 114.5, 113.0, 74.4, 65.4, 55.3, 52.9, 39.4, 6.9, 4.5 (split, 2C) IR λ 2115, 1750 cm^{-1} HRMS (ES) calcd [M+Na] for $C_{16}H_{19}N_3NaO_5$ 356.1222, obsd 356.1221

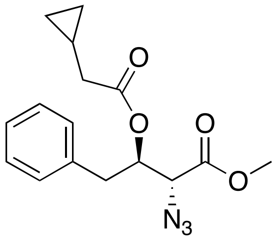

(±)-(2R, 3R)-2-Azido-3-(2-cyclopropyl-acetoxy)-4-phenyl-butyric acid methyl ester ((±)-**15c)**: Following the general procedure starting from 150 mg of (±)**-14c** gave 153 mg of (±)-**15c** as a colorless oil (75% yield) ^{1}H-NMR (400 MHz, $CDCl_3$) δ 7.32-7.19 (m, 5H), 5.53-5.46 (m, 1H), 4.21 (d, ***J*** = 4.4 Hz, 1H), 3.71 (s, 3H), 3.05-2.92 (m, 2H), 2.18 (d, ***J*** = 7.2 Hz, 1H), 1.02-0.90 (m, 1H), 0.54-0.46 (m, 2H), 0.15-0.06 (m, 2H) ^{13}C-NMR (100 MHz, $CDCl_3$) δ 172.1, 167.8, 135.8, 129.6 (2C), 128.5 (2C), 127.0, 73.9, 63.3, 52.8, 39.4, 36.1, 6.7, 4.4 (2C) IR λ 2114, 1747 cm^{-1} HRMS (ES) calcd [M+Na] for $C_{16}H_{19}N_3NaO_4$ 340.1273, obsd 340.1271

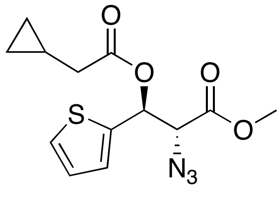

(±)-(2R, 3S)-2-Azido-3-(2-cyclopropyl-acetoxy)-3-thiophen-2-yl-propionic acid methyl ester ((±)-**15d)**: Following the general procedure starting from 130

mg of (±)-**14d** gave 142 mg of (±)-**15d** as a colorless oil (81% yield) ^{1}H-NMR (400 MHz, $CDCl_3$) δ 7.36-7.32 (m, 1H), 7.15-7.11 (m, 1H), 7.01-6.97 (m, 1H), 6.48 (d, ***J*** = 6.0 Hz, 1H), 4.48 (d, ***J*** = 6.0 Hz, 1H), 3.78 (s, 3H), 2.27 (d, ***J*** = 7.2 Hz, 1H), 1.09-0.98 (m, 1H), 0.58-0.51 (m, 2H), 0.19-0.13 (m, 2H) ^{13}C-NMR (100 MHz, $CDCl_3$) δ 171.5, 167.3, 137.1, 127.8, 127.0, 126.8, 70.6, 65.5, 53.1, 39.4, 6.8, 4.5 (2C) IR λ 2117, 1751 cm^{-1} HRMS (ES) calcd [M+Na] for $C_{13}H_{15}N_3NaO_4S$ 332.0681, obsd 332.0681

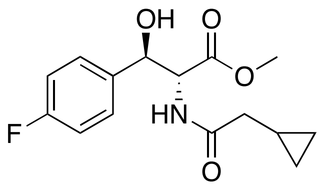

(±)-(2R, 3R)-2-(2-Cyclopropyl-acetylamino)-3-(4-fluoro-phenyl)-3-hydroxy-propionic acid methyl ester ((±)-**16a)**: Following the general procedure starting from 50 mg of (±)-**15a** gave 39 mg of (±)-**16a** as a colorless oil (83% yield) ^{1}H-NMR (400 MHz, $CDCl_3$) δ 7.23-7.16 (m, 2H), 7.04-6.95 (m, 2H), 6.71-6.63 (m, 1H), 5.25 (d, ***J*** = 3.6 Hz, 1H), 5.03-4.98 (m, 1H), 3.75 (s, 3H), 2.23-2.10 (m, 2H), 0.94-0.82 (m, 1H), 0.61-0.51 (m, 2H), 0.18-0.07 (m, 2H) ^{13}C-NMR (100 MHz, $CDCl_3$) δ 174.5, 169.9, 162.3 (d, ***J*** = 245 Hz, 1C), 135.0 (d, ***J*** = 3 Hz, 1C), 127.8 (d, ***J*** = 8 Hz, 2C), 115.3 (d, ***J*** = 22Hz, 2C), 74.8, 59.4, 52.9, 41.1, 7.0, 4.7 (split, 2C) IR λ 3418, 1743, 1652, 1511 cm^{-1} HRMS (ES) calcd [M+Na] for $C_{15}H_{18}FNNaO_4$ 318.1118, obsd 318.1119

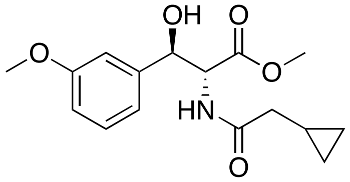

(±)-(2R, 3R)-2-(2-Cyclopropyl-acetylamino)-3-hydroxy-3-(3-methoxy-phenyl)-propionic acid methyl ester ((±)-**16b)**: Following the general procedure starting from 165 mg of (±)-**15b** gave 120 mg of (±)-**16b** as a colorless non crystalline solid (80% yield) ^{1}H-NMR (400 MHz, $CDCl_3$) δ 7.25-7.18 (m, 1H), 6.83-6.74 (m, 3H), 6.69-6.63 (m, 1H), 5.25 (d, ***J*** = 3.6 Hz, 1H), 5.03-4.99 (m, 1H), 3.77 (s, 3H), 3.75 (s, 3H), 2.15 (d, ***J*** = 7.2 Hz, 1H), 0.93-0.82 (m, 1H), 0.59-0.50 (m, 2H), 0.17-0.07 (m, 2H) ^{13}C-NMR (100 MHz, $CDCl_3$) δ 174.3, 170.0, 159.7, 140.9, 129.4, 118.3, 113.6, 111.7, 75.2, 59.2, 55.3, 52.8, 41.1, 7.0, 4.7, 4.6 IR λ 3418, 1744, 1652, 1520 cm^{-1} HRMS (ES) calcd [M+Na] for $C_{16}H_{21}NNaO_5$ 330.1317, obsd 330.1313

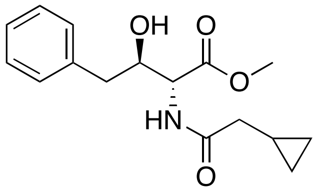

(±)-(2R, 3R)-2-(2-Cyclopropyl-acetylamino)-3-hydroxy-4-phenyl-butyric

acid methyl ester ((±)-16c): Following the general procedure starting from 130 mg of (±)**-15c** gave 92 mg of (±)-**16c** as a colorless oil (77% yield) ^{1}H-NMR (400 MHz, $CDCl_3$) δ 7.36-7.22 (m, 5H), 7.05-6.97 (m, 1H), 4.80-4.75 (m, 1H), 4.35-4.29 (m, 1H), 3.72 (s, 3H), 2.93-2.81 (m, 2H), 2.33-2.19 (m, 2H), 1.10-0.99 (m, 1H), 0.73-0.66 (m, 2H), 0.31-0.25 (m, 2H) ^{13}C-NMR (100 MHz, $CDCl_3$) δ 173.7, 170.5, 137.4, 129.5 (2C), 128.6 (2C), 126.8, 74.2, 57.7, 52.7, 41.2, 40.0, 7.2, 4.8, 4.7 IR λ 3377, 1742, 1650, 1522 cm^{-1} HRMS (ES) calcd [M+Na] for $C_{16}H_{21}NNaO_4$ 314.1368, obsd 314.1374

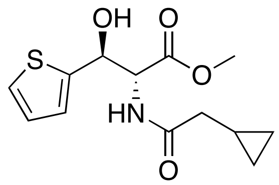

(±)-(2R, 3S)-2-(2-Cyclopropyl-acetylamino)-3-hydroxy-3-thiophen-2-yl-propionic acid methyl ester ((±)-16d): Following the general procedure starting from 70 mg of (±)**-15d** gave 51mg of (±)-**16d** as a colorless oil (78% yield) ^{1}H-NMR (400 MHz, $CDCl_3$) δ 7.25-7.21 (m, 1H), 6.97-6.92 (m, 1H), 6.87-6.79 (m, 2H), 5.56 (d, ***J*** = 3.6 Hz, 1H), 5.12-5.06 (m, 1H), 3.78 (s, 3H), 2.27-2.15 (m, 2H), 1.00-0.88 (m, 1H), 0.63-0.55 (m, 2H), 0.21-0.13 (m, 2H) ^{13}C-NMR (100 MHz, $CDCl_3$) δ 174.9, 169.5, 142.7, 126.8, 125.3, 124.3, 72.3, 59.2, 53.0, 41.1, 7.0, 4.7 (2C) IR λ 3386, 1739, 1652, 1525 cm^{-1} HRMS (ES) calcd [M+Na] for $C_{13}H_{17}NNaO_4S$ 306.0776, obsd 306.0766

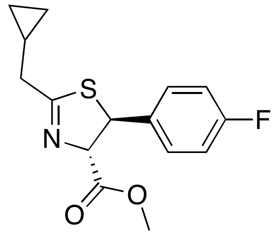

(±)-(4S, 5S)-2-Cyclopropylmethyl-5-(4-fluoro-phenyl)-4,5-dihydro-thiazole-4-carboxylic acid methyl ester ((±)-17a): Following the general procedure starting from 35 mg of (±)**-16a** using 0.6 eq of Lawesson's reagent gave 25 mg of (±)-**17a** as a colorless oil (71% yield) ^{1}H-NMR (400 MHz, $CDCl_3$) δ 7.37-7.30 (m, 2H), 7.06-6.98 (m, 2H), 5.31 (d, ***J*** = 6.8 Hz, 1H), 5.08 (d, ***J*** = 6.8 Hz, 1H), 3.79 (s, 3H), 2.59-2.47 (m, 2H), 1.10-0.98 (m, 1H), 0.65-0.57 (m, 2H), 0.30-0.23 (m, 2H) ^{13}C-NMR (100 MHz, $CDCl_3$) δ 174.9, 170.8, 162.5 (d, ***J*** = 246 Hz, 1C), 136.4 (d, ***J*** = 3 Hz, 1C), 129.3 (d, ***J*** = 8 Hz, 2C), 116.0 (d, ***J*** = 22 Hz, 2C), 86.0, 55.9, 53.0, 39.4, 9.4, 5.2, 5.1 IR λ 1744, 1619, 1510, 1266 cm^{-1} HRMS (ES) calcd [M+Na] for $C_{15}H_{16}FNNaO_2S$ 316.0783, obsd 316.0782

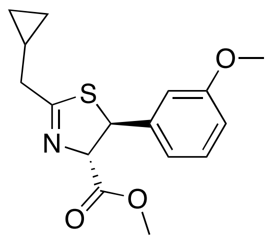

(±)-(4S, 5S)-2-Cyclopropylmethyl-5-(3-methoxy-phenyl)-4,5-dihydro-thiazole-4-carboxylic acid methyl ester ((±)-17b): Following the general procedure starting from 120 mg of (±)**-16b** using 0.6 eq of Lawesson's reagent gave 95 mg of (±)-**17b** as a colorless oil (80% yield) ^{1}H-NMR (400 MHz, $CDCl_3$) δ 7.27-7.22 (m, 1H), 6.96-6.92 (m, 1H), 6.91-6.89 (m, 1H), 6.84-679 (m, 1H), 5.29 (d, ***J*** = 6.4 Hz, 1H), 5.14 (d, ***J*** = 6.4 Hz, 1H), 3.81-3.77 (m, 6H), 2.53 (d, ***J*** = 7.2 Hz, 2H) 1.10-1.00 (m, 1H), 0.64-0.57 (m, 2H), 0.30-0.24 (m, 2H) ^{13}C-NMR (100 MHz, $CDCl_3$) δ 174.8, 170.9, 160.1, 142.2, 130.1, 119.8, 113.6, 113.2, 85.9, 56.5, 55.4, 52.9, 39.4, 9.4, 5.2, 5.1 IR λ 1744, 1600, 1266, 1162 cm^{-1} HRMS (ES) calcd [M+Na] for $C_{16}H_{19}NNaO_3S$ 328.0983, obsd 328.0980

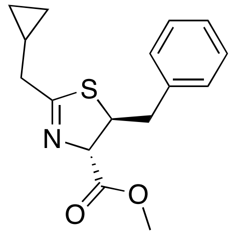

(±)-(4S, 5S)-5-Benzyl-2-cyclopropylmethyl-4,5-dihydro-thiazole-4-carboxylic acid methyl ester ((±)-17c): Following the general procedure starting from 80 mg of (±)**-16c** using 0.6 eq of Lawesson's reagent gave 19 mg of (±)-**17c** as a colorless oil (24% yield) ^{1}H-NMR (400 MHz, $CDCl_3$) δ 7.36-7.30 (m, 2H), 7.29-7.21 (m, 3H), 4.88 (d, ***J*** = 6.0 Hz, 1H), 4.50-4.43 (m, 1H), 3.76 (s, 3H), 3.16-2.93 (m, 2H), 2.47 (d, ***J*** = 7.2 Hz, 1H), 1.05-0.93 (m, 1H), 0.62-0.55 (m, 2H), 0.27-0.21 (m, 2H) ^{13}C-NMR (100 MHz, $CDCl_3$) δ 175.3, 171.1, 138.3, 129.1 (2C), 128.7 (2C), 127.1, 81.4, 55.3, 52.8, 42.3, 39.7, 9.3, 5.1 (split, 2C) IR λ 1744, 1618 cm^{-1} HRMS (ES) calcd [M+Na] for $C_{16}H_{19}NNaO_2S$ 312.1034, obsd 312.1031

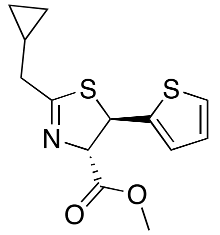

(±)-(4S, 5R)-2-Cyclopropylmethyl-5-thiophen-2-yl-4,5-dihydro-thiazole-4-carboxylic acid methyl ester ((±)-17d): Following the general procedure starting from 85 mg of (±)**-16d** using 0.6 eq of Lawesson's reagent gave 55 mg of (±)-**17d** as a colorless oil (65% yield) ^{1}H-NMR (400 MHz, $CDCl_3$) δ 7.25-7.22 (m, 1H), 7.04-7.01 (m, 1H), 6.95-6.90 (m, 1H), 5.64 (d, ***J*** = 7.2 Hz, 1H), 5.17-5.12 (m, 1H), 3.81 (s, 3H), 2.58-2.46 (m, 2H), 1.09-0.97 (m, 1H), 0.64-0.57 (m, 2H), 0.29-

0.23 (m, 2H) ^{13}C-NMR (100 MHz, $CDCl_3$) δ 174.6, 170.5, 143.9, 127.2, 125.9, 125.7, 85.9, 53.1, 52.1, 39.5, 9.3, 5.2 (split, 2C) IR λ 1744, 1618, 1435, 1214 cm^{-1} HRMS (ES) calcd [M+Na] for $C_{13}H_{15}NNaO_2S_2$ 304.0442, obsd 304.0443

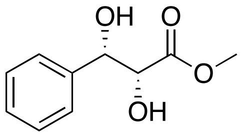

(2R, 3S)-2,3-Dihydroxy-3-phenyl-propionic acid methyl ester ((+)-2): Following the general procedure using AD-mix-α starting from 500 mg methyl cinnamate gave 514 mg of (+)-**2** as a colorless non crystalline solid (85% yield) $[\alpha]_D$ (c=4.0, $CHCl_3$) +10, spectral data agreed with published results.[7]

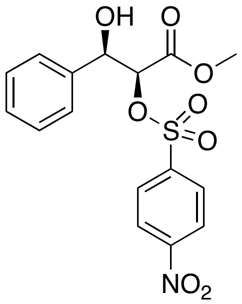

(2S, 3R)-3-Hydroxy-2-(4-nitro-benzenesulfonyloxy)-3-phenyl-propionic acid methyl ester ((-)-3): Following the general procedure starting from 242 mg (-)-**2** gave 356 mg of (-)-**3** as a colorless non crystalline solid (76% yield) $[\alpha]_D$ (c=4.0, $CHCl_3$) -52, spectral data agreed with published results.[8]

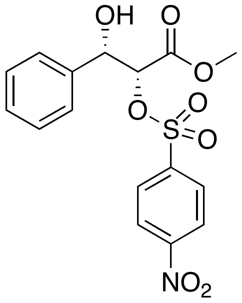

(2R, 3S)-3-Hydroxy-2-(4-nitro-benzenesulfonyloxy)-3-phenyl-propionic acid methyl ester ((+)-3): Following the general procedure starting from 425 mg (+)-**2** gave 650 mg of (+)-**3** as a colorless non crystalline solid (79% yield) $[\alpha]_D$ (c=4.0, $CHCl_3$) +52, spectral data agreed with published results.[8]

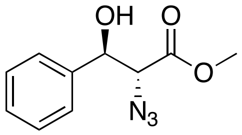

(2R, 3R)-2-Azido-3-phenyl-3-hydroxy-propionic acid methyl ester ((+)-4): Following the general procedure starting from 380 mg (-)-**3** gave 155mg of (+)-**4**

as a colorless non crystalline solid (70% yield) [α]D (c=5.0, $CHCl_3$) +4, spectral data agreed with (±)-**4**

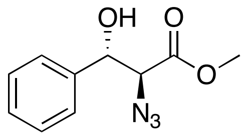

(2S, 3S)-2-Azido-3-phenyl-3-hydroxy-propionic acid methyl ester ((-)-4): Following the general procedure starting from 650 mg (+)-**3** gave 273 mg of (-)-**4** as a colorless non crystalline solid (73% yield) [α]D (c=5.0, $CHCl_3$) -4, spectral data agreed with (±)-**4**

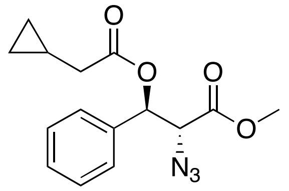

(2R, 3R)-2-Azido-3-(2-cyclopropyl-acetoxy)-3-phenyl-propionic acid methyl ester ((-)-8b): Following the general procedure starting from 150 mg (+)-**4** gave 189 mg of (-)-**8b** as a colorless oil (92% yield) [α]D (c=4.0, $CHCl_3$) -38, spectral data agreed with (±)-**8b**

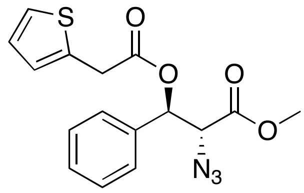

(2R, 3R)-2-Azido-3-phenyl-3-(2-thiophen-2-yl-acetoxy)-propionic acid methyl ester ((-)-8e): Following the general procedure starting from 150 mg (+)-**4** gave 214 mg of (-)-**8e** as a colorless oil (91% yield) [α]D (c=4.0, $CHCl_3$) -29, spectral data agreed with (±)-**8e**

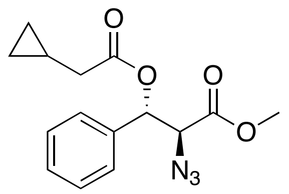

(2S, 3S)-2-Azido-3-(2-cyclopropyl-acetoxy)-3-phenyl-propionic acid methyl ester ((+)-8b): Following the general procedure starting from 132 mg (-)-**4** gave 169 mg of (+)-**8b** as a colorless oil (92% yield) [α]D (c=4.0, $CHCl_3$) +37, spectral data agreed with (±)-**8b**

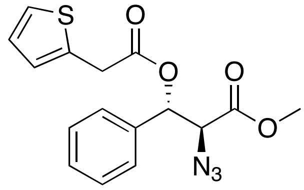

(2S, 3S)-2-Azido-3-phenyl-3-(2-thiophen-2-yl-acetoxy)-propionic acid methyl ester ((+)-**8e)**: Following the general procedure starting from 132 mg (-)-**4** gave 195 mg of (+)-**8e** as a colorless oil (94% yield) [α]$_D$ (c=4.0, $CHCl_3$) +29, spectral data agreed with (±)-**8e**

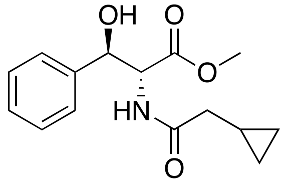

(2R, 3R)-2-(2-Cyclopropyl-acetylamino)-3-hydroxy-3-phenyl-propionic acid methyl ester ((-)-**9b)**: Following the general procedure starting from 179 mg (-)-**8b** gave 117 mg of (-)-**9b** as a colorless non crystalline solid (72% yield) [α]$_D$ (c=4.0, $CHCl_3$) -116, spectral data agreed with (±)-**9b**

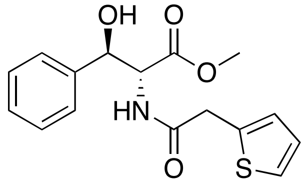

(2R, 3R)-3-Hydroxy-3-phenyl-2-(2-thiophen-2-yl-acetylamino)-propionic acid methyl ester ((-)-**9e)**: Following the general procedure starting from 195 mg (-)-**8e** gave 146 mg of (-)-**9e** as a colorless non crystalline solid (82% yield) [α]$_D$ (c=4.0, $CHCl_3$) -59, spectral data agreed with (±)-**9e**

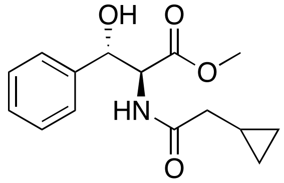

(2S, 3S)-2-(2-Cyclopropyl-acetylamino)-3-hydroxy-3-phenyl-propionic acid methyl ester ((+)-**9b)**: Following the general procedure starting from 165 mg (+)-**8b** gave 110 mg of (+)-**9b** as a colorless non crystalline solid (73% yield) [α]$_D$ (c=4.0, $CHCl_3$) +117, spectral data agreed with (±)-**9b**

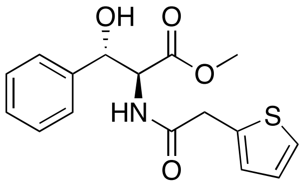

(2s, 3s)-3-Hydroxy-3-phenyl-2-(2-thiophen-2-yl-acetylamino)-propionic acid methyl ester ((+)-9e): Following the general procedure starting from 180 mg (+)-**8e** gave 133 mg of (+)-**9e** as a colorless non crystalline solid (80% yield) $[\alpha]_D$ (c=4.0, $CHCl_3$) +60, spectral data agreed with (±)-**9e**

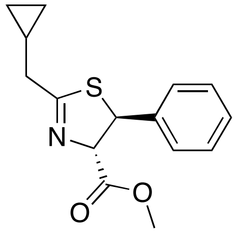

(4S, 5S)-2-Cyclopropylmethyl-5-phenyl-4,5-dihydro-thiazole-4-carboxylic acid methyl ester ((-)-10a): Following the general procedure starting from 70 mg (-)-**9b** using 0.6 eq of Lawesson's reagent gave 51 mg of (-)-**10a** as a colorless oil (74% yield) $[\alpha]_D$ (c=4.0, $CHCl_3$) -159, spectral data agreed with (±)-**10a**

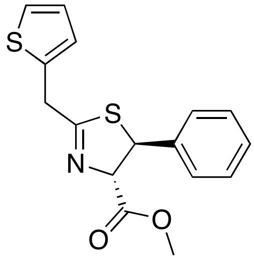

(4S, 5S)-5-Phenyl-2-thiophen-2-ylmethyl-4,5-dihydro-thiazole-4-carboxylic acid methyl ester ((-)-10d): Following the general procedure starting from 127 mg (-)-**9e** using 0.6 eq of Lawesson's reagent gave 90 mg of (-)-**10d** as a colorless oil (71% yield) $[\alpha]_D$ (c=4.0, $CHCl_3$) -142, spectral data agreed with (±)-**10d**

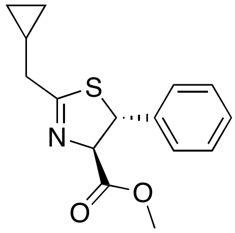

(4R, 5R)-2-Cyclopropylmethyl-5-phenyl-4,5-dihydro-thiazole-4-carboxylic acid methyl ester ((+)-10a): Following the general procedure starting from 105 mg (+)-**9b** using 0.6 eq of Lawesson's reagent gave 84 mg of (+)-**10a** as a colorless oil (80% yield) $[\alpha]_D$ (c=4.0, $CHCl_3$) +124, spectral data agreed with (±)-**10a**

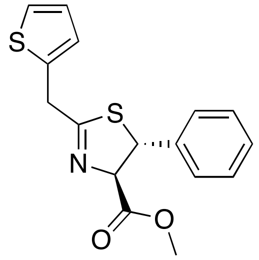

(4R, 5R)-5-Phenyl-2-thiophen-2-ylmethyl-4,5-dihydro-thiazole-4-carboxylic acid methyl ester ((+)-10d): Following the general procedure starting from 105 mg (+)-**9e** using 0.6 eq of Lawesson's reagent gave 75 mg of (+)-**10d** as a colorless oil (72% yield) $[\alpha]_D$ (c=4.0, $CHCl_3$) +120, spectral data agreed with (±)-**10d**

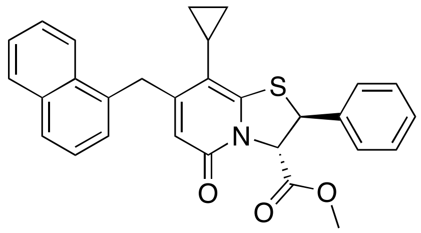

(2S, 3S)-8-Cyclopropyl-7-naphthalen-1-ylmethyl-5-oxo-2-phenyl-2,3-dihydro-5*H*-thiazolo[3,2-*a*]pyridine-3-carboxylic acid methyl ester ((-)-18a): Following the general procedure starting from 43 mg (-)-**10a** gave 61 mg of (-)-**18a** as a pale yellow foam (81% yield) $[\alpha]_D$ (c=2.0, $CHCl_3$) -164, spectral data agreed with published results.[9]

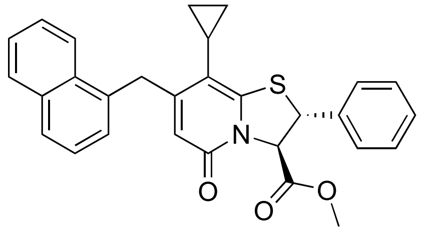

(2R, 3R)-8-Cyclopropyl-7-naphthalen-1-ylmethyl-5-oxo-2-phenyl-2,3-dihydro-5*H*-thiazolo[3,2-*a*]pyridine-3-carboxylic acid methyl ester ((+)-18a): Following the general procedure starting from 80 mg (+)-**10a** gave 108 mg of (+)-**18a** as a pale yellow foam (80% yield) $[\alpha]_D$ (c=2.0, $CHCl_3$) +130, spectral data agreed with published results.[9]

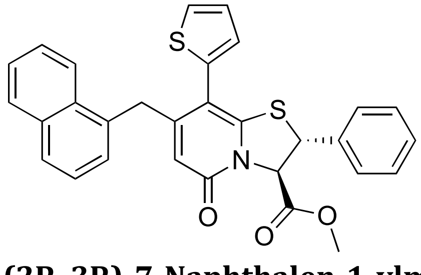

(2R, 3R)-7-Naphthalen-1-ylmethyl-5-oxo-2-phenyl-8-thiophen-2-yl-2,3-dihydro-5*H*-thiazolo[3,2-*a*]pyridine-3-carboxylic acid methyl ester ((+)-18b): Following the general procedure starting from 75 mg (+)-**10d** gave 100

mg of (+)-**18b** as a yellow foam (82% yield) $[\alpha]_D$ (c=2.0, $CHCl_3$) +96, spectral data agreed with (-)-**18b**

^{1}H- and ^{13}C-NMR of compound (±)-**4** in $CDCl_3$

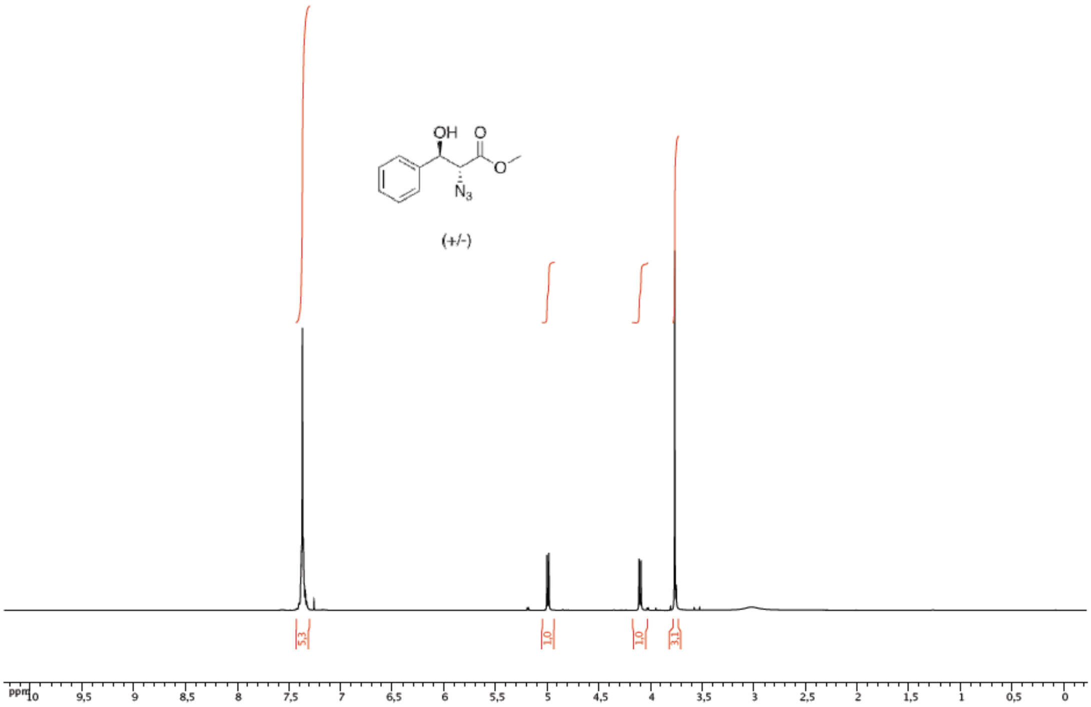

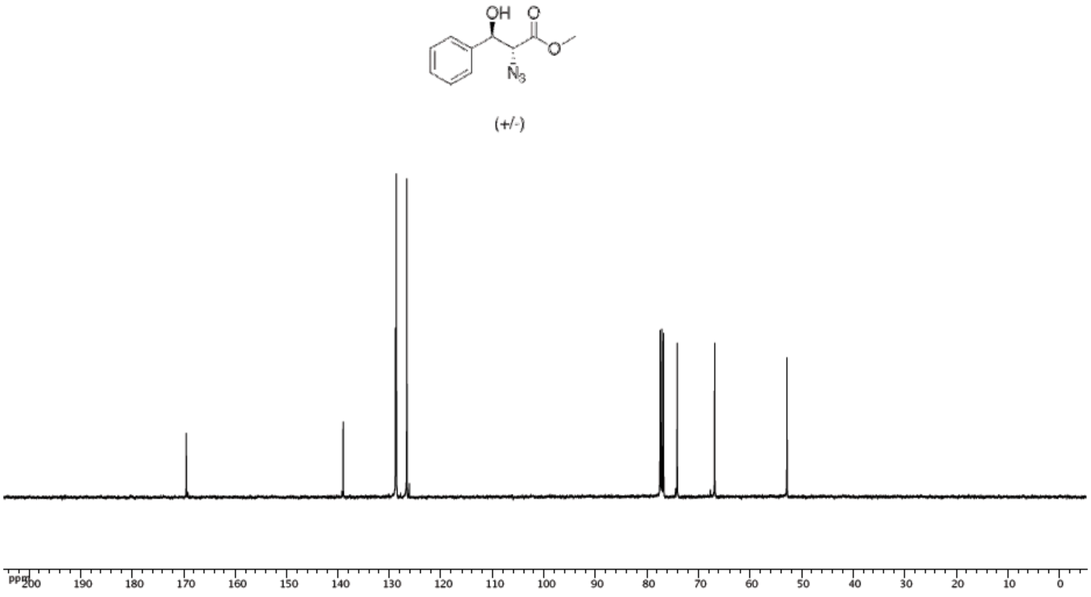

^{1}H- and ^{13}C-NMR of compound (±)-**5** in $CDCl_3$

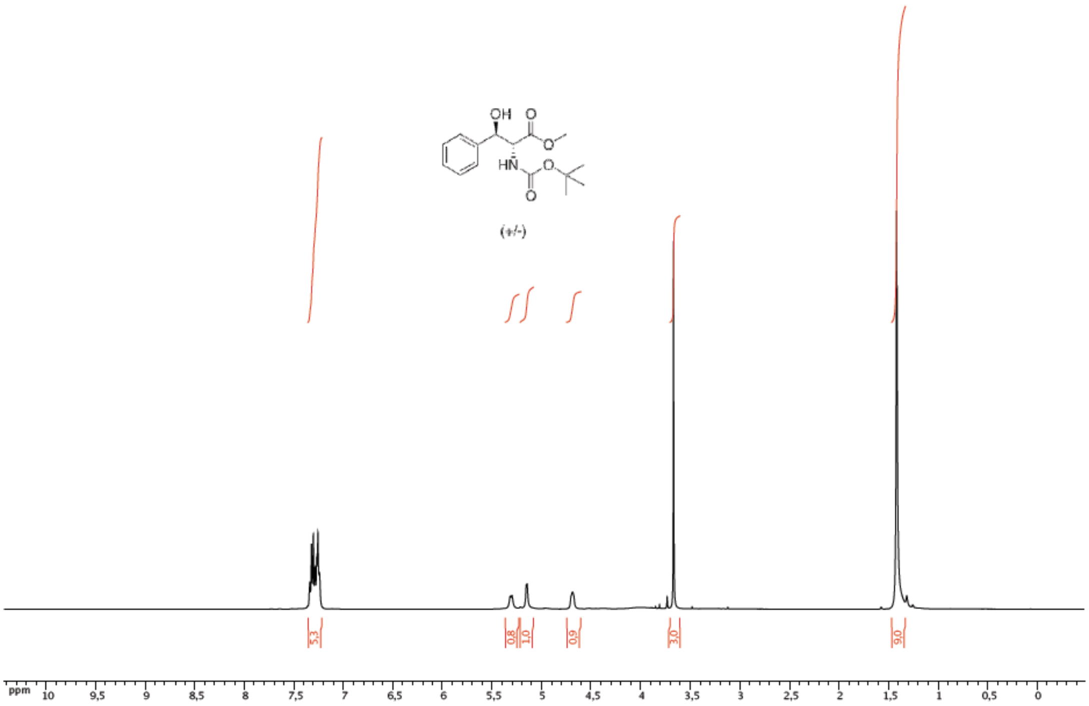

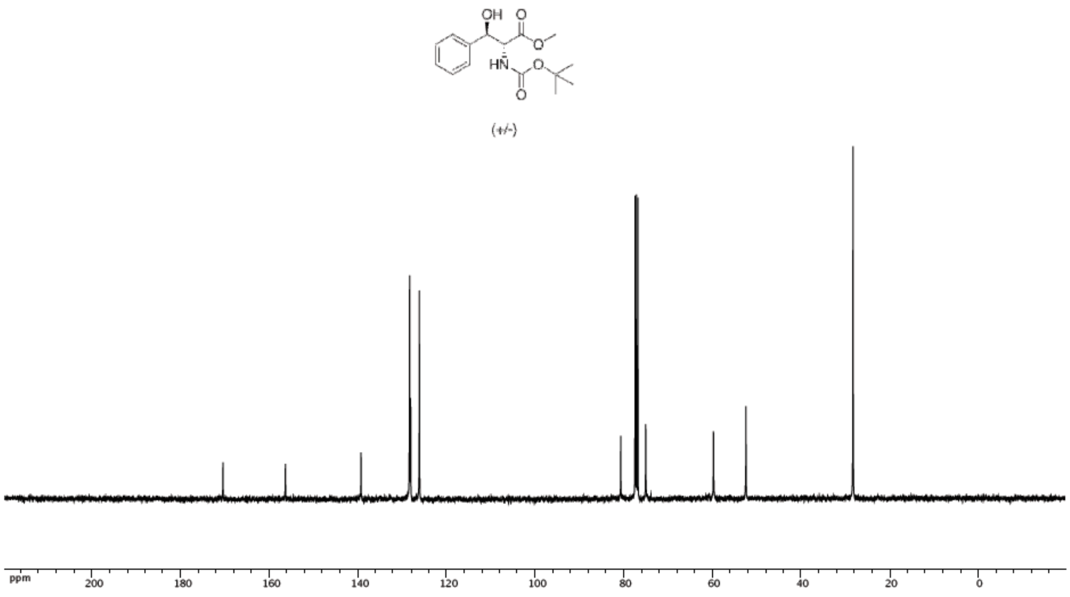

^{1}H- and ^{13}C-NMR of compound (±)-**6** in $CDCl_3$

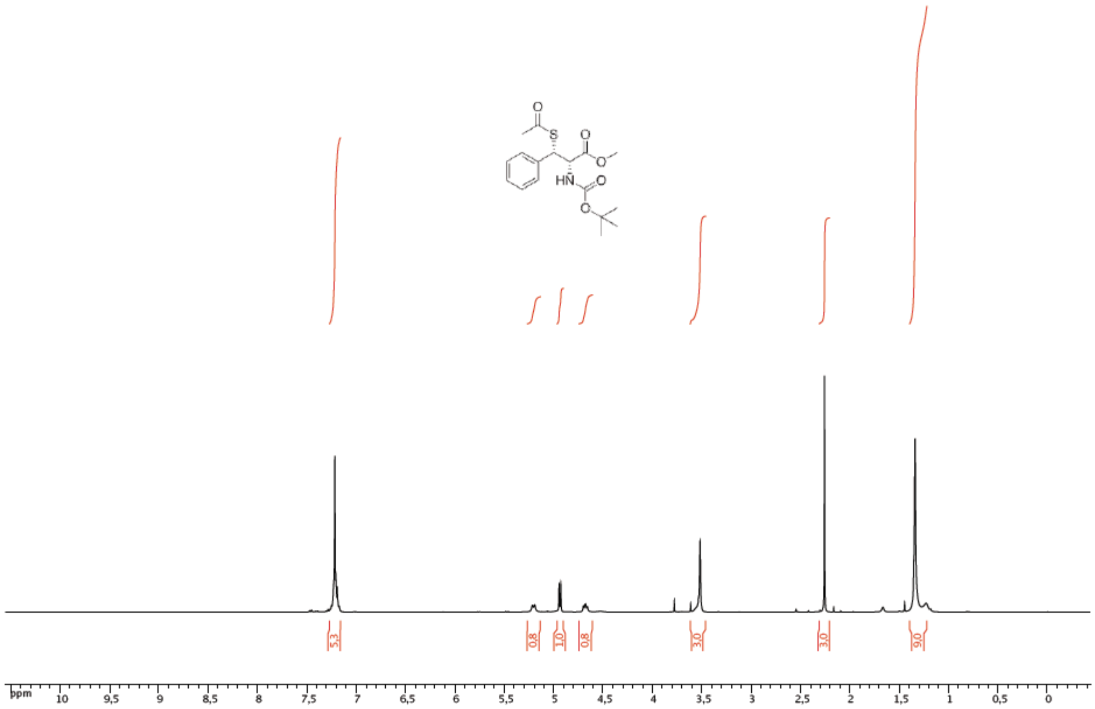

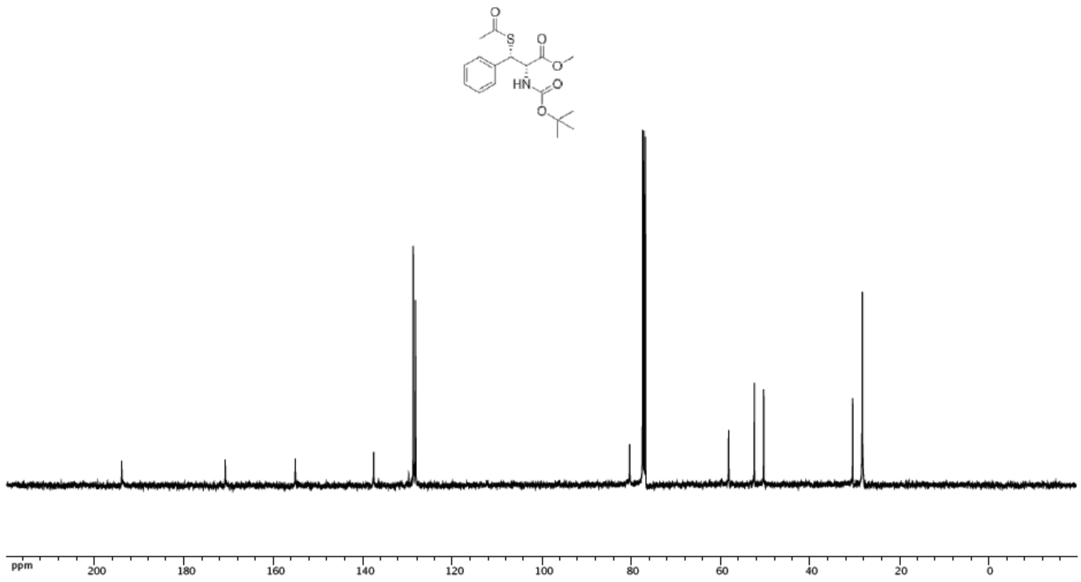

^{1}H- and ^{13}C-NMR of compound (±)-**8a** in $CDCl_3$

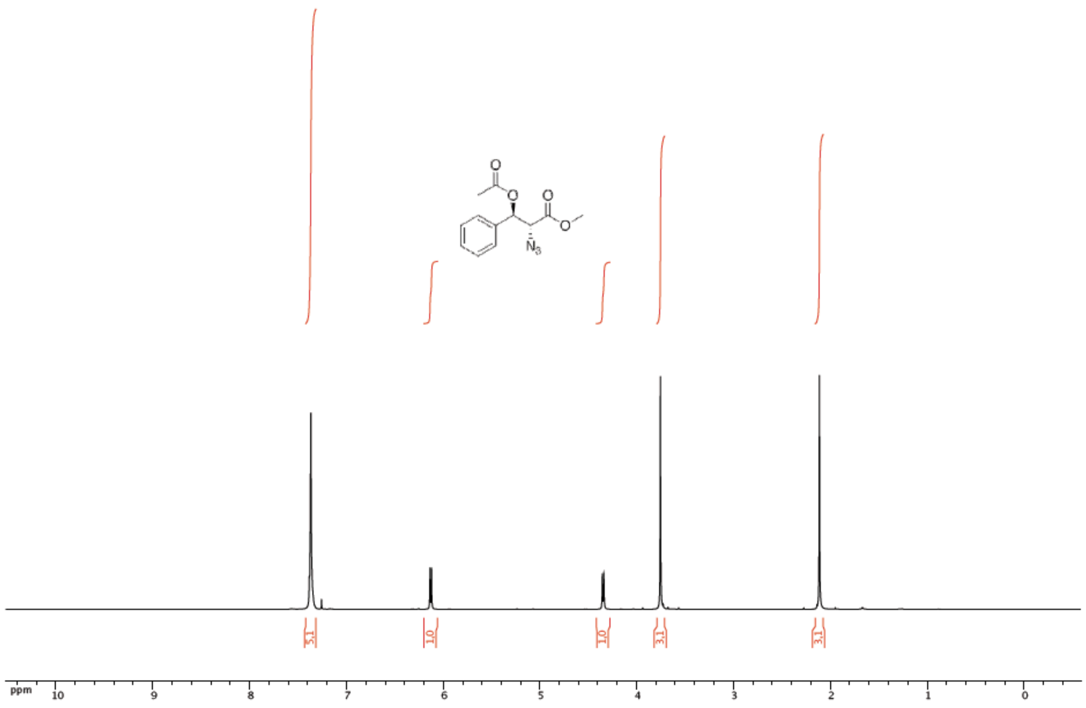

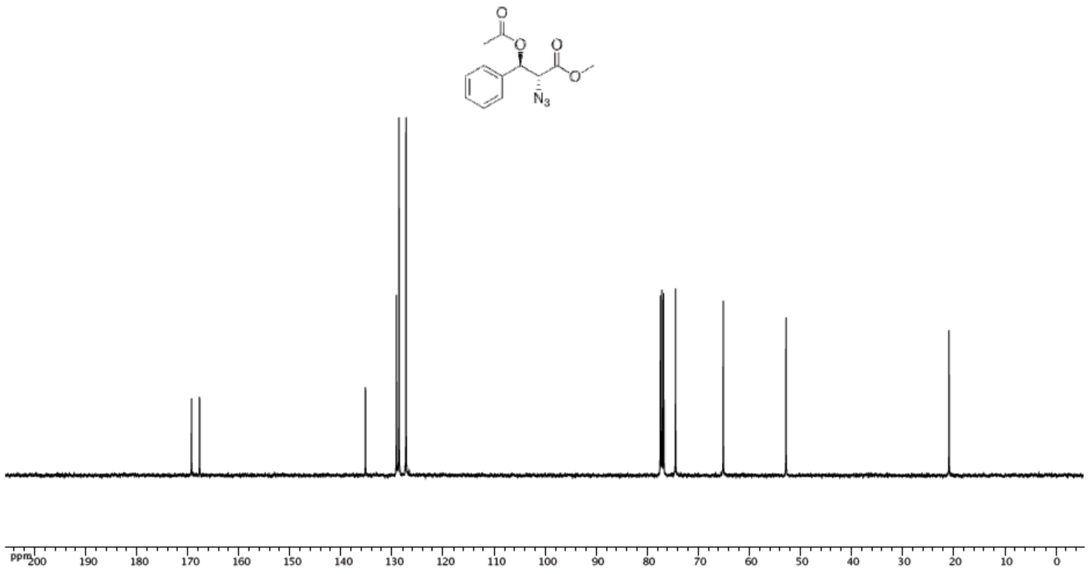

^{1}H- and ^{13}C-NMR of compound (±)-**8b** in $CDCl_3$

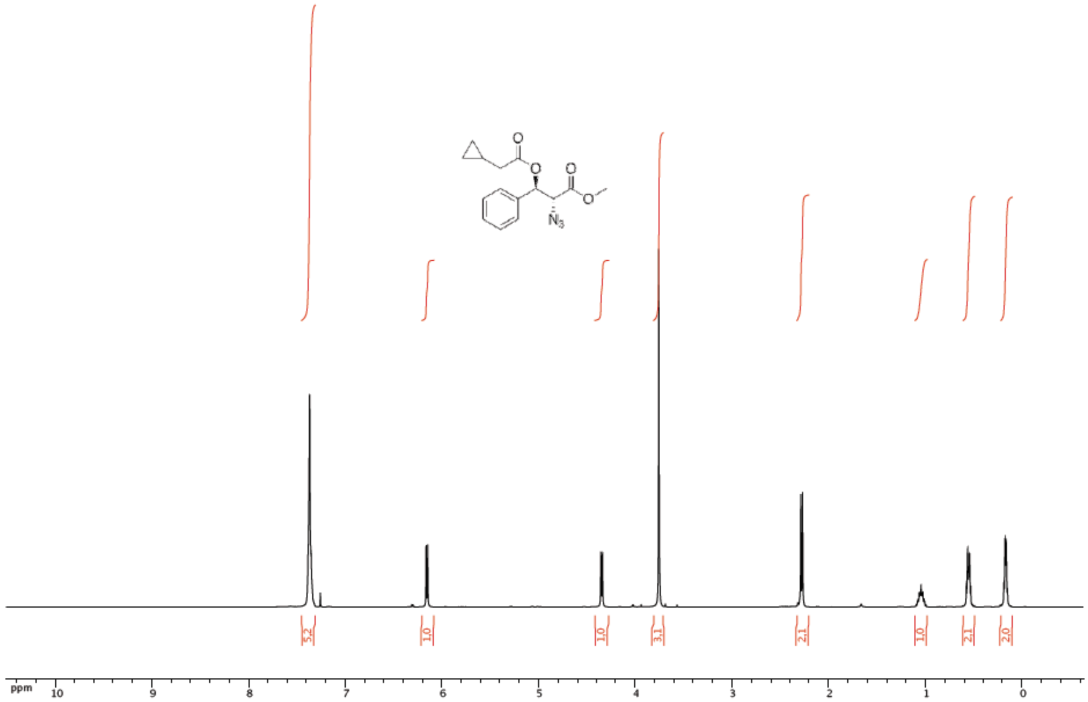

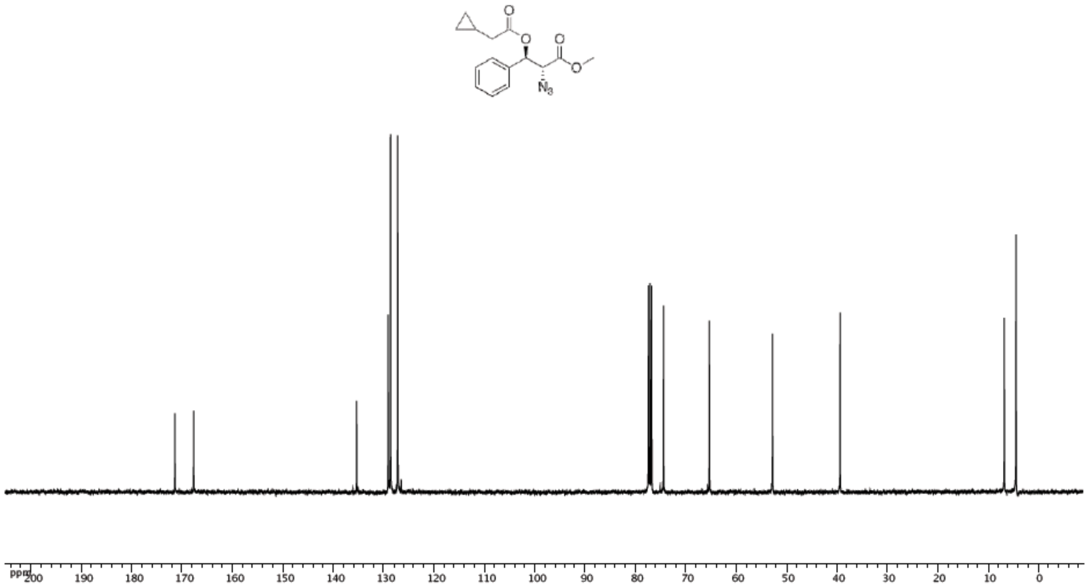

^{1}H- and ^{13}C-NMR of compound (±)-**8c** in $CDCl_3$

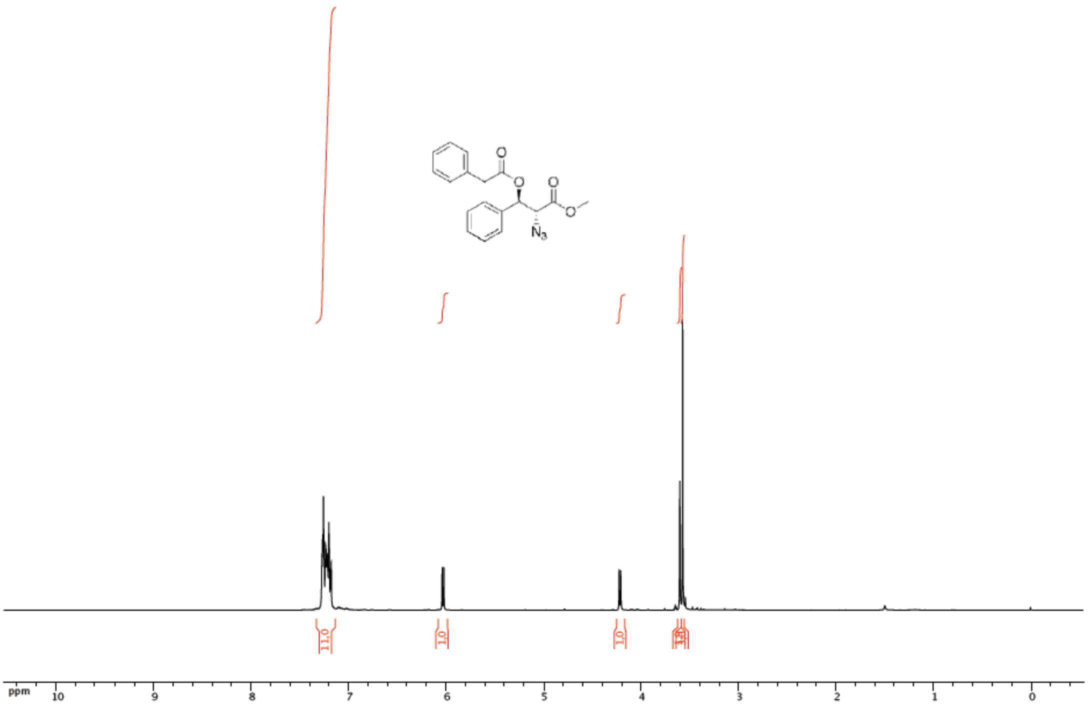

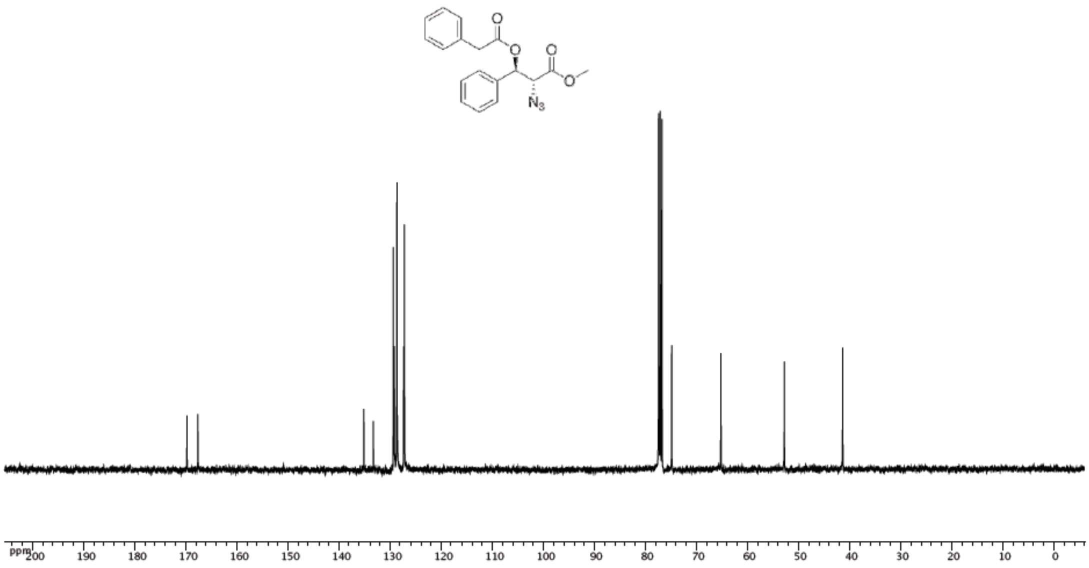

^{1}H- and ^{13}C-NMR of compound (±)-**8d** in $CDCl_3$

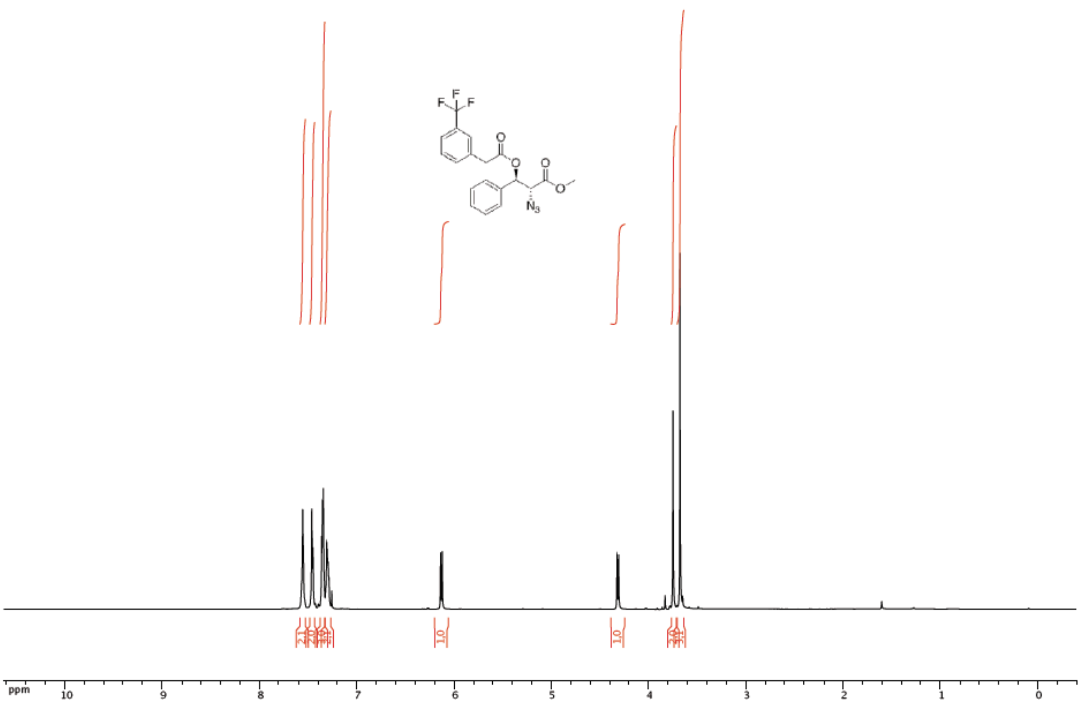

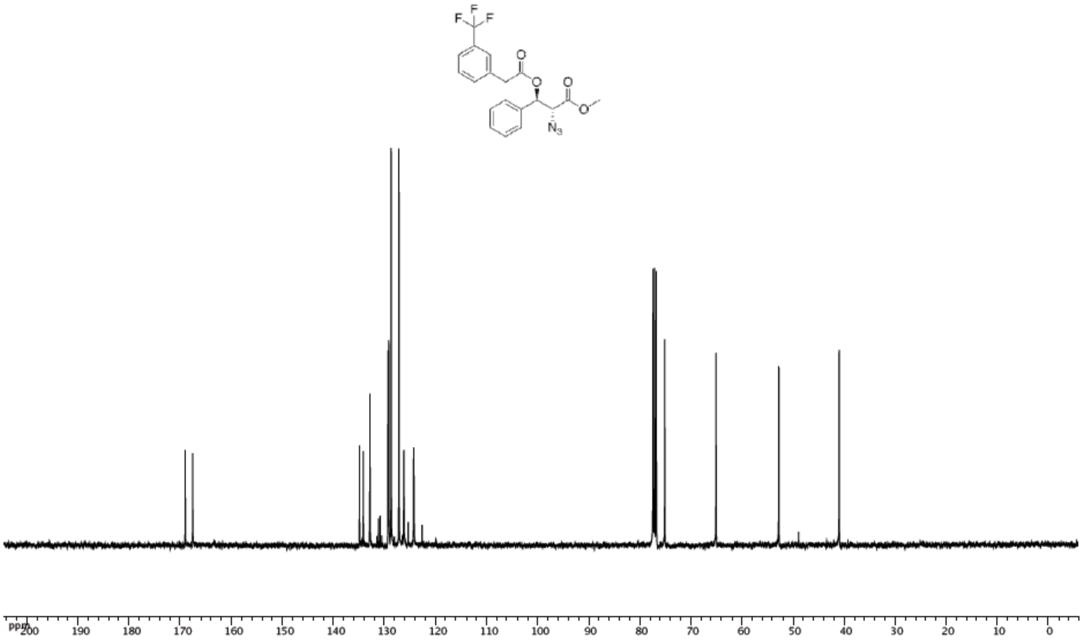

^{1}H- and ^{13}C-NMR of compound (±)-**8e** in $CDCl_3$

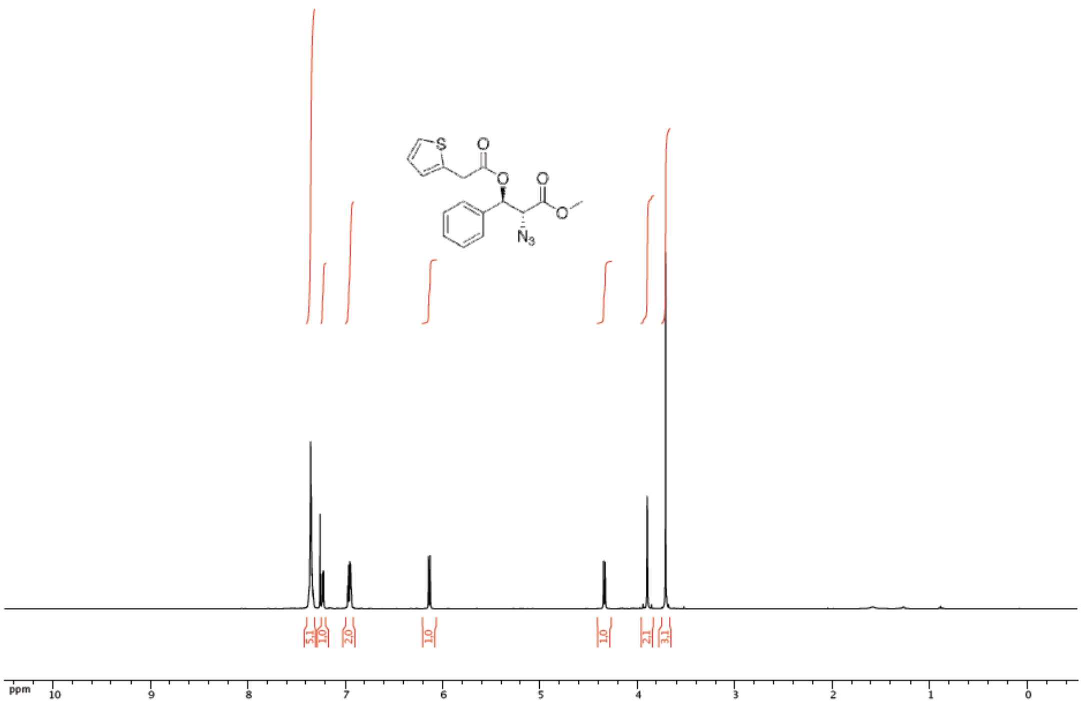

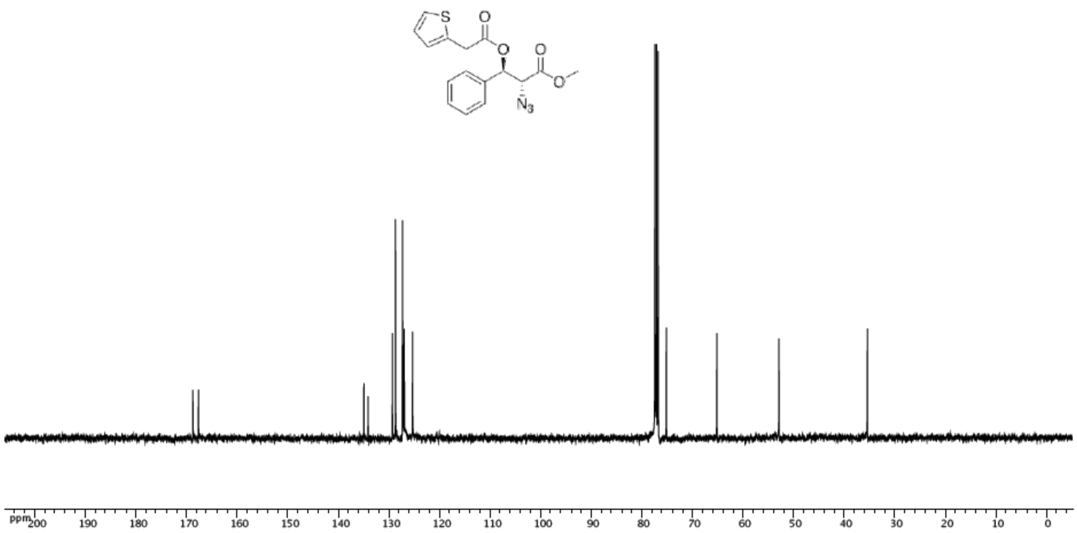

^{1}H- and ^{13}C-NMR of compound (±)-**9b** in $CDCl_3$

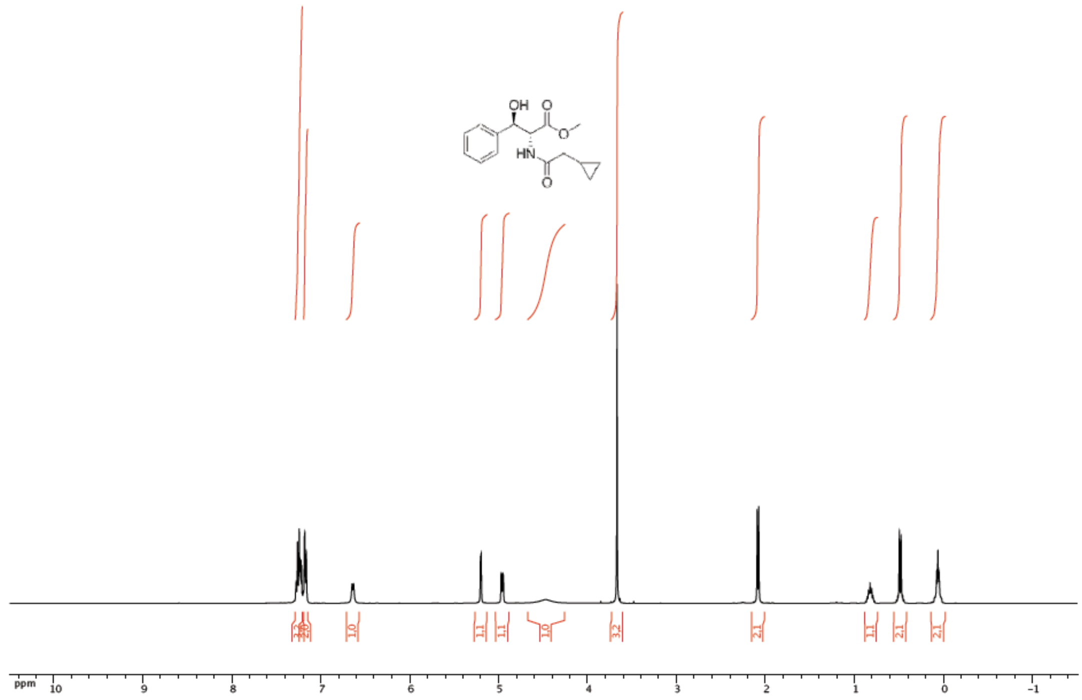

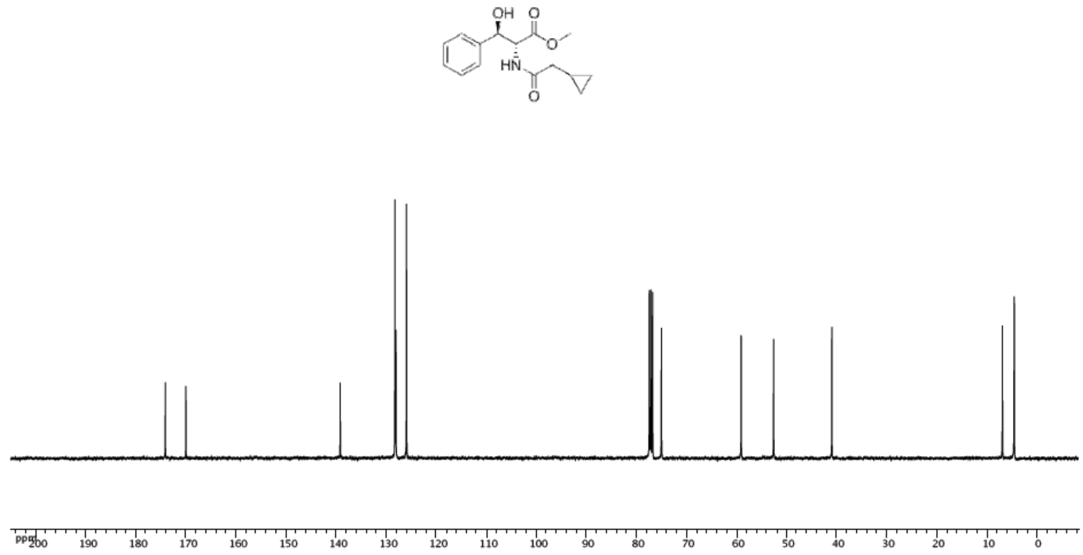

^{1}H- and ^{13}C-NMR of compound (±)-**9c** in $CDCl_3$

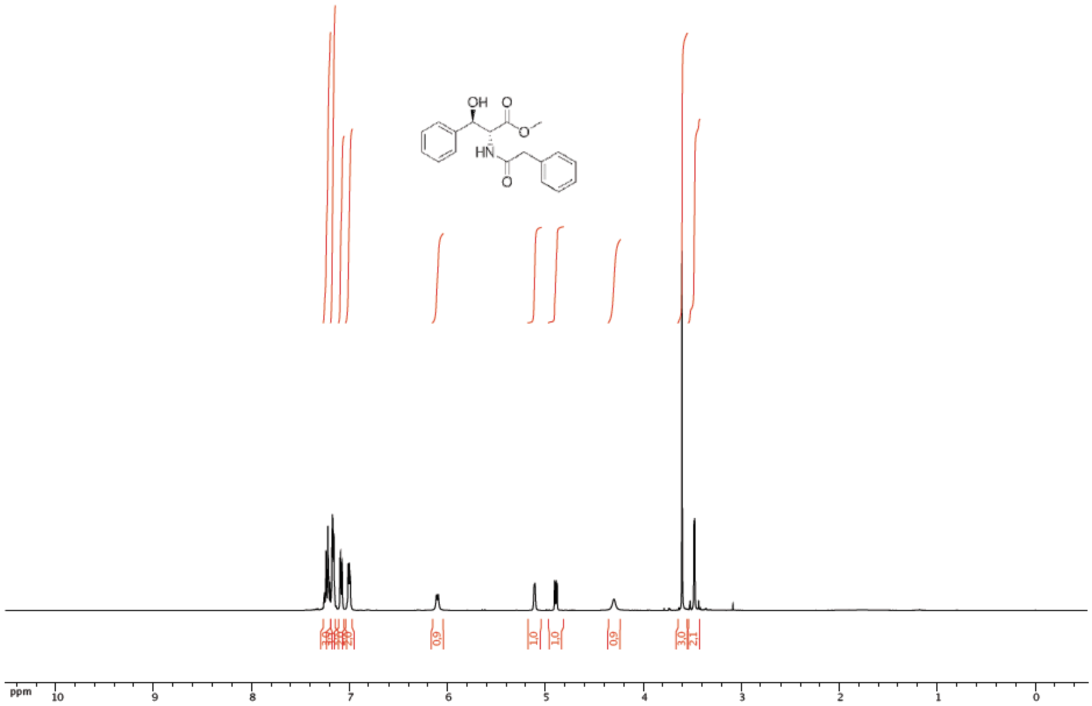

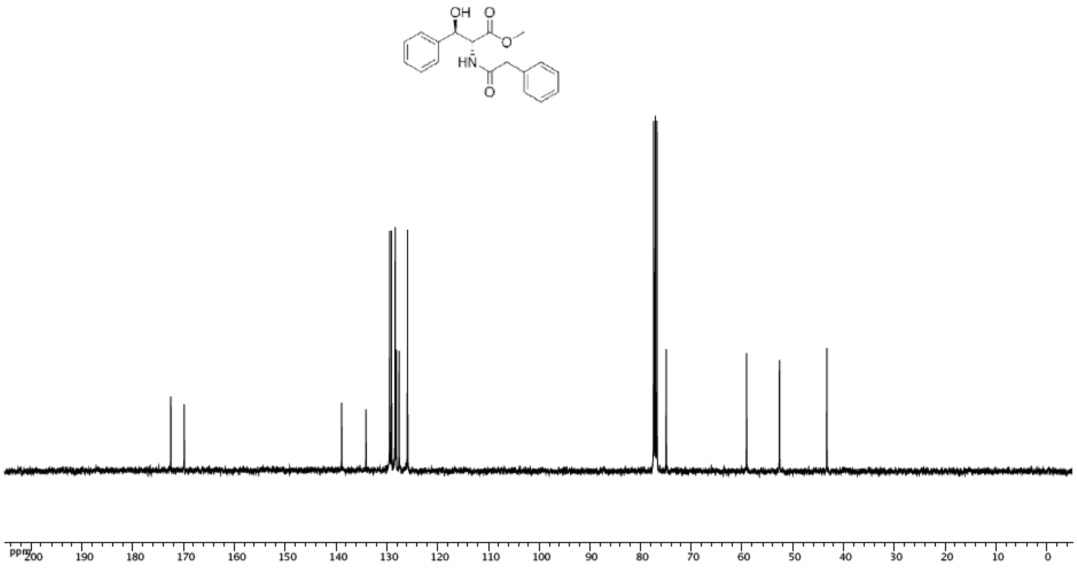

^{1}H- and ^{13}C-NMR of compound (±)-**9d** in $CDCl_3$

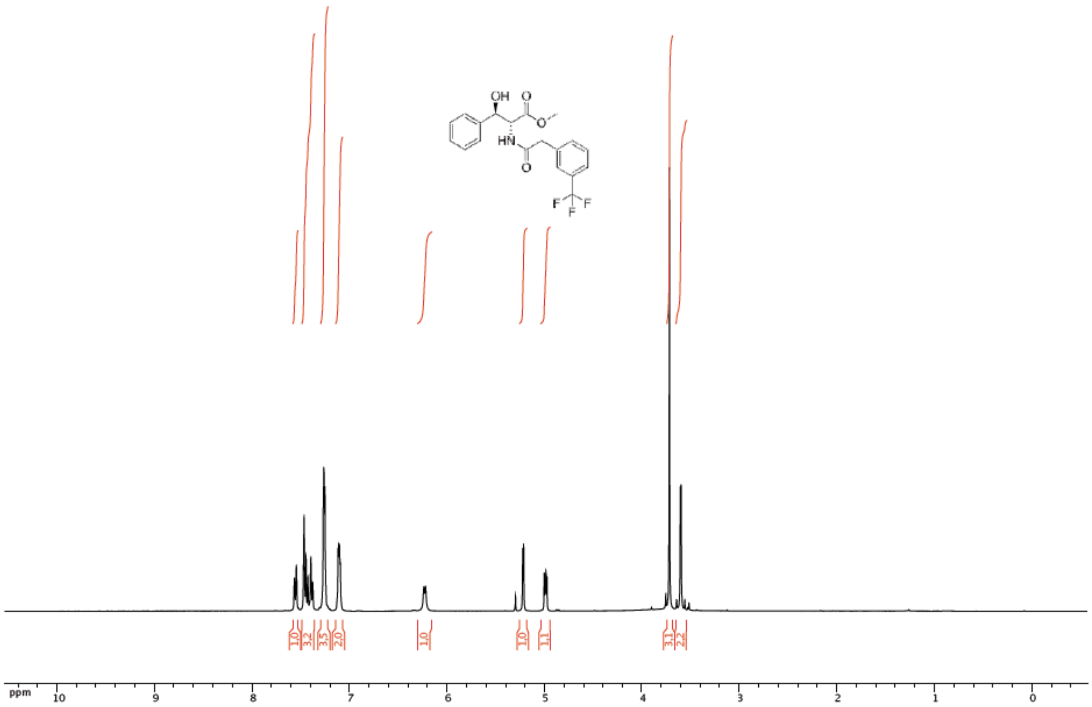

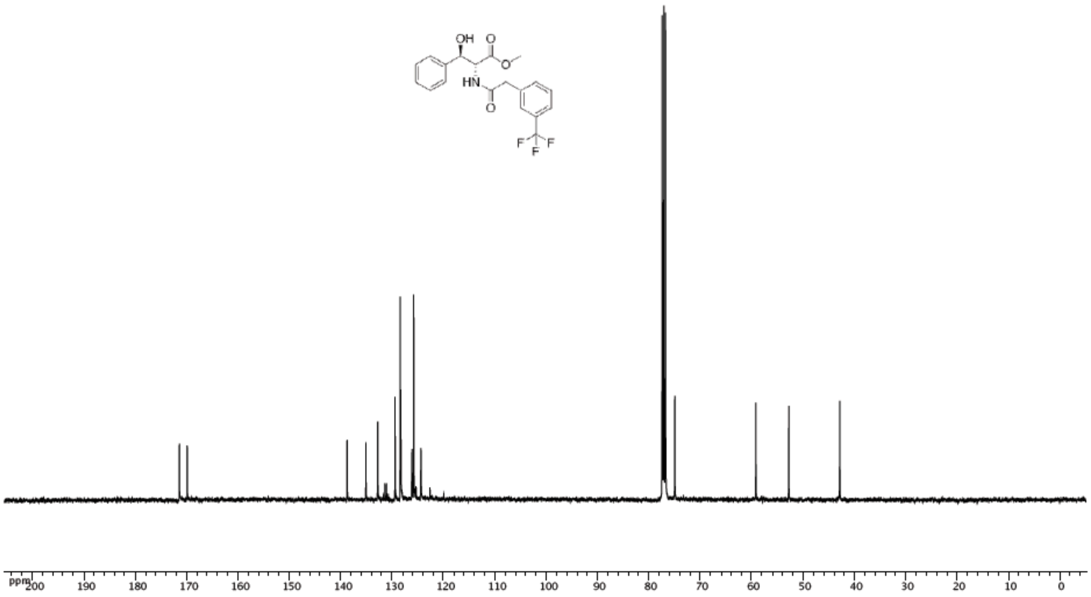

^{1}H- and ^{13}C-NMR of compound (±)-**9e** in $CDCl_3$

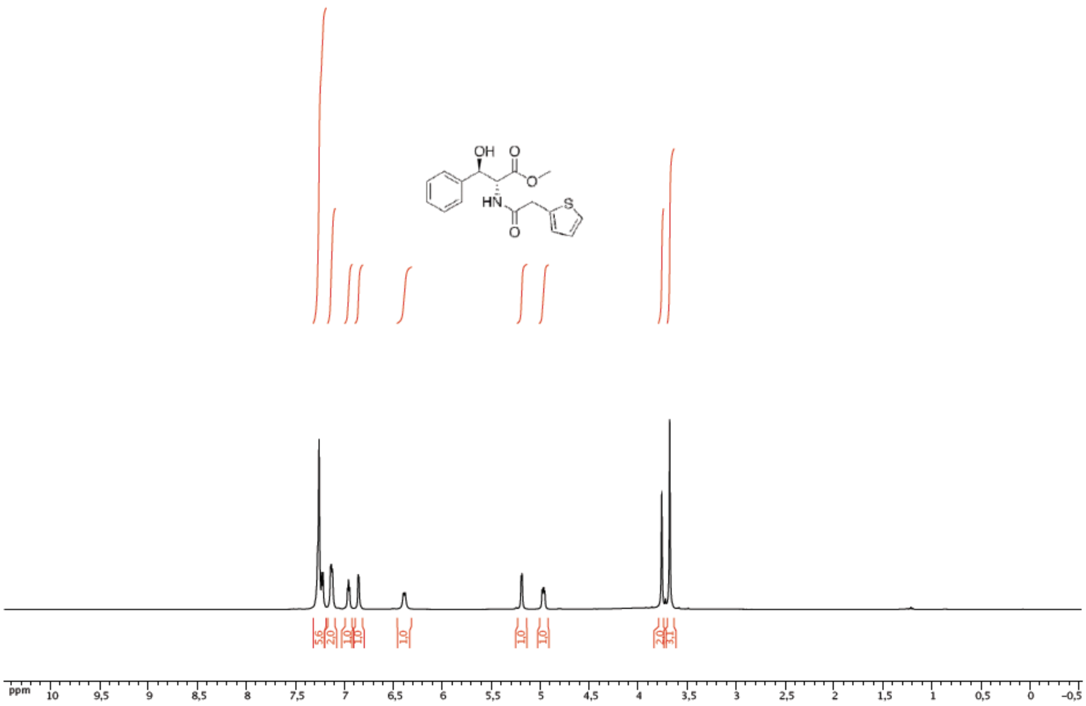

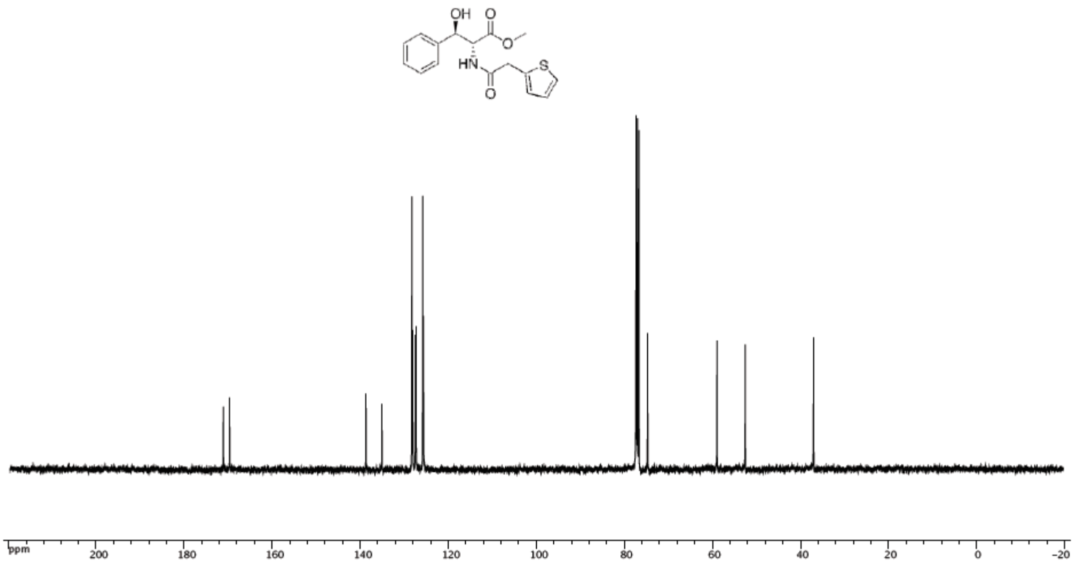

^{1}H- and ^{13}C-NMR of compound (±)-**10a** in $CDCl_3$

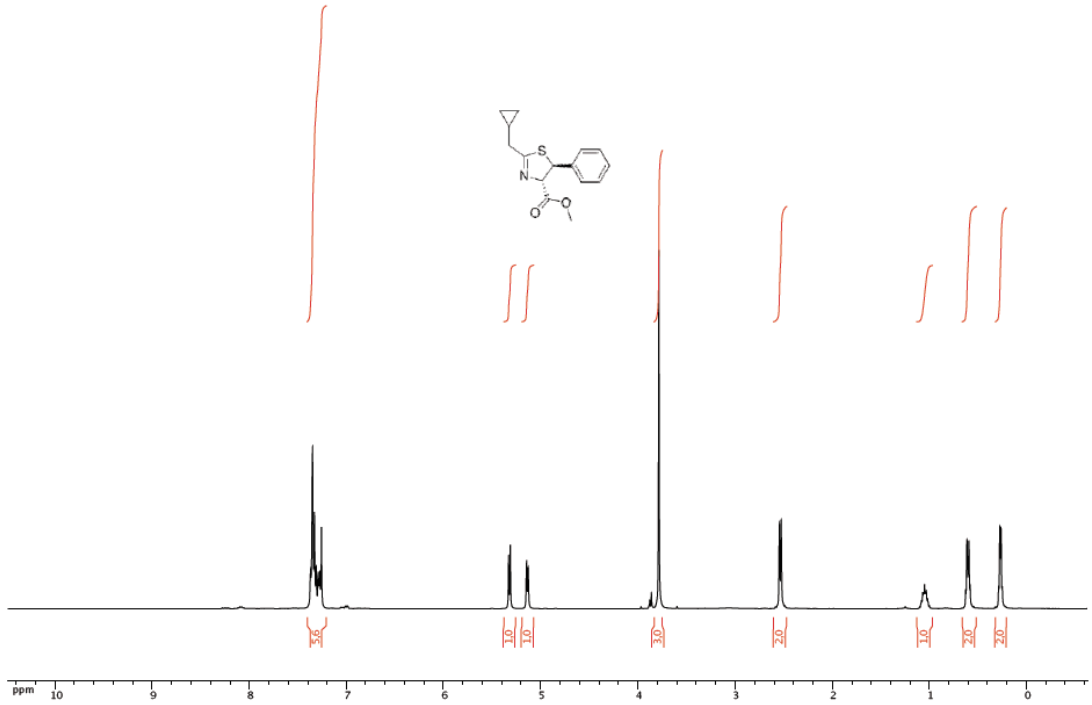

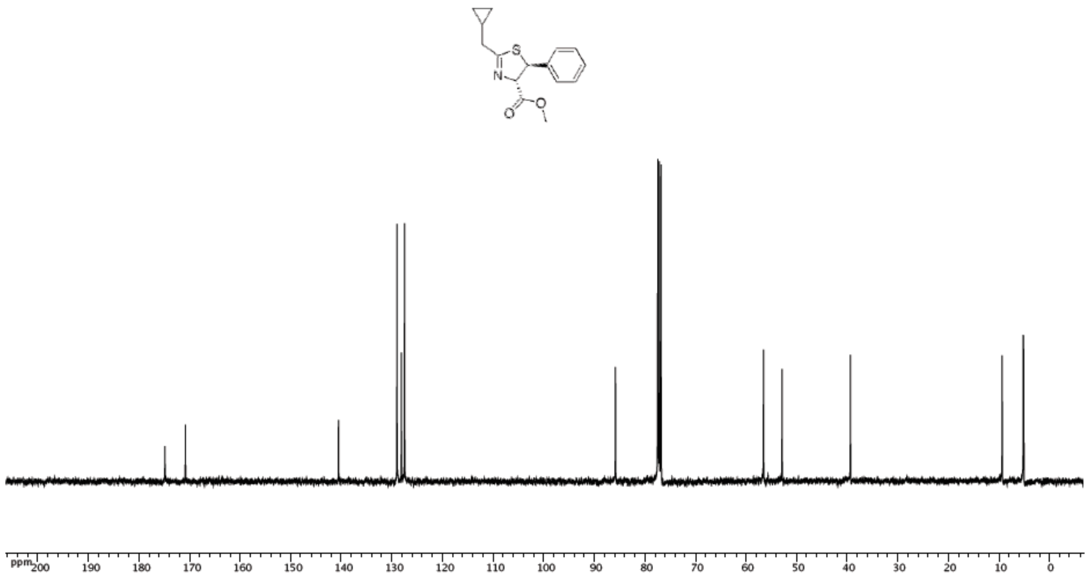

^{1}H- and ^{13}C-NMR of compound (±)-**10b** in $CDCl_3$

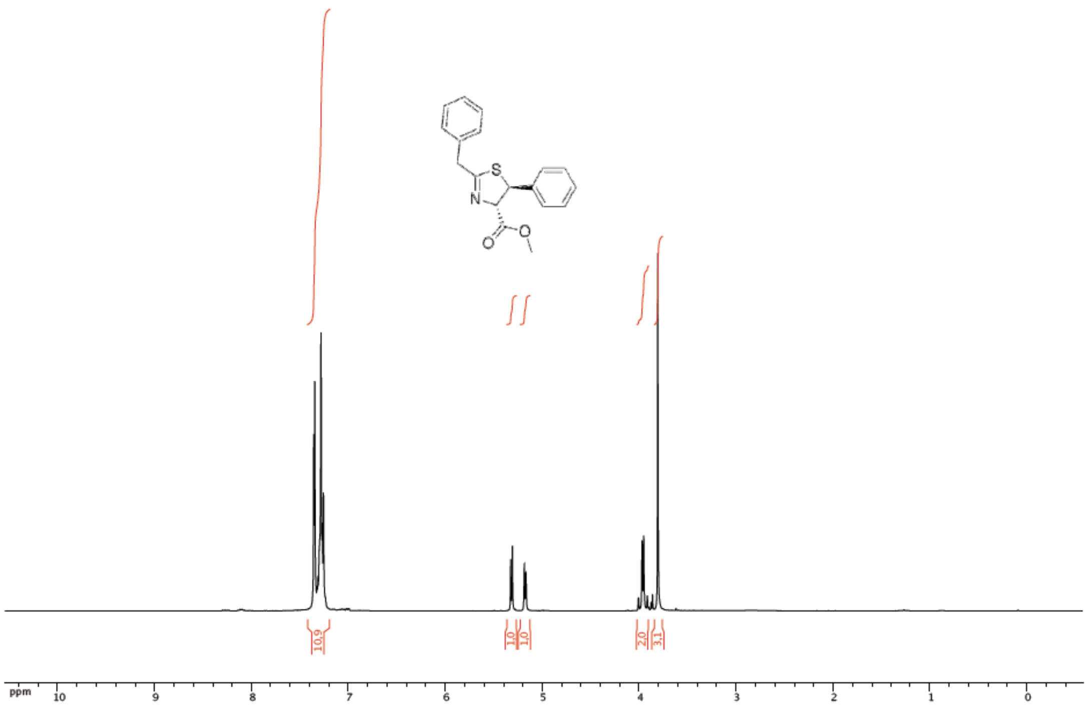

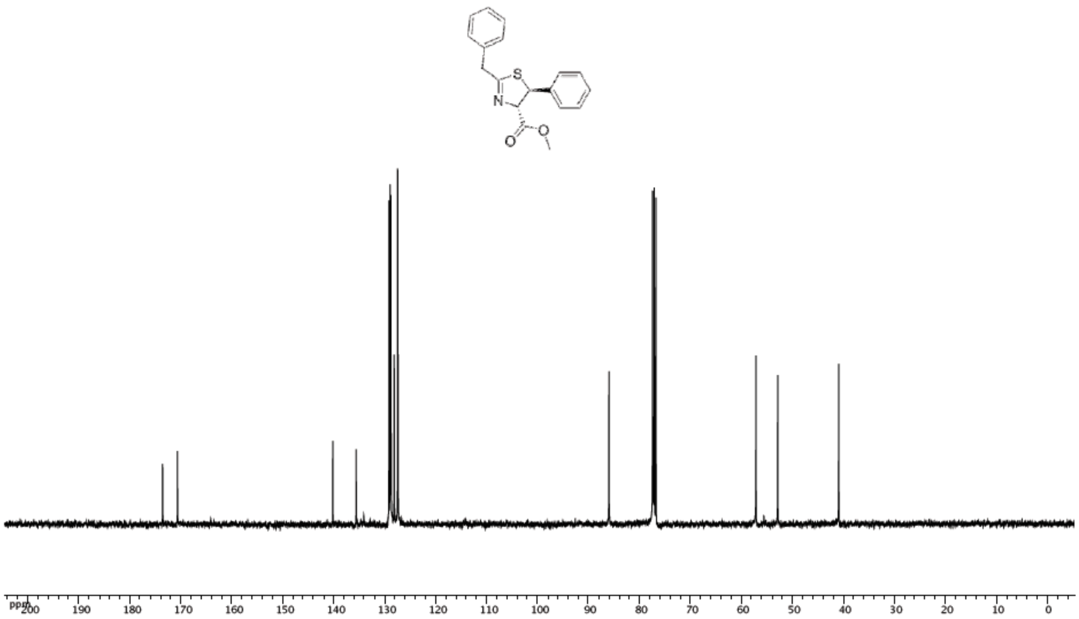

^{1}H- and ^{13}C-NMR of compound (±)-**10c** in $CDCl_3$

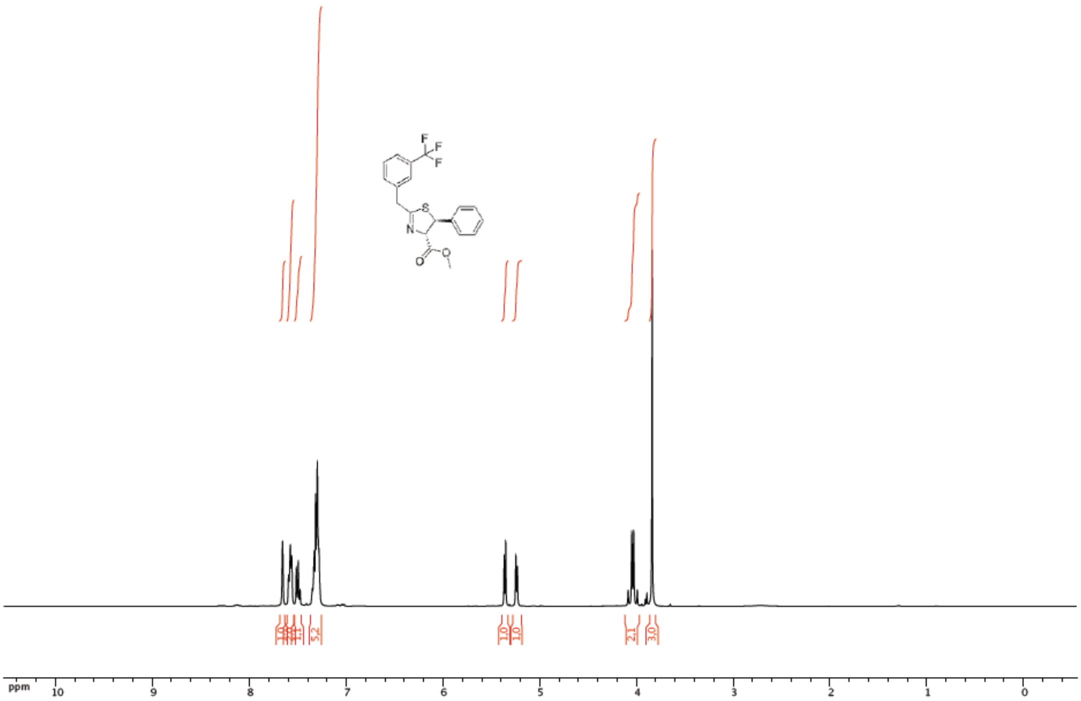

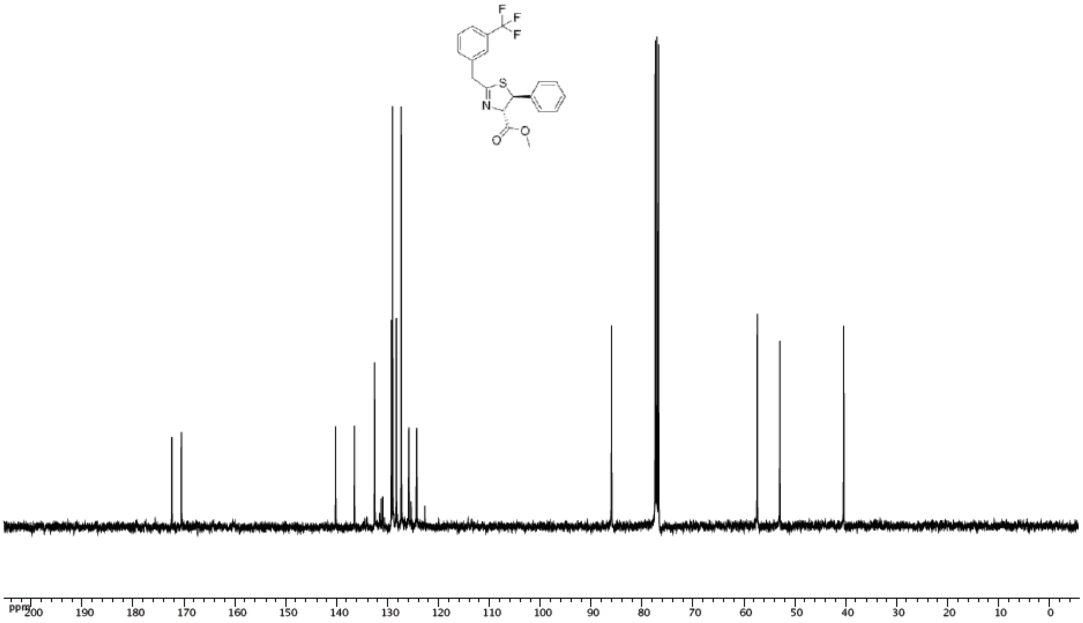

^{1}H- and ^{13}C-NMR of compound (±)-**10d** in $CDCl_3$

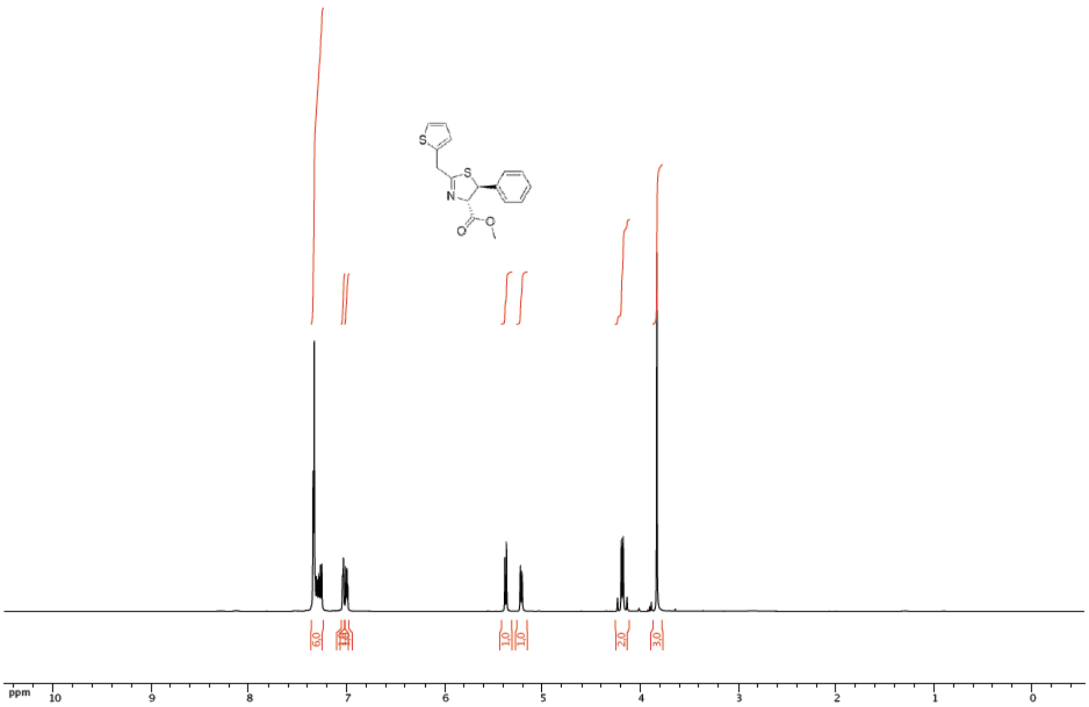

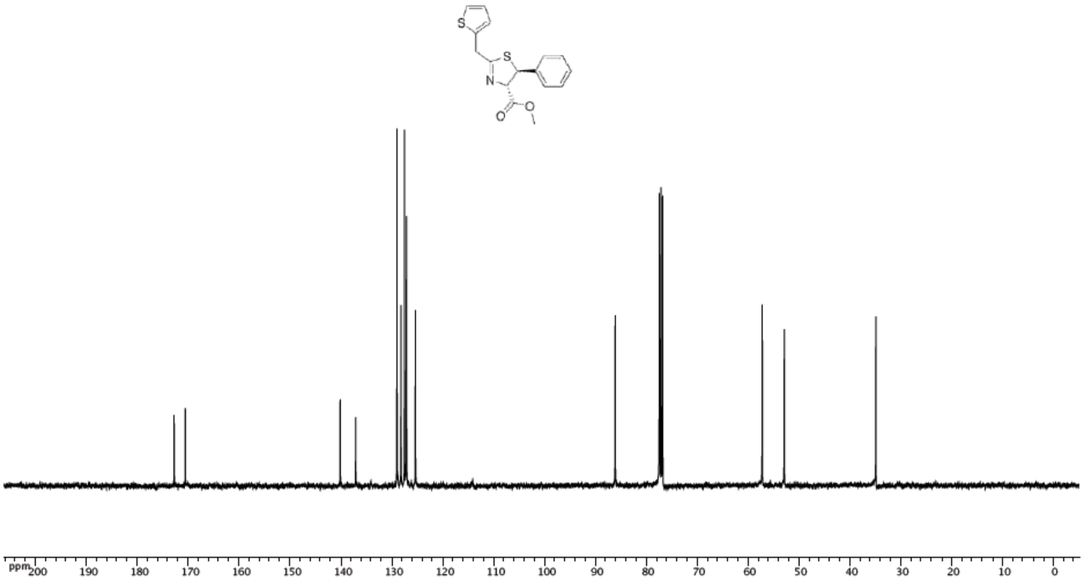

^{1}H- and ^{13}C-NMR of compound (±)-**12b** in $CDCl_3$

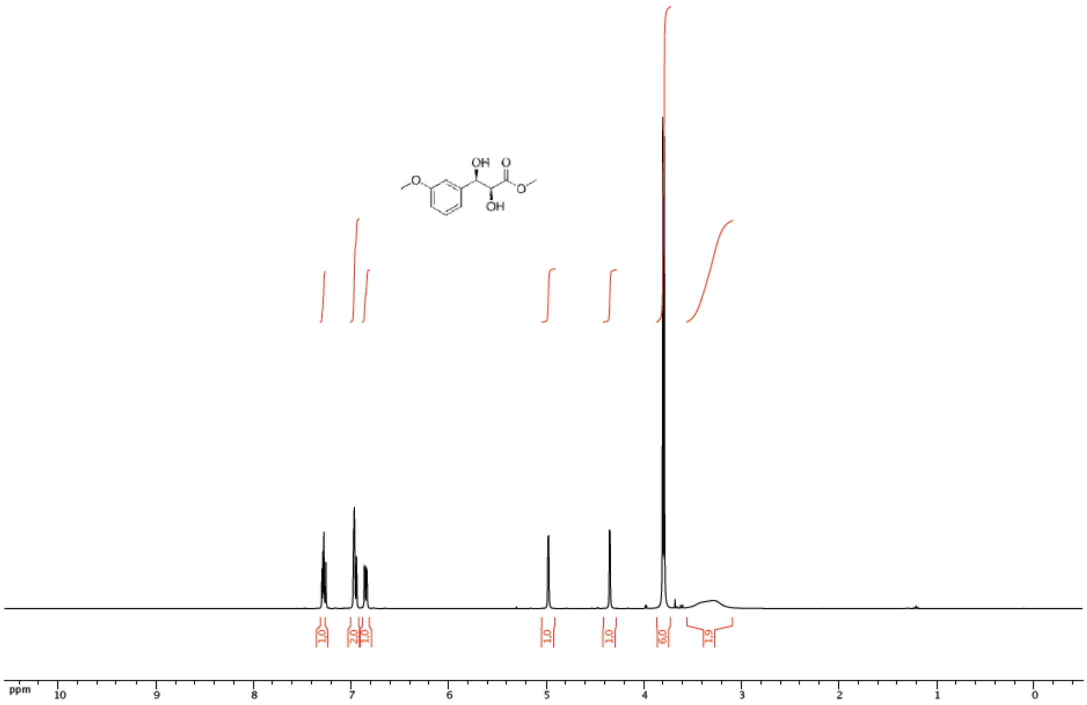

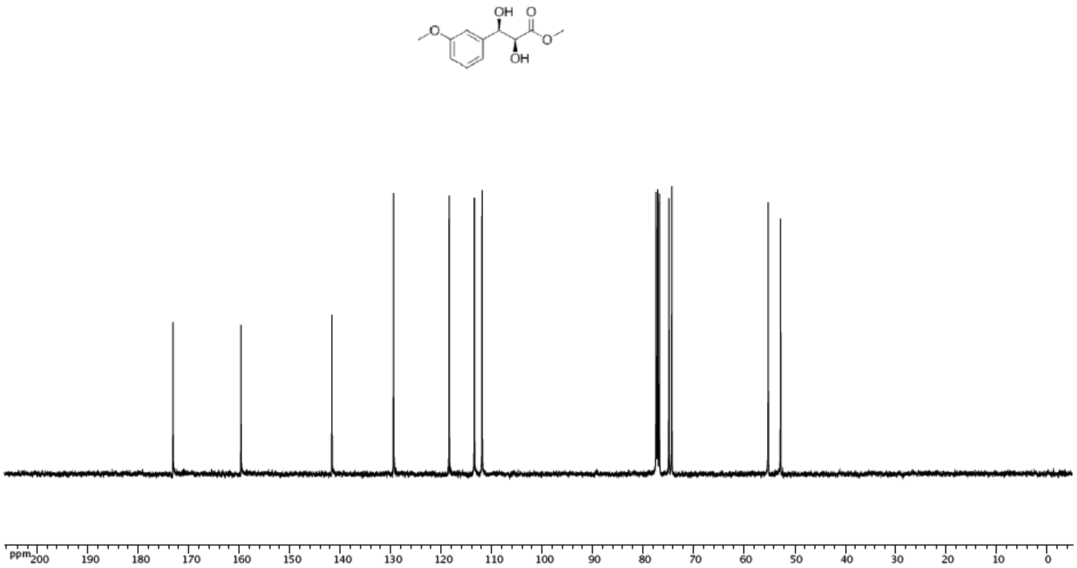

^{1}H- and ^{13}C-NMR of compound (±)-**12d** in $CDCl_3$

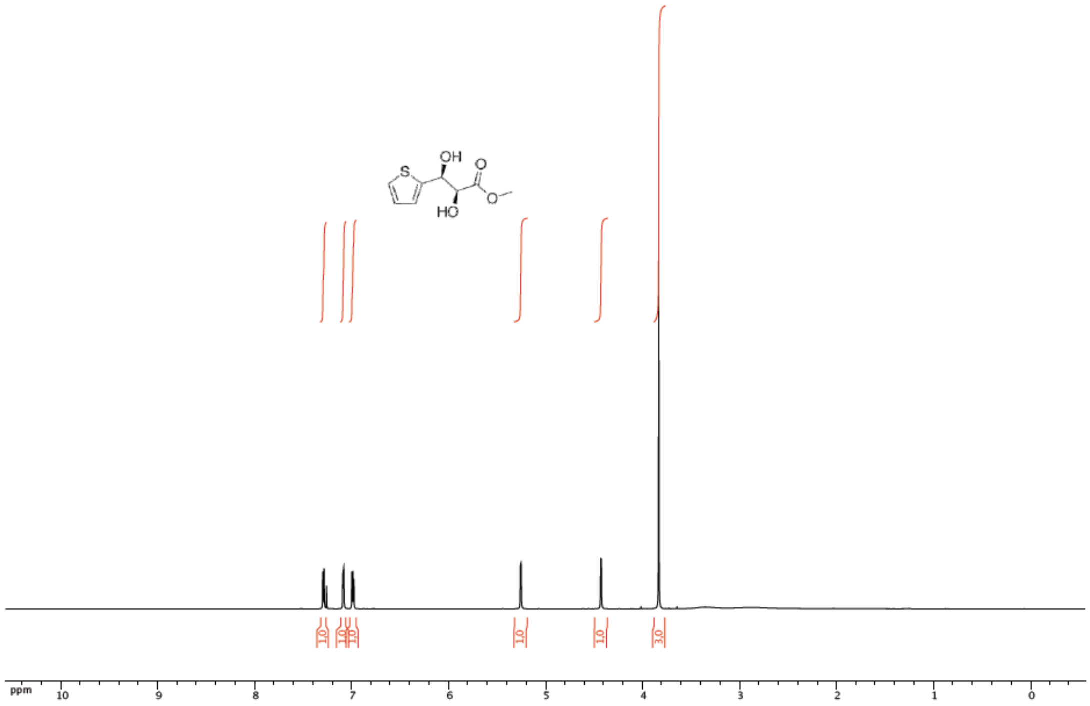

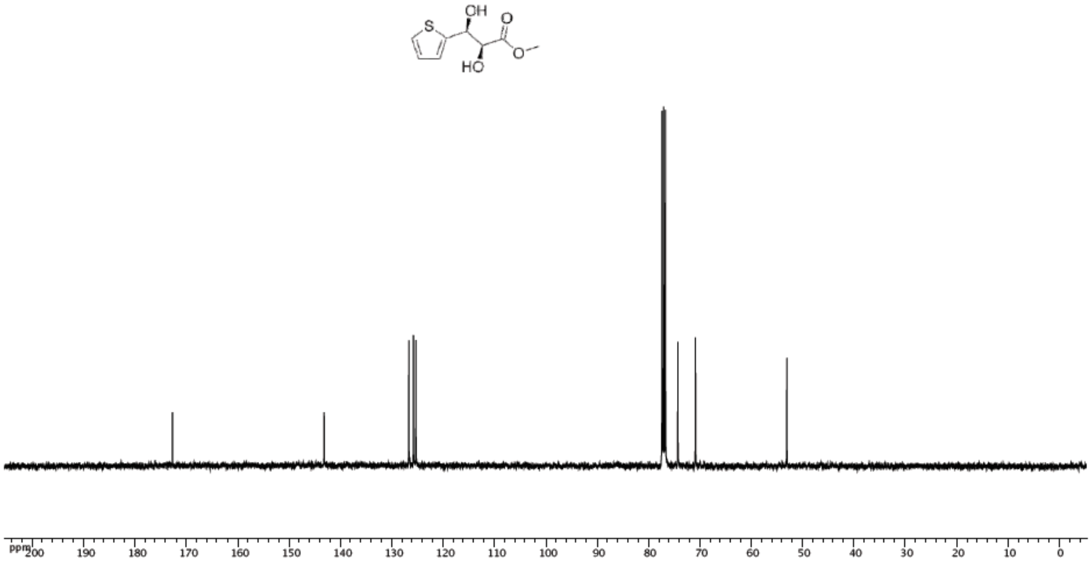

^{1}H-NMR of compound (±)-**13a** in $CDCl_3$ and ^{13}C-NMR in $CDCl_3$:d_6-DMSO 1:1

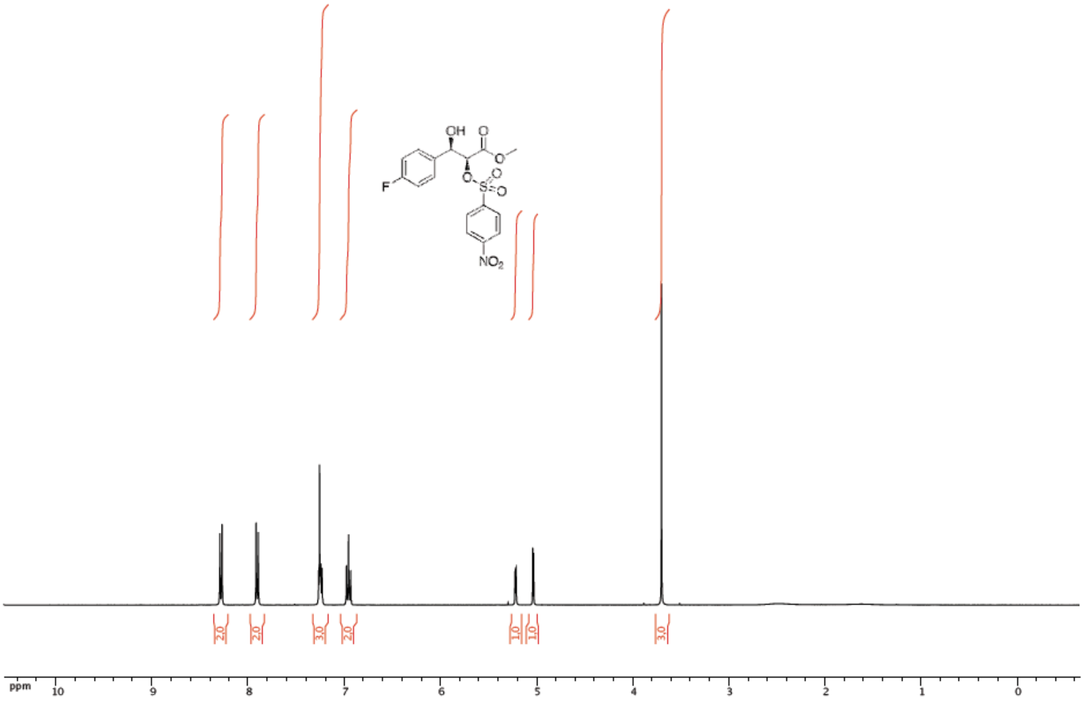

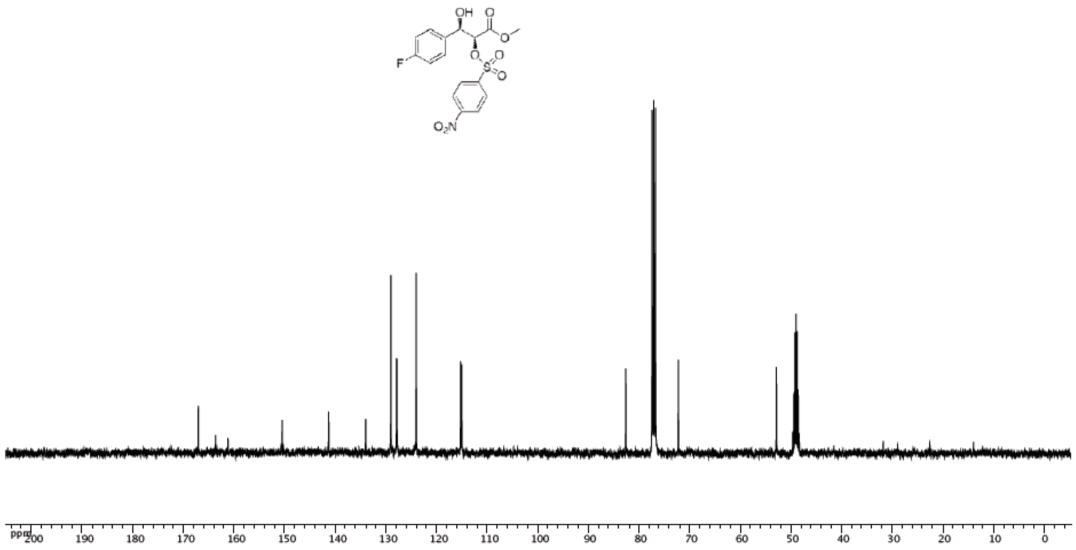

^{1}H-NMR of compound (±)-**13b** in $CDCl_3$ and ^{13}C-NMR in d_6-DMSO

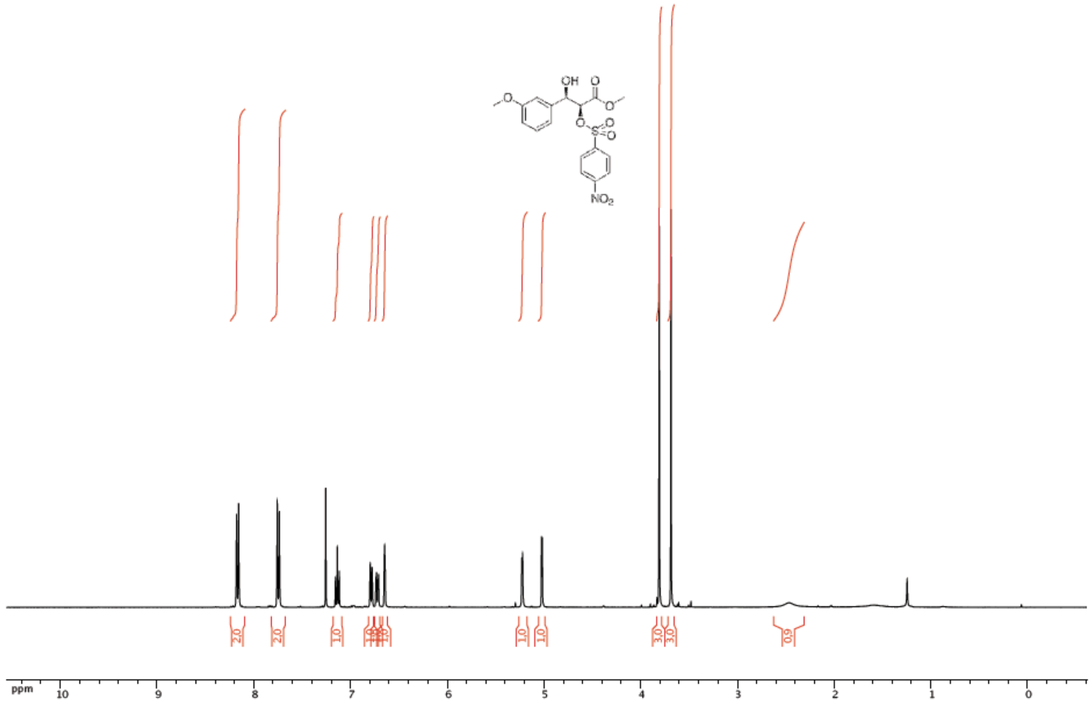

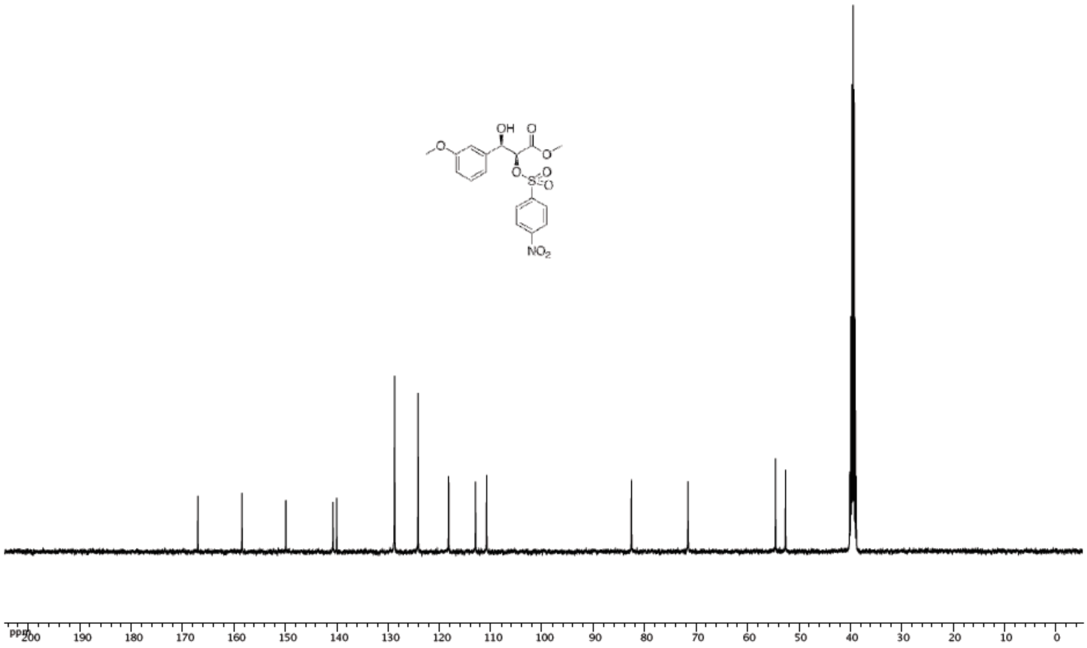

^{1}H- and ^{13}C-NMR of compound (±)-**13c** in $CDCl_3$

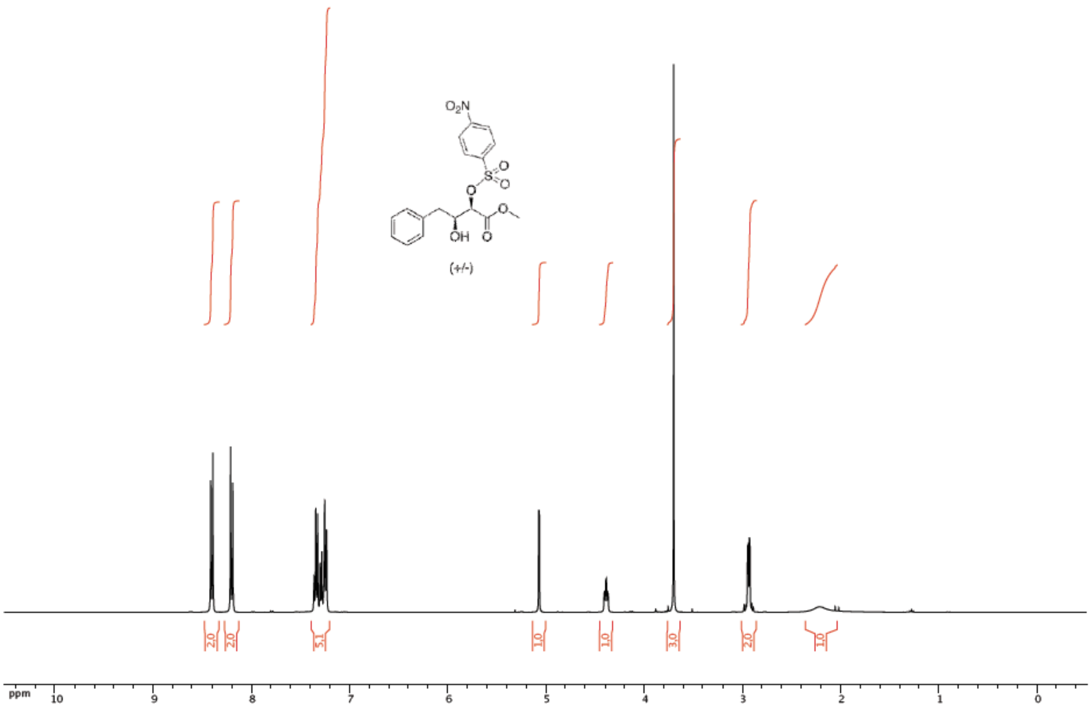

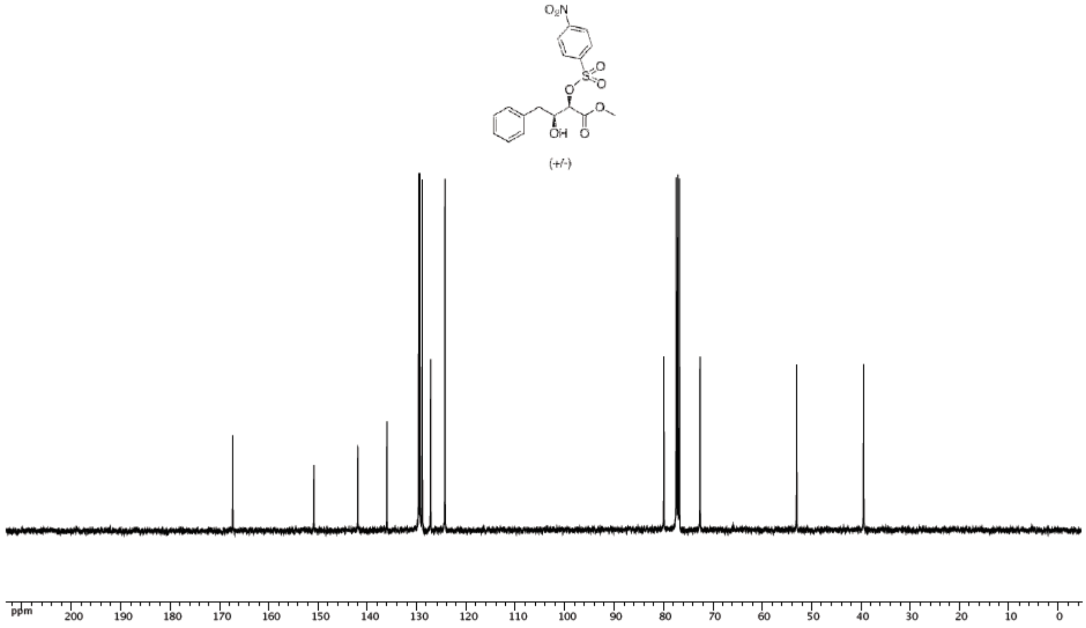

^{1}H- and ^{13}C-NMR of compound (±)-**13d** in $CDCl_3$

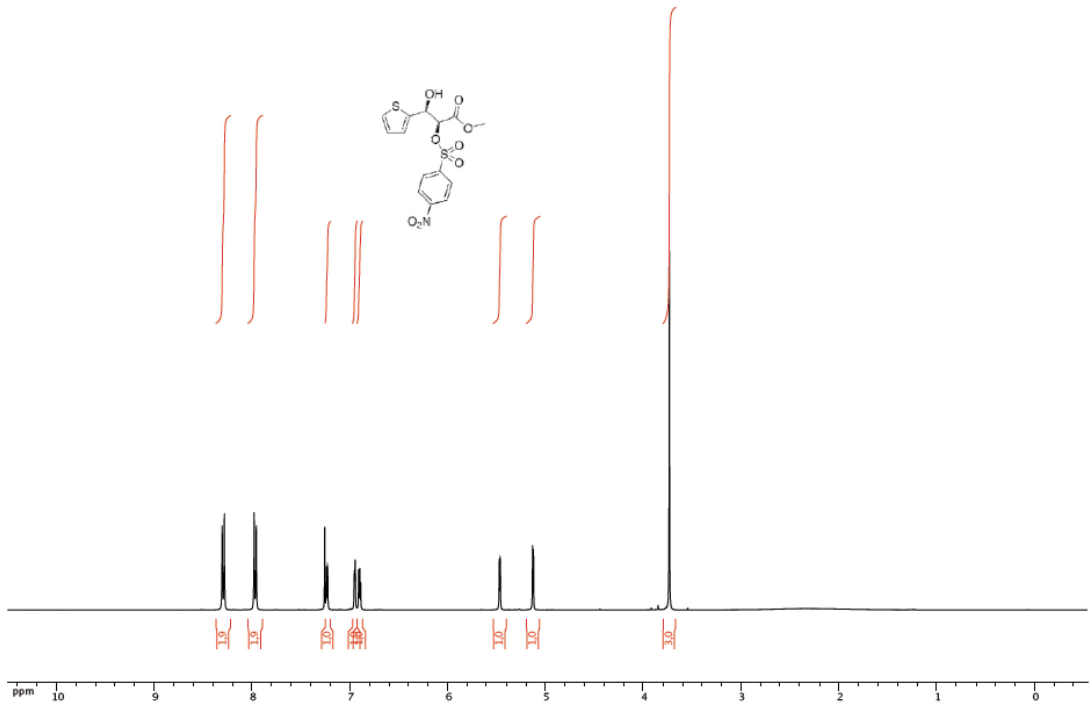

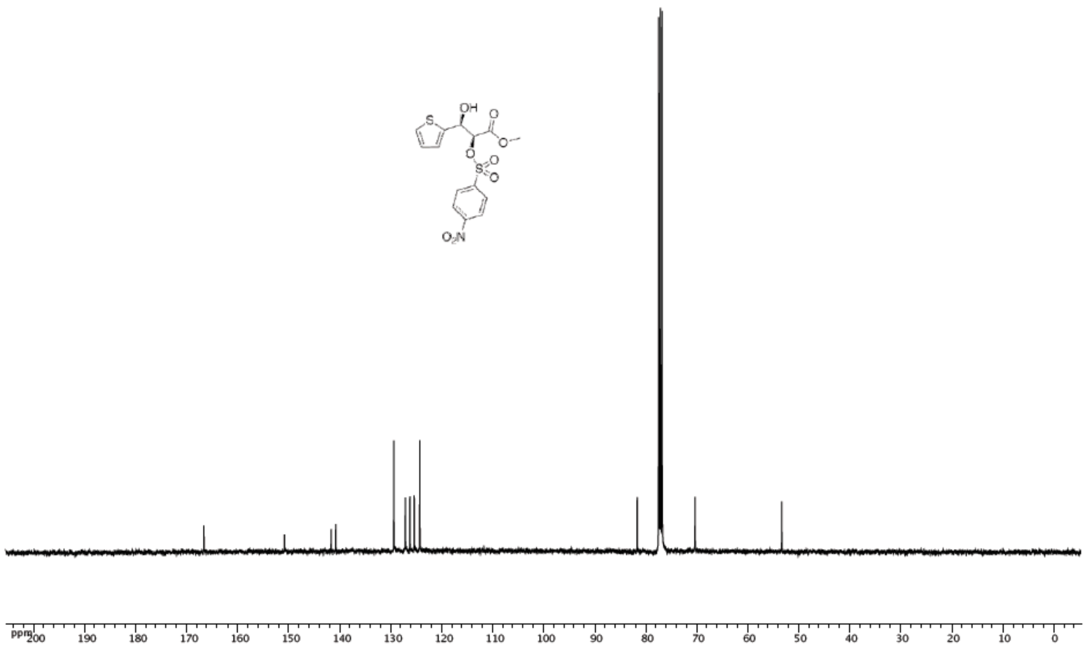

^{1}H- and ^{13}C-NMR of compound (±)-**14a** in $CDCl_3$

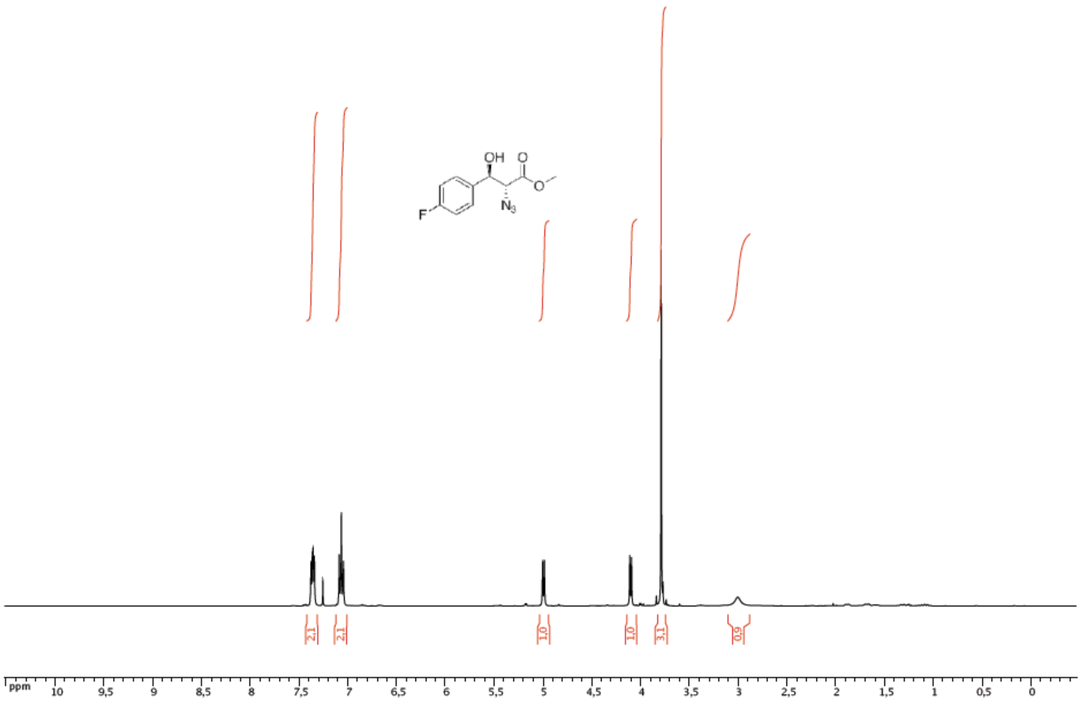

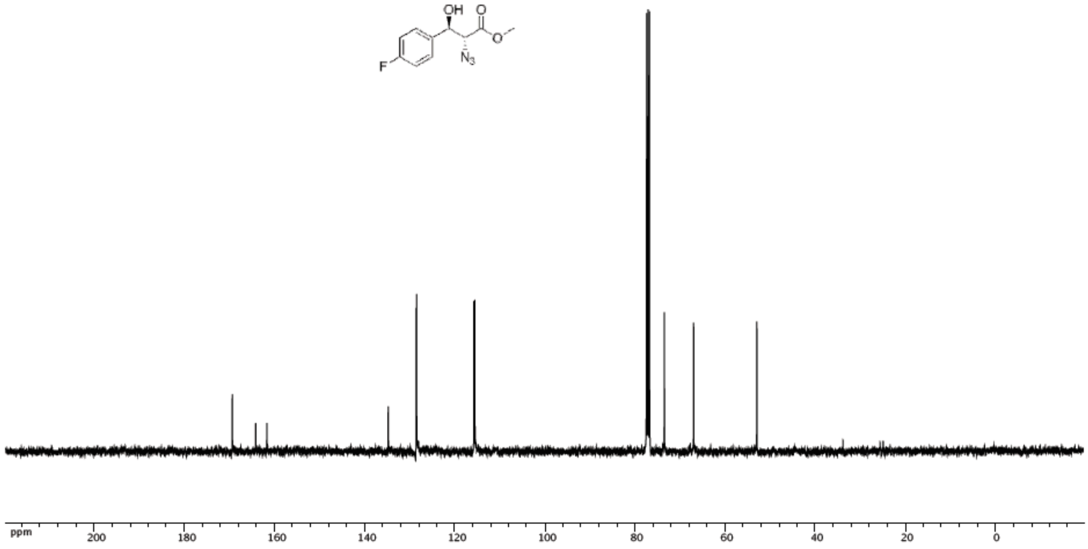

^{1}H- and ^{13}C-NMR of compound (±)-**14b** in $CDCl_3$

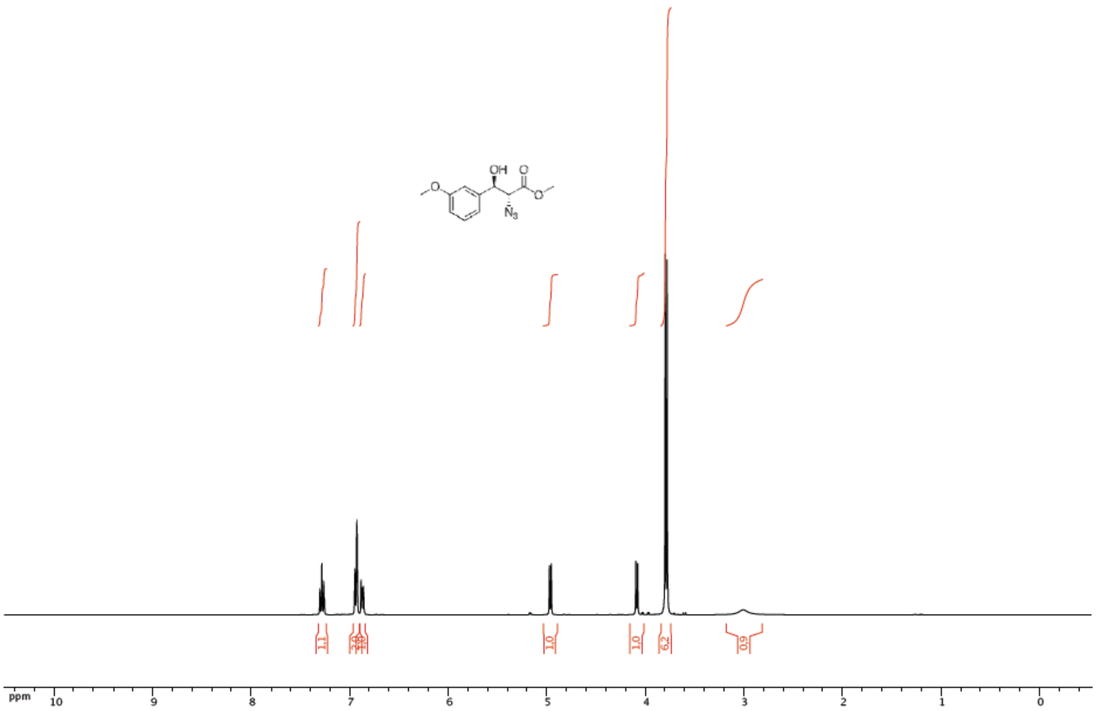

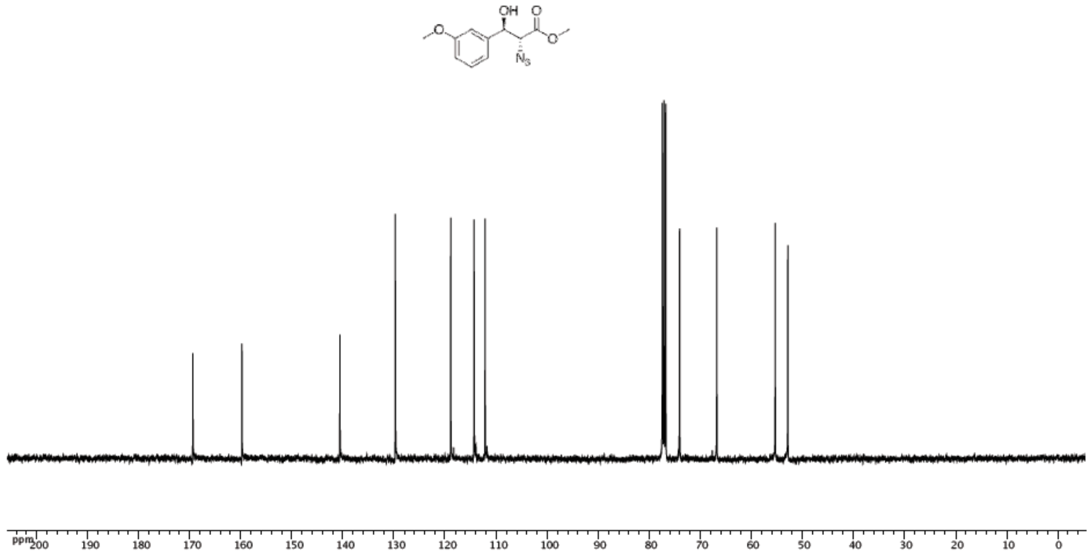

^{1}H- and ^{13}C-NMR of compound (±)-**14c** in $CDCl_3$

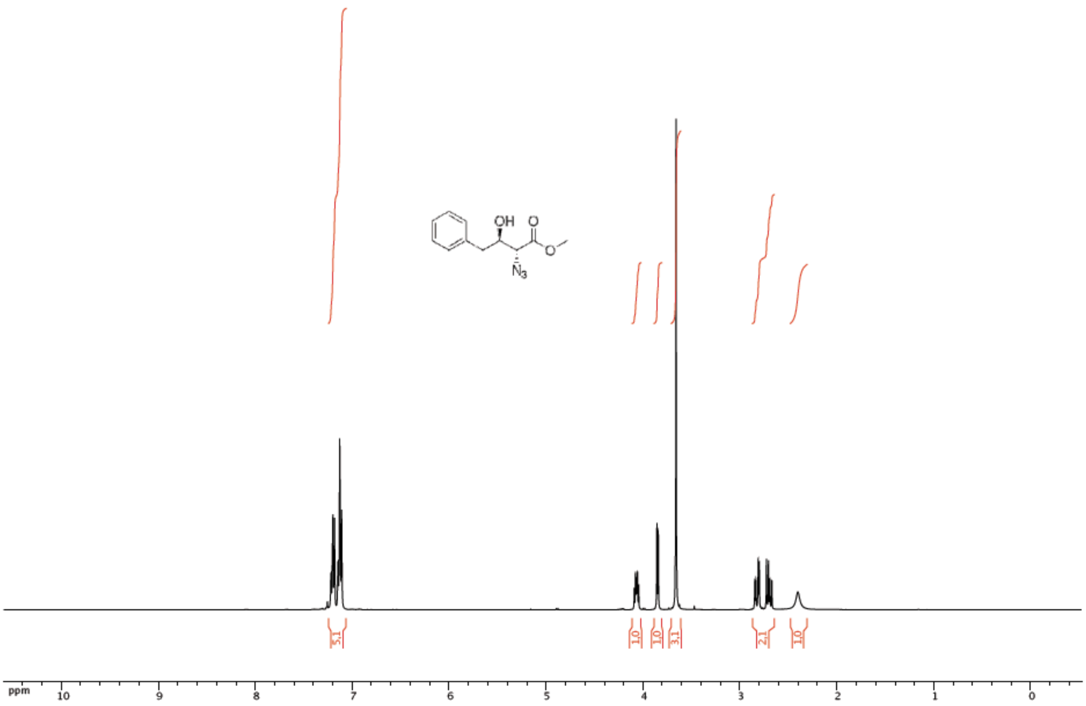

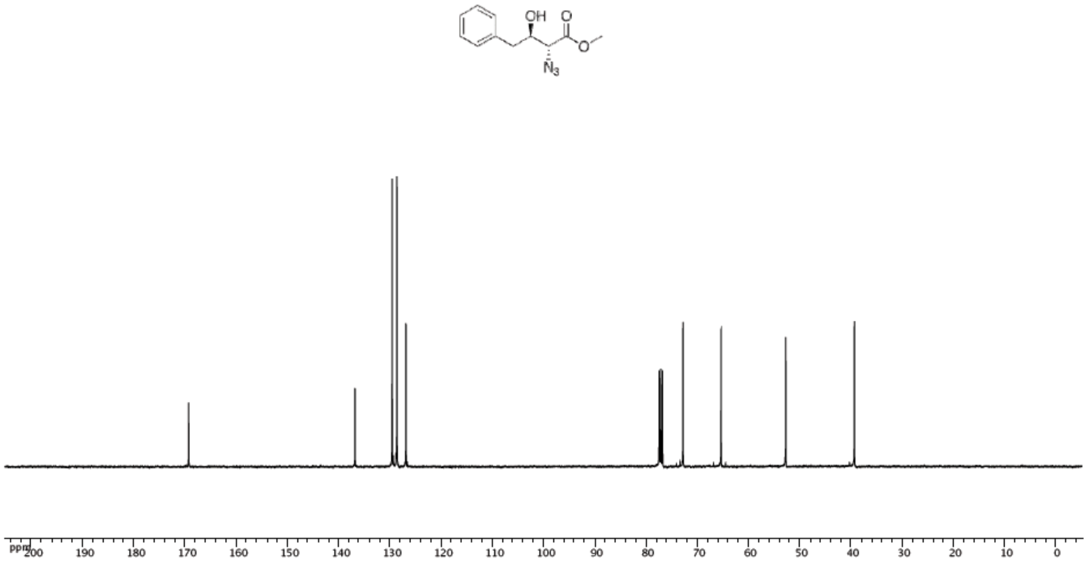

^{1}H- and ^{13}C-NMR of compound (±)-**14d** in $CDCl_3$

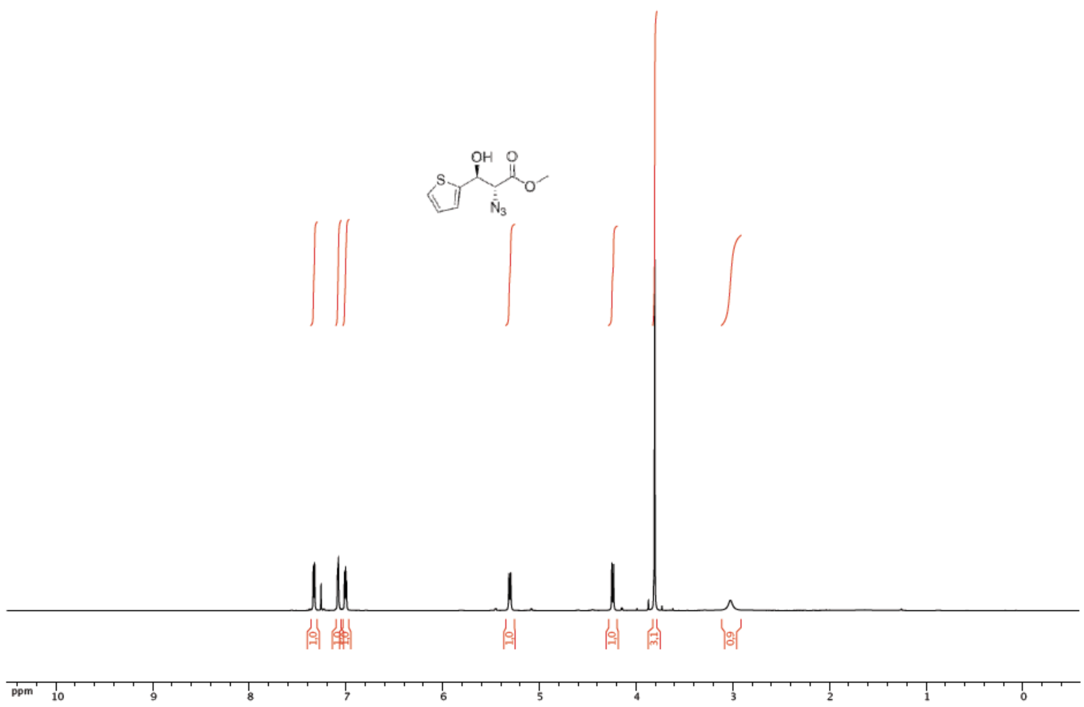

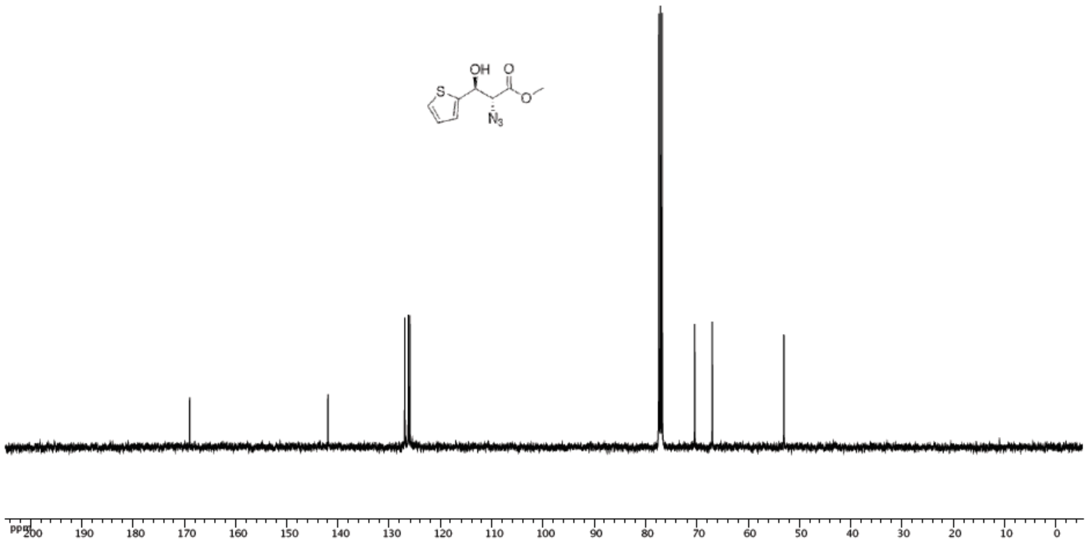

^{1}H- and ^{13}C-NMR of compound (±)-**15a** in $CDCl_3$

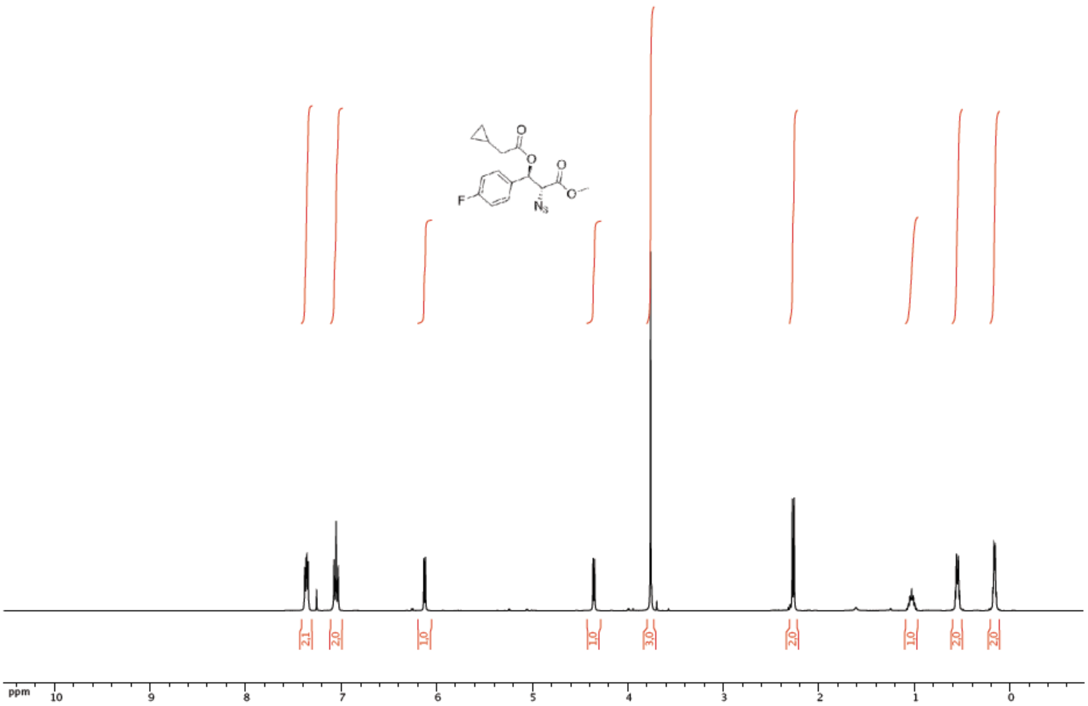

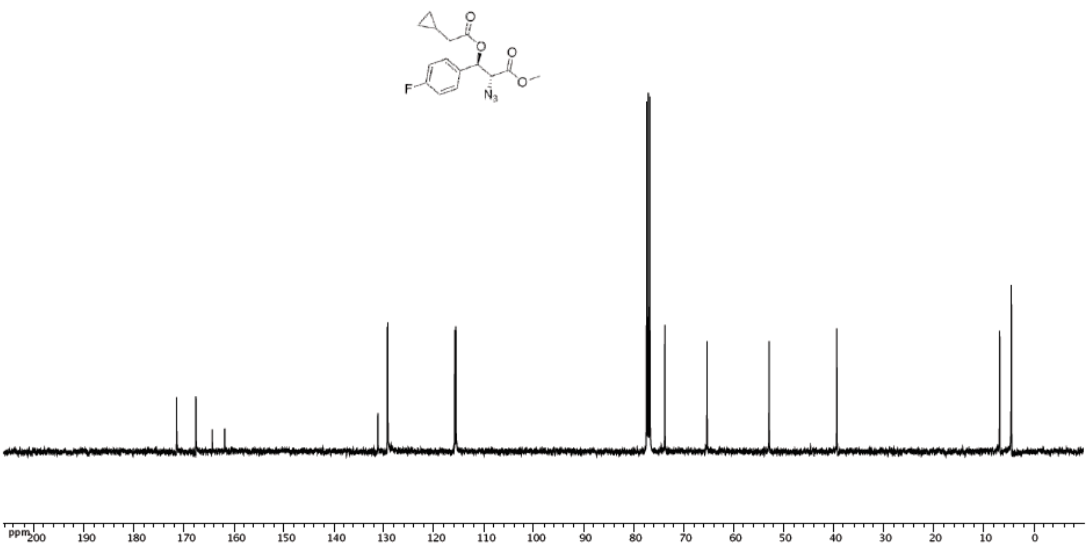

^{1}H- and ^{13}C-NMR of compound (±)-**15b** in $CDCl_3$

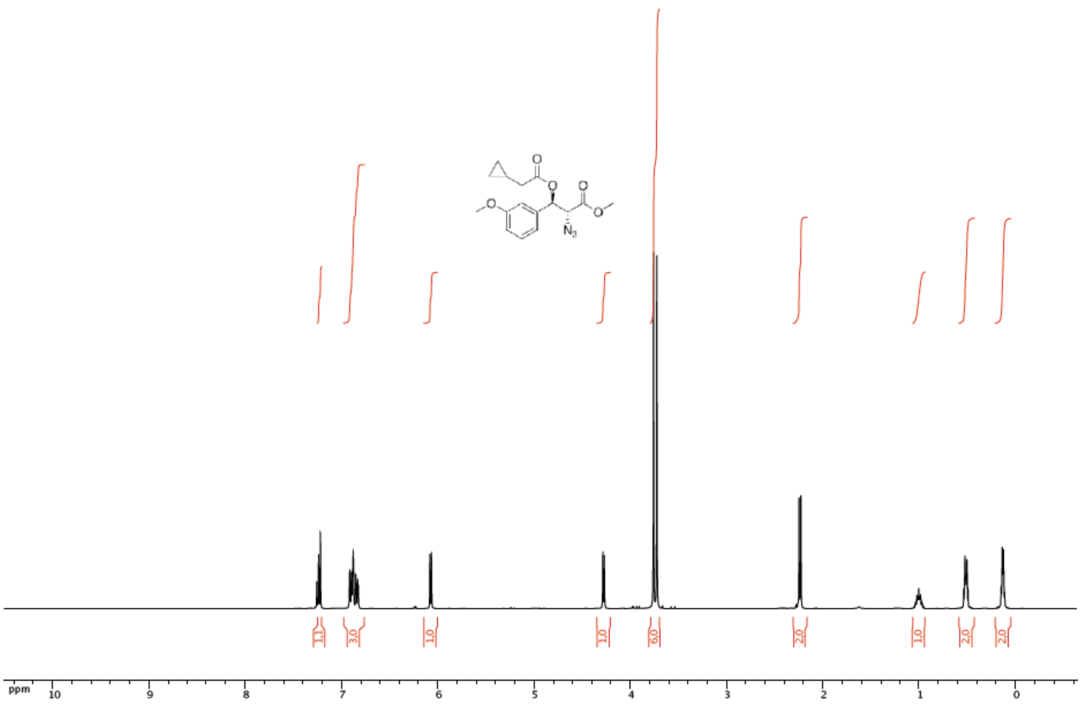

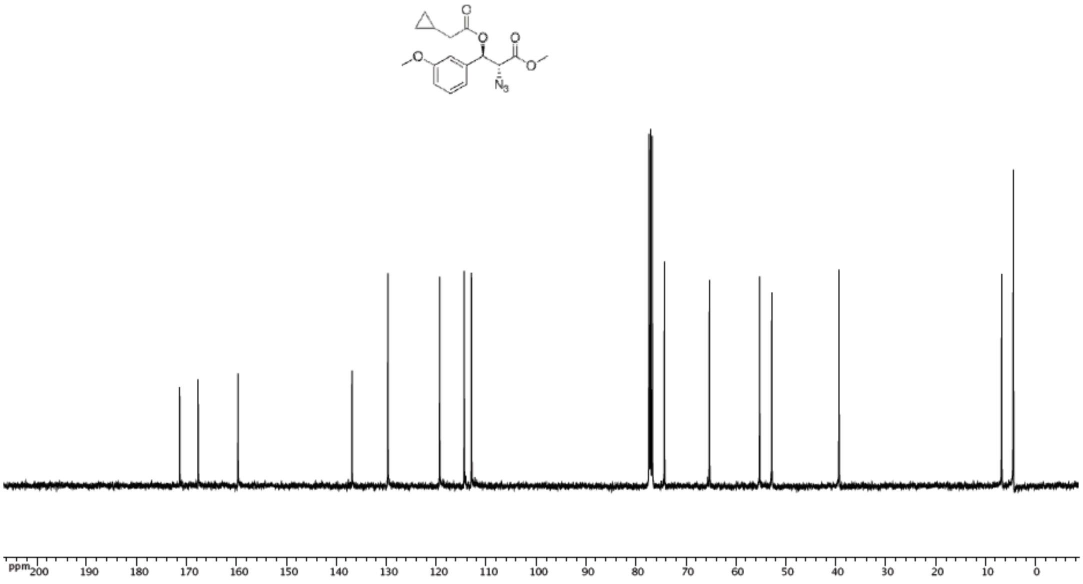

^{1}H- and ^{13}C-NMR of compound (±)-**15c** in $CDCl_3$

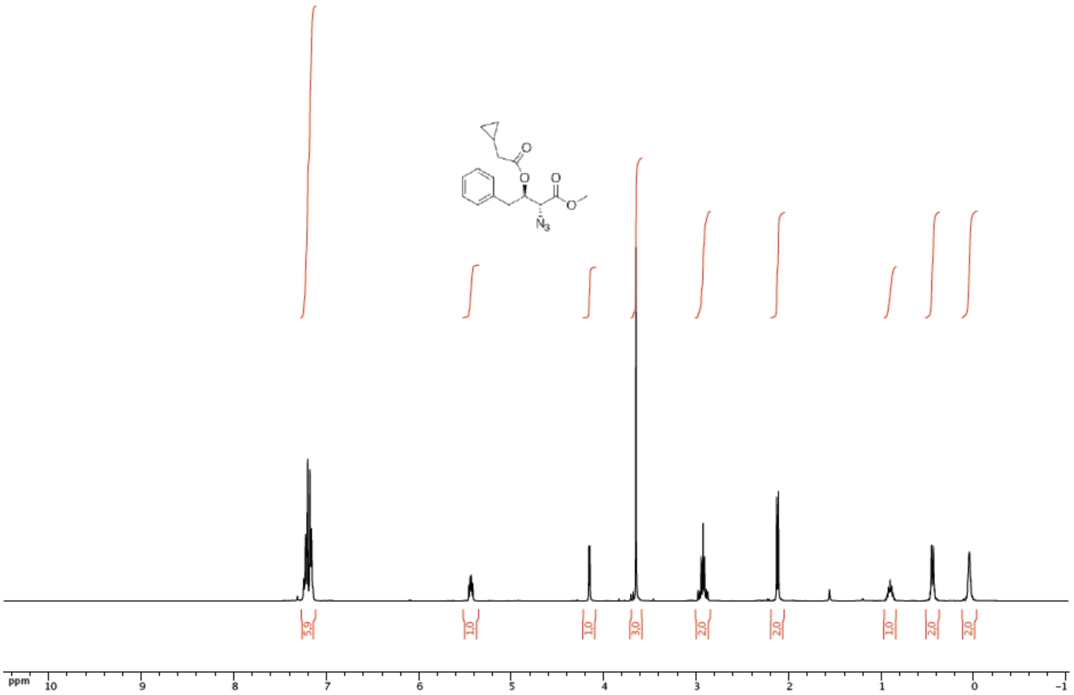

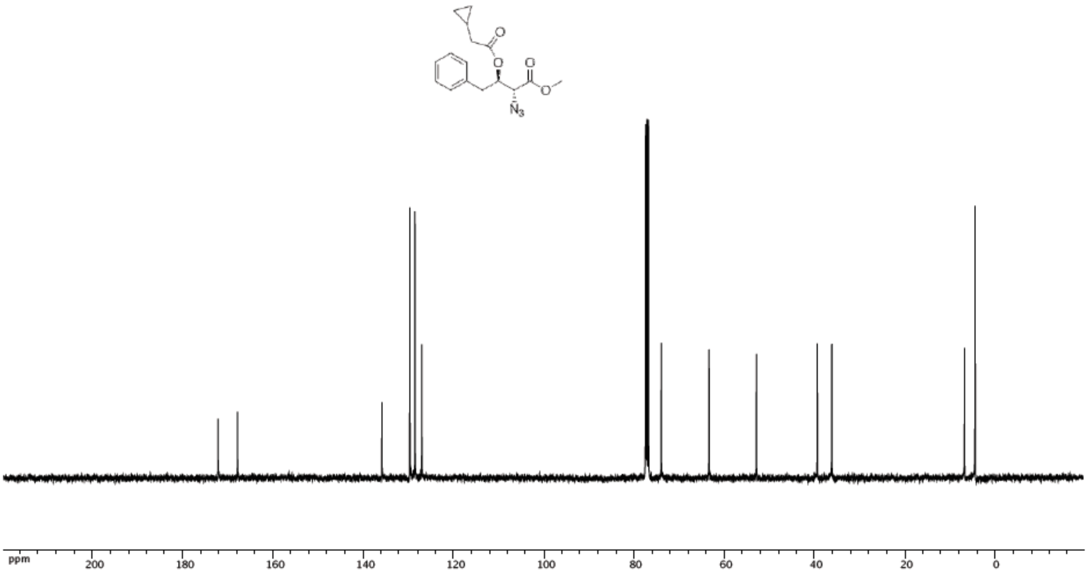

^1H- and ^{13}C-NMR of compound (±)-**15d** in $CDCl_3$

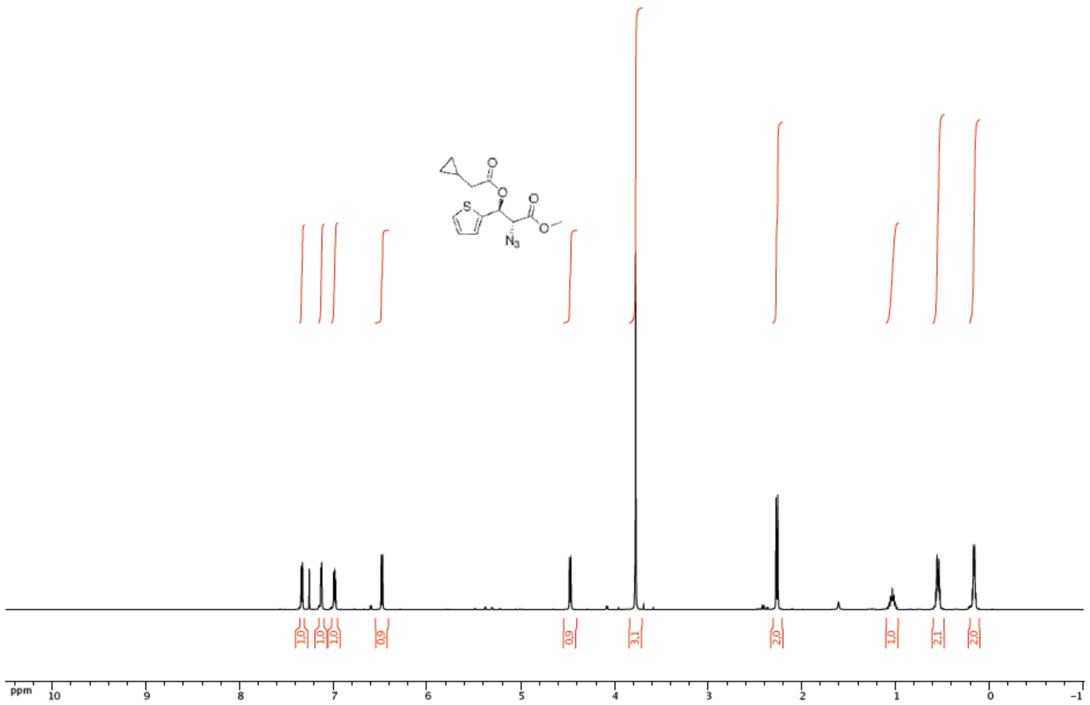

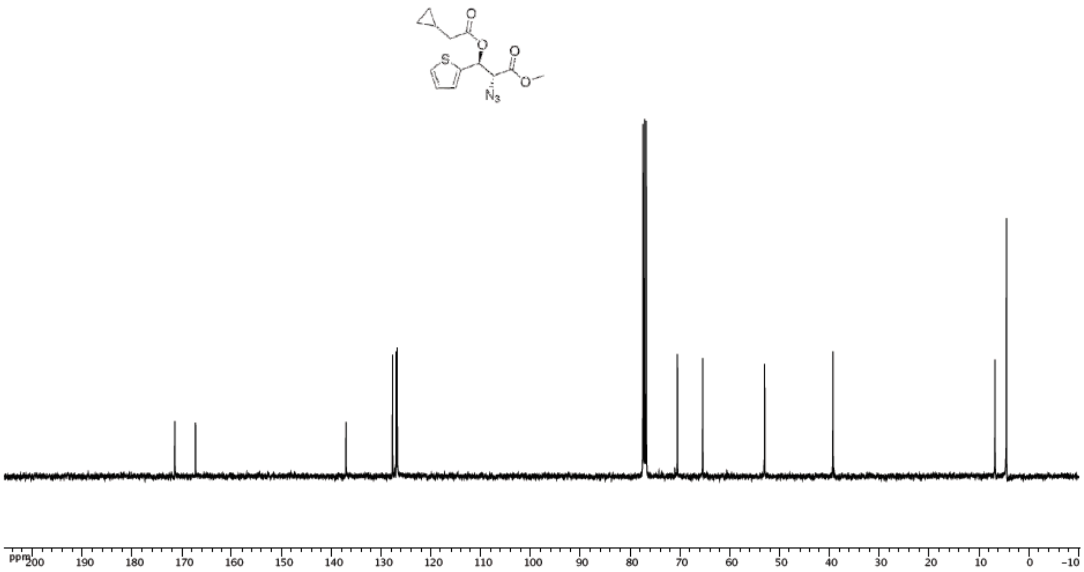

^{1}H- and ^{13}C-NMR of compound (±)-**16a** in $CDCl_3$

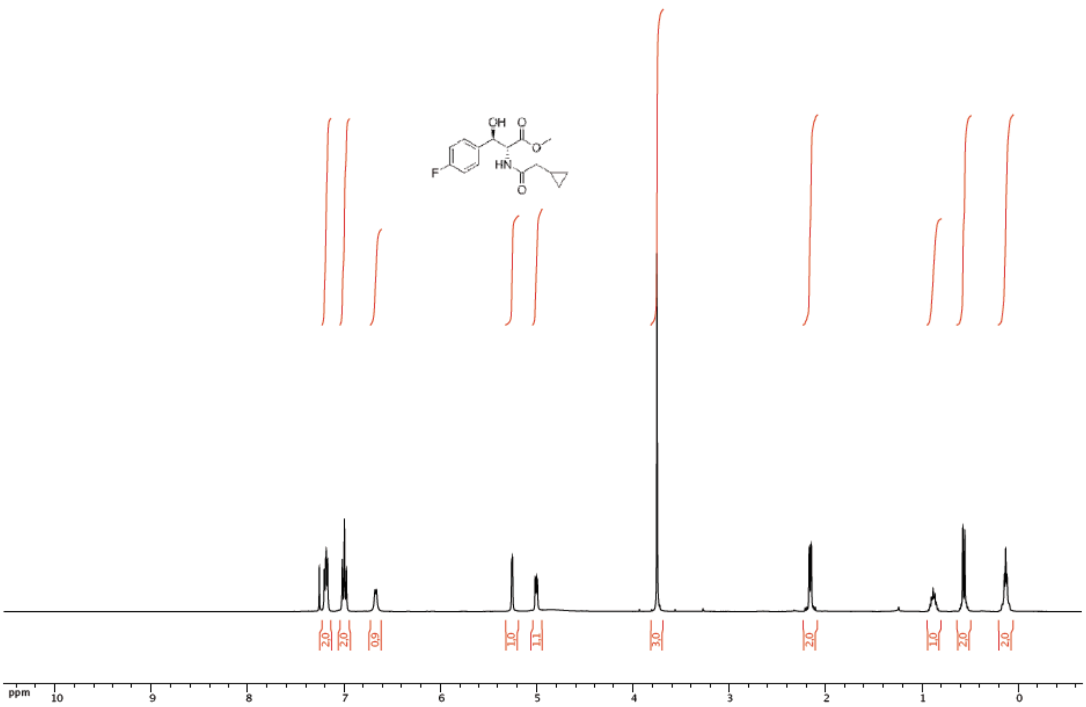

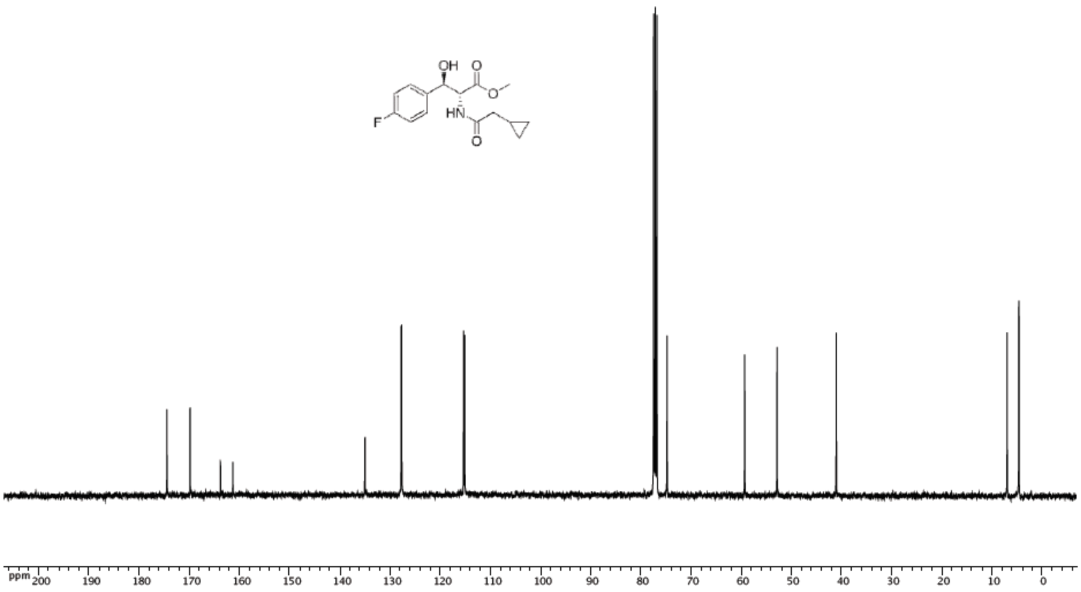

^{1}H- and ^{13}C-NMR of compound (±)-**16b** in $CDCl_3$

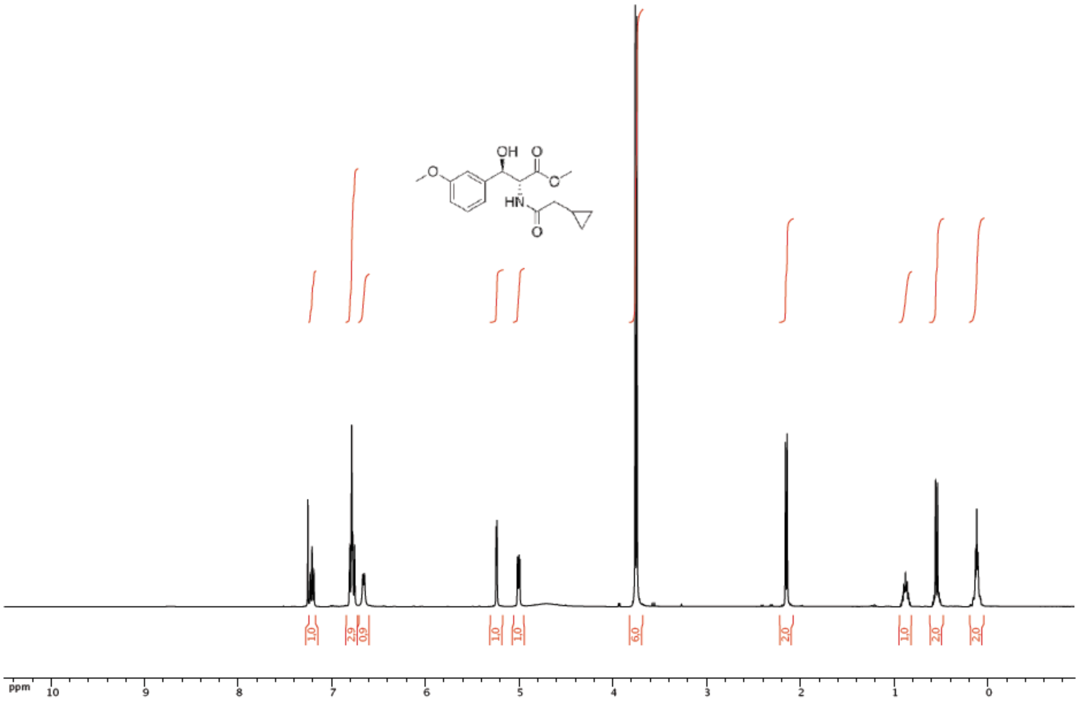

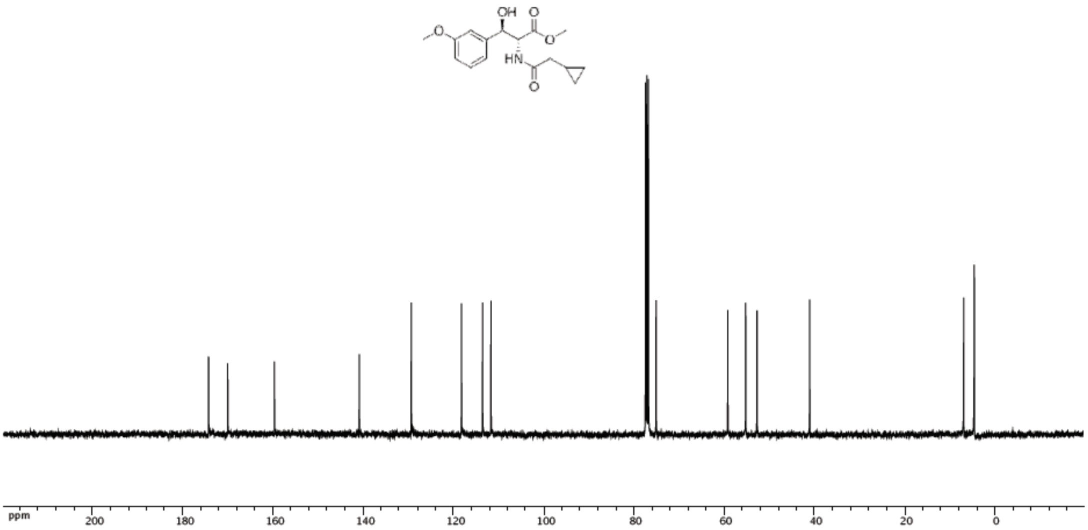

^{1}H- and ^{13}C-NMR of compound (±)-**16c** in $CDCl_3$

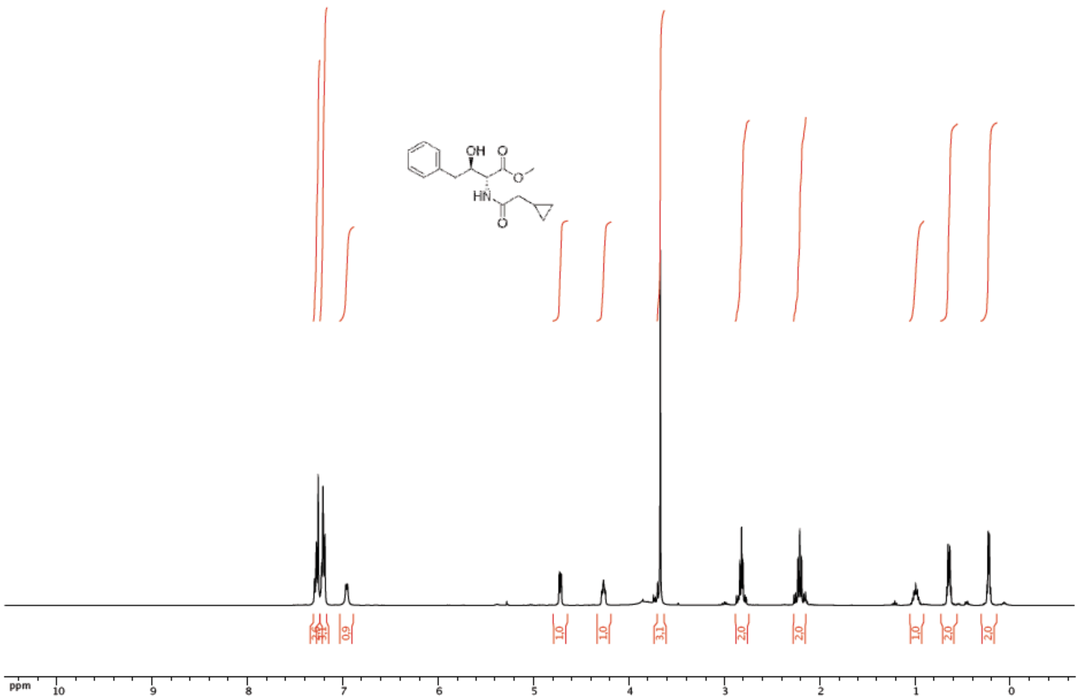

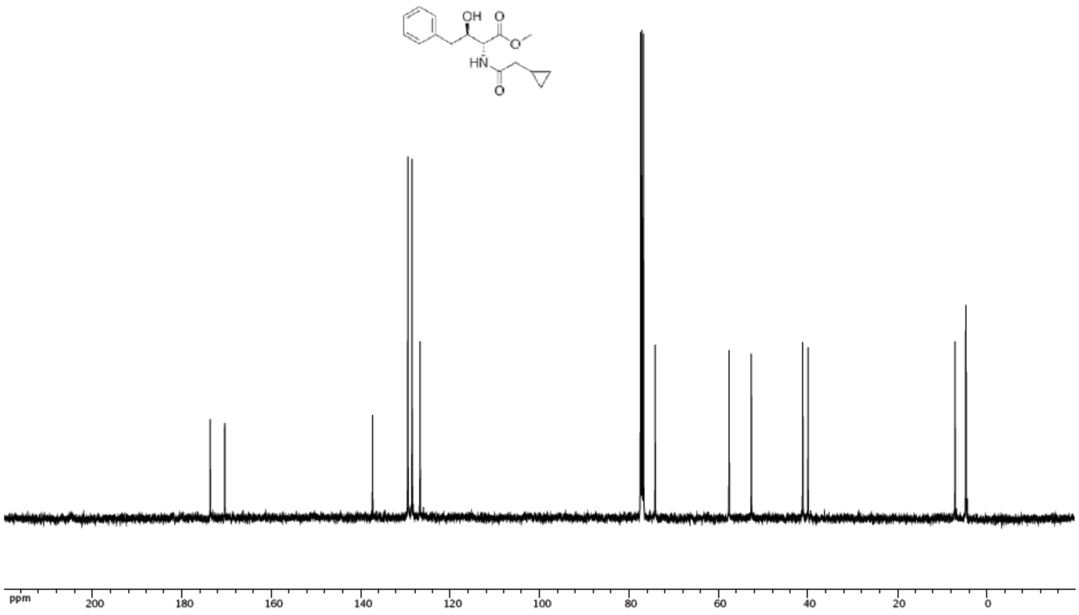

^{1}H- and ^{13}C-NMR of compound (±)-**16d** in $CDCl_3$

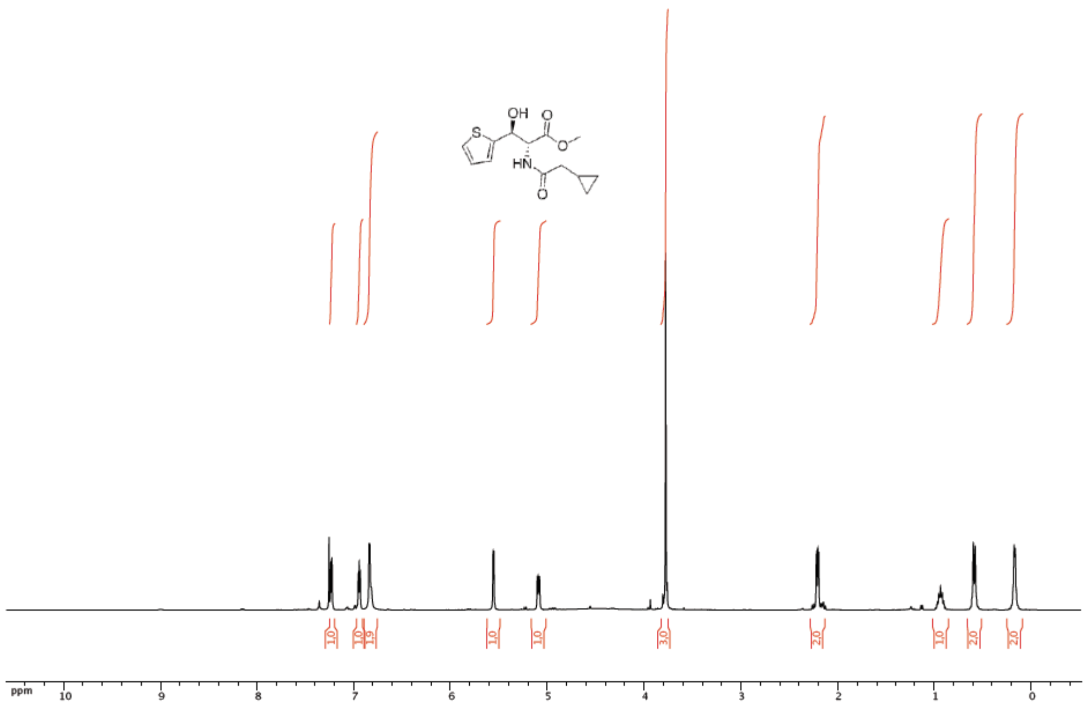

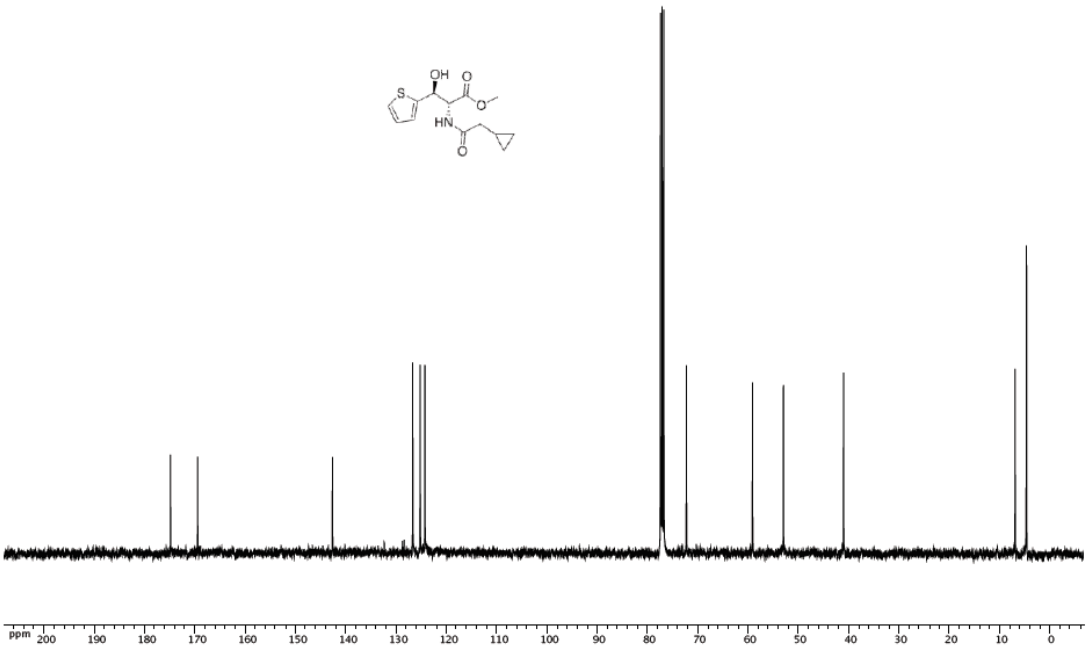

^{1}H- and ^{13}C-NMR of compound (±)-**17a** in $CDCl_3$

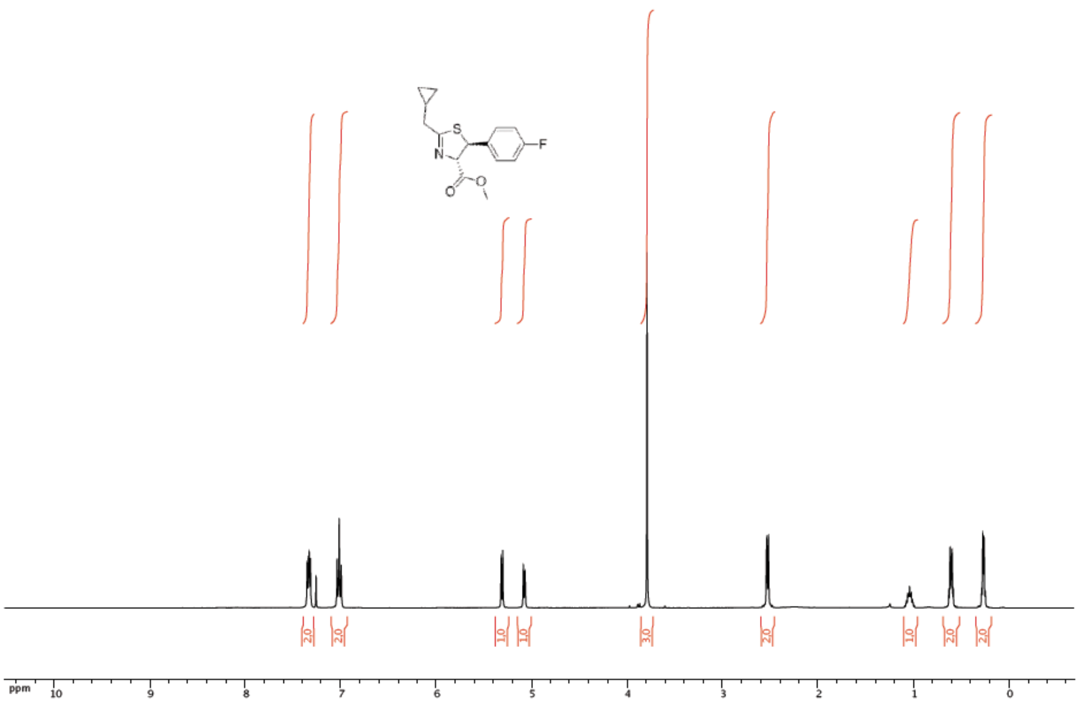

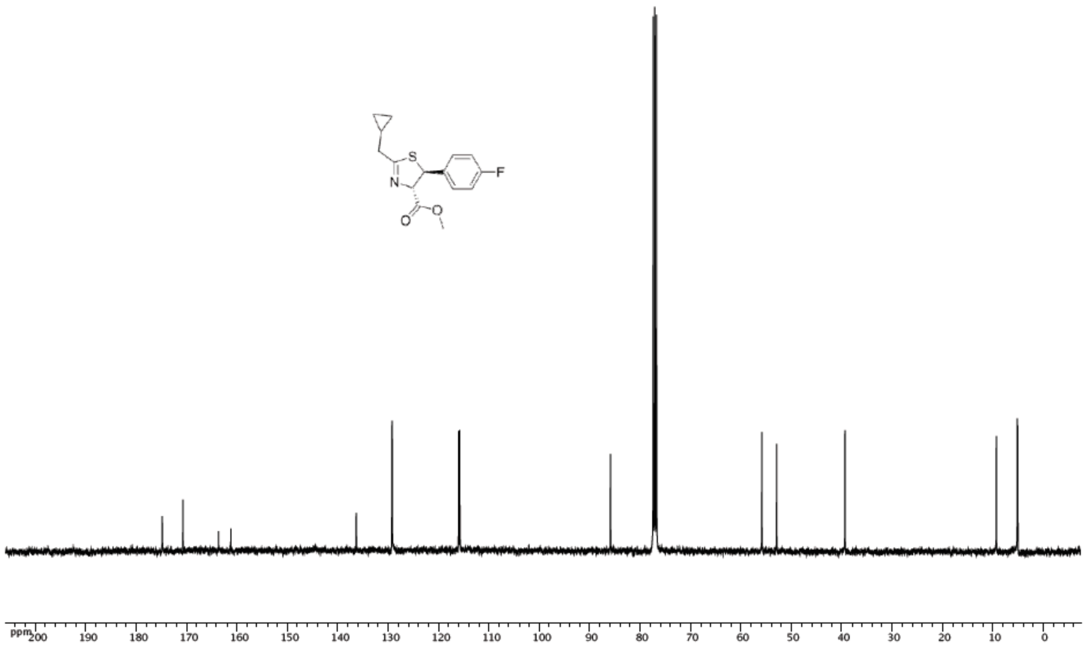

^{1}H- and ^{13}C-NMR of compound (±)-**17b** in $CDCl_3$

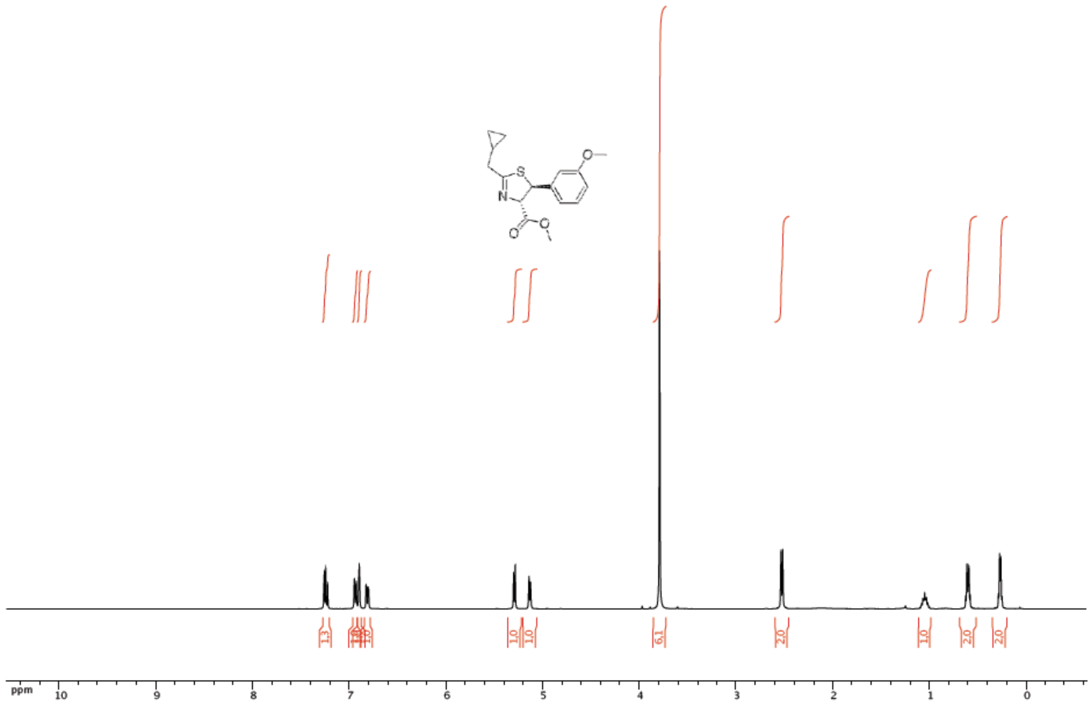

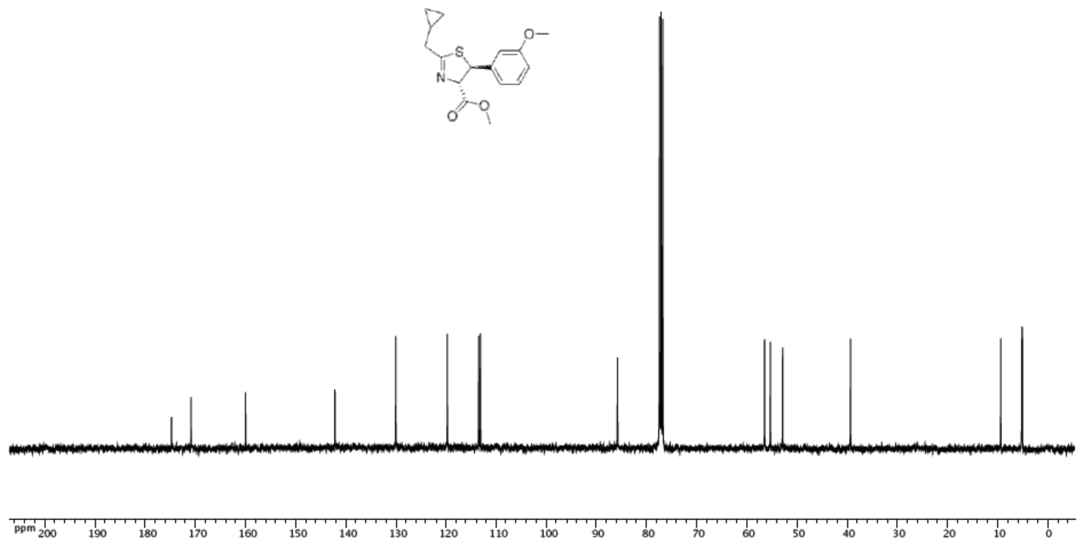

^{1}H- and ^{13}C-NMR of compound (±)-**17c** in $CDCl_3$

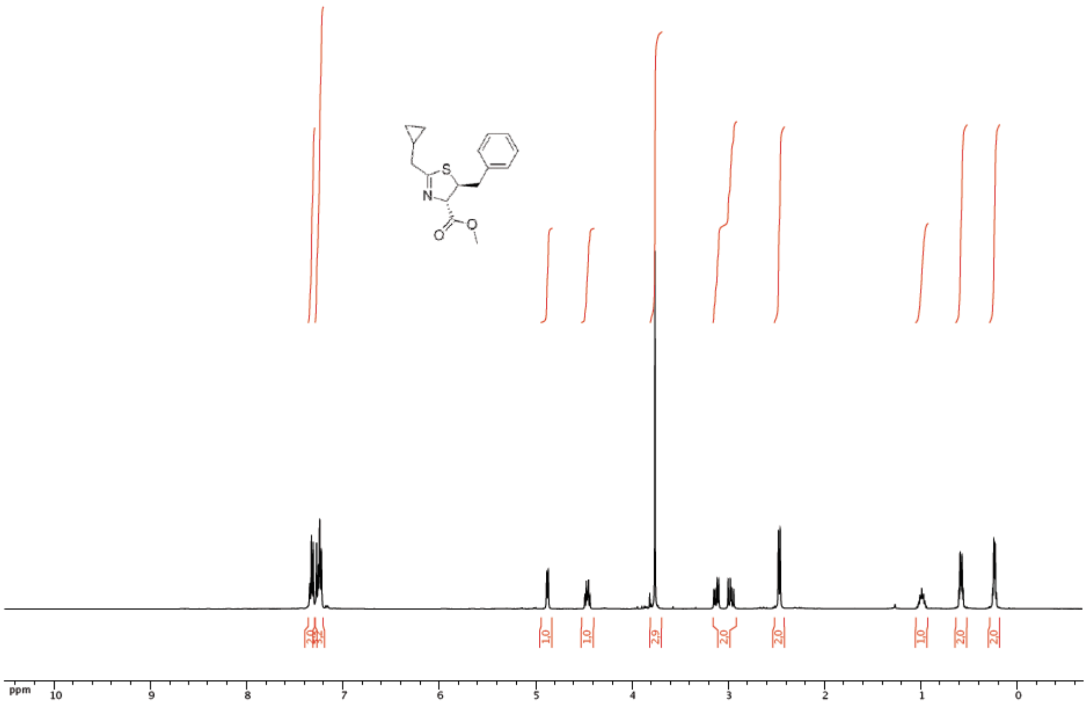

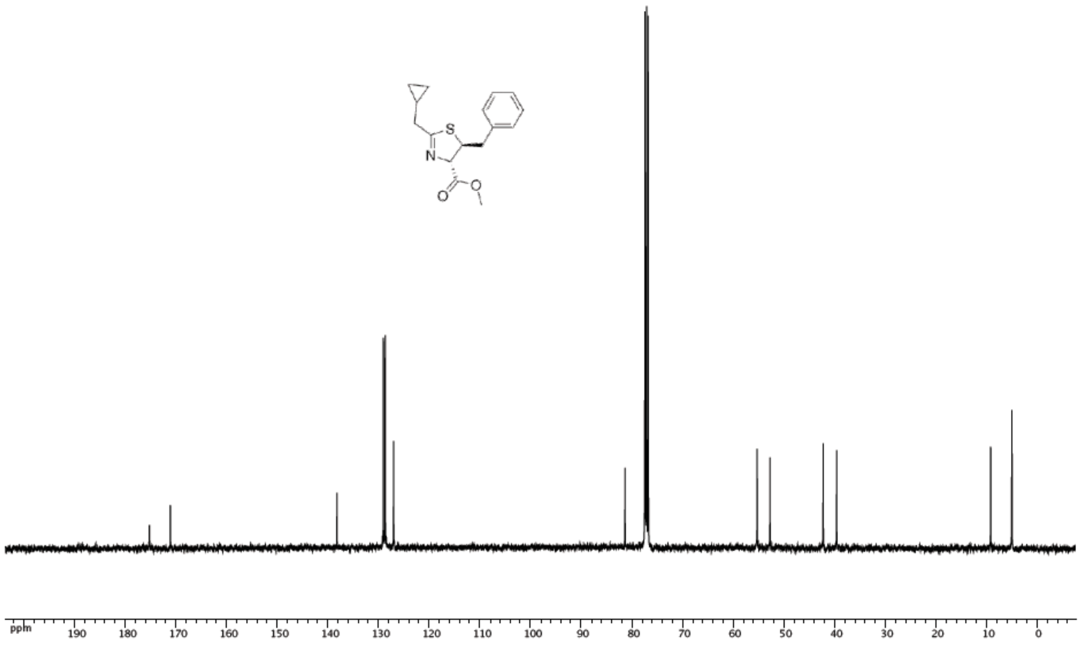

^{1}H- and ^{13}C-NMR of compound (±)-**17d** in $CDCl_3$

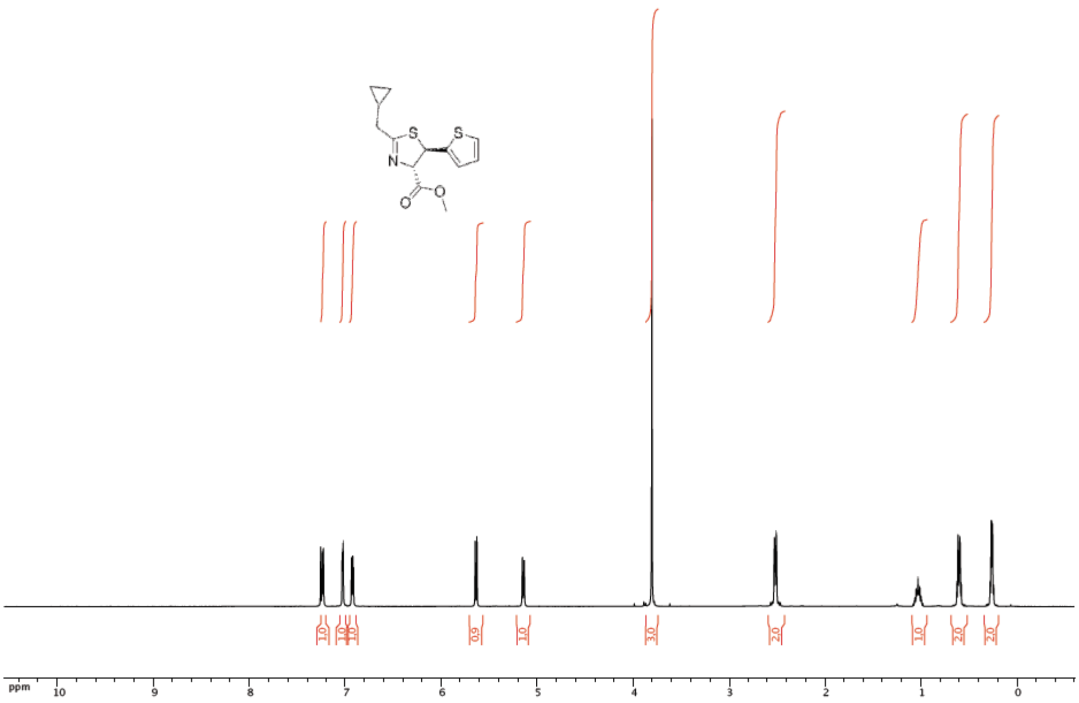

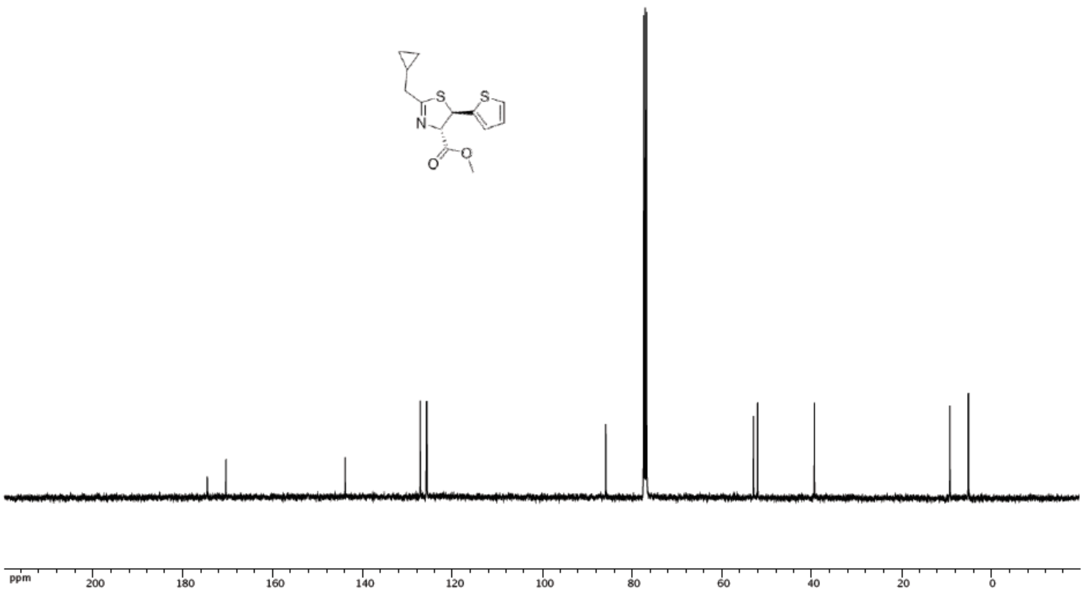

^{1}H- and ^{13}C-NMR of compound (-)-**18b** in $CDCl_3$

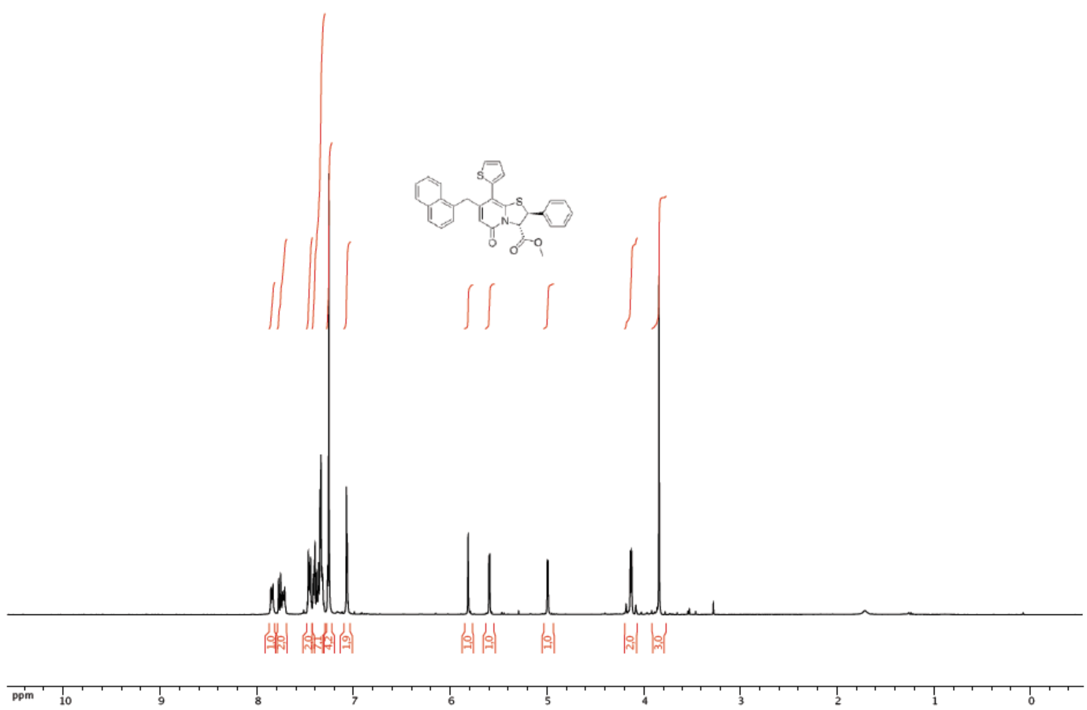

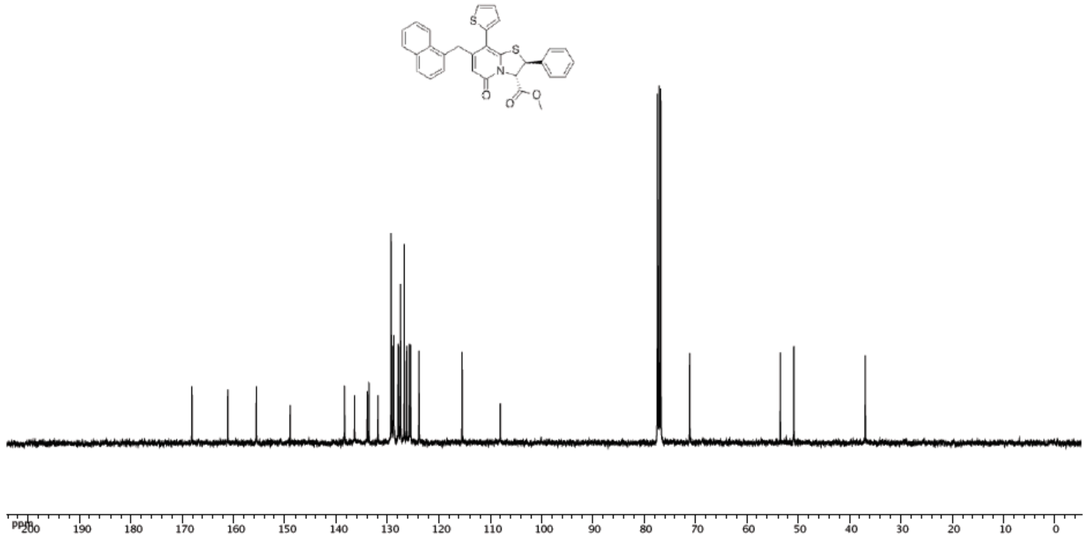

Chiral chromatogram of compound (±)-**8b**
Column: (S, S) Whelk-O1, 25cm x 4.6mm
Eluent: Hexane:DCM:*t*BuOH 76:20:4
Flow rate: 1ml/min
UV detection at 254nm

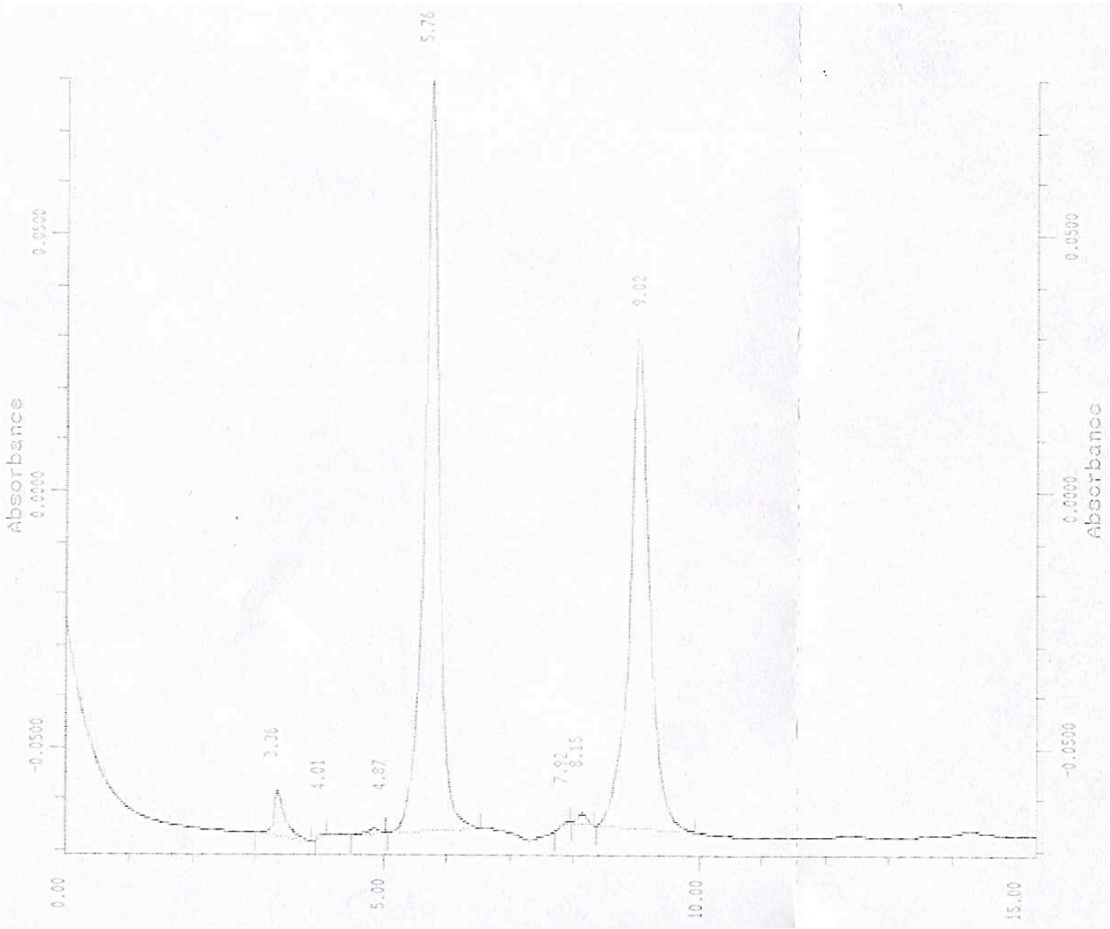

Chiral chromatogram of compound (-)-**8b** ee 99%
Column: (S, S) Whelk-O1, 25cm x 4.6mm
Eluent: Hexane:DCM:*t*BuOH 76:20:4
Flow rate: 1ml/min
UV detection at 254nm

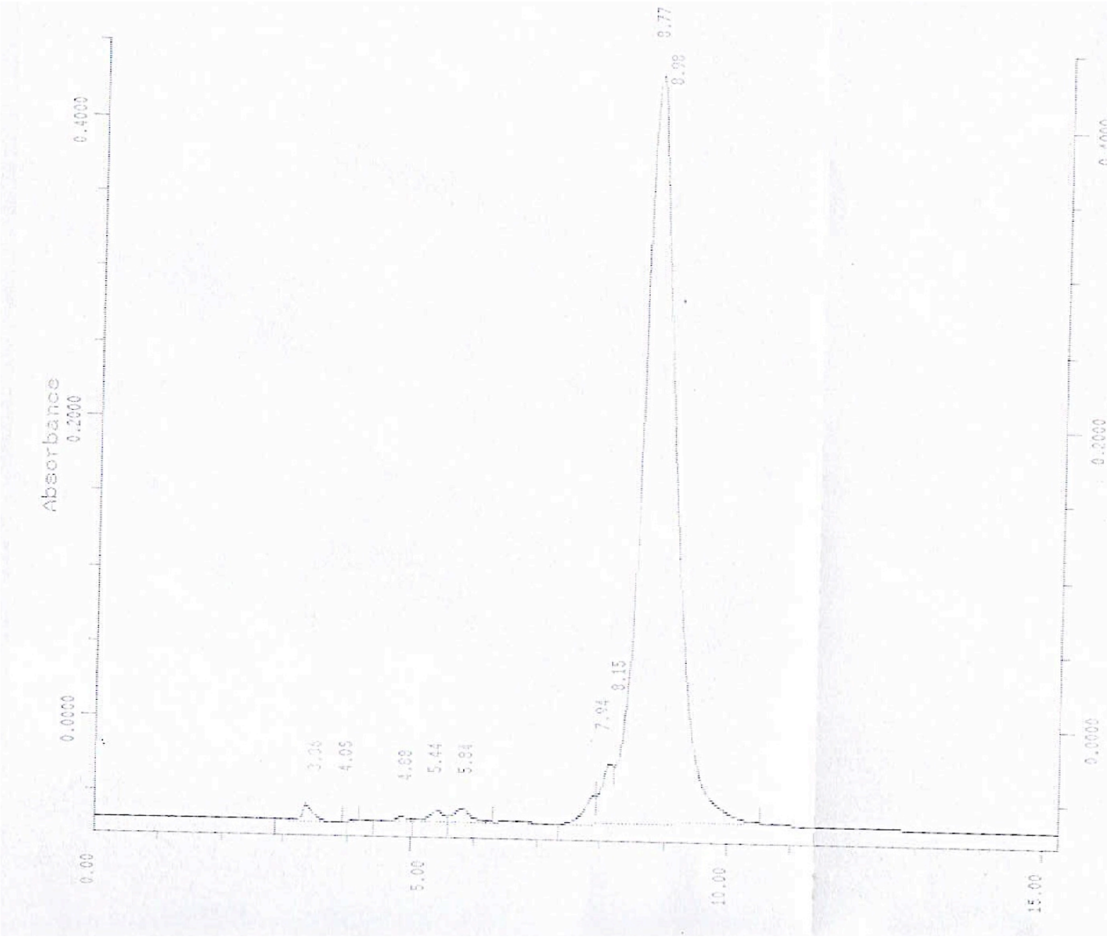

Chiral chromatogram of compound (+)-**8b** ee 99%
Column: (S, S) Whelk-O1, 25cm x 4.6mm
Eluent: Hexane:DCM:*t*BuOH 76:20:4
Flow rate: 1ml/min
UV detection at 254nm

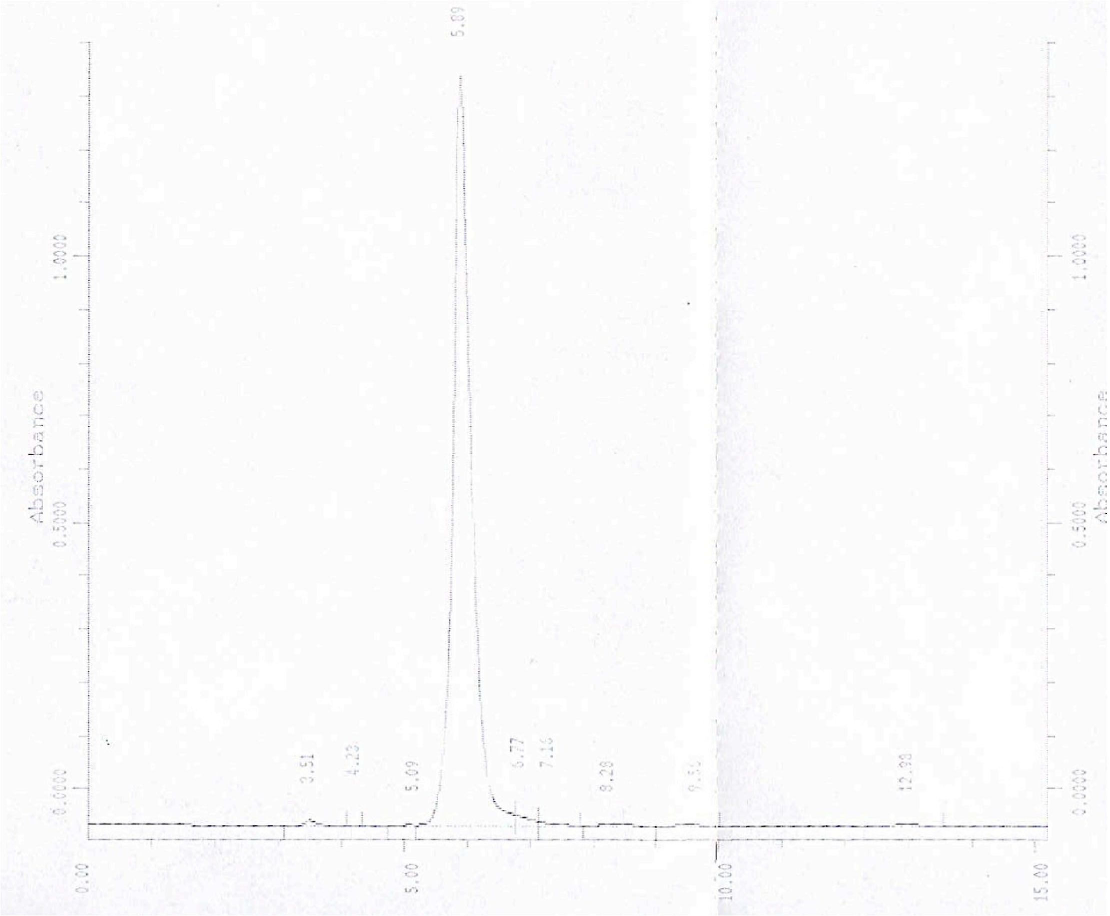

Chiral chromatogram of compound (±)-**8e**
Column: (S, S) Whelk-O1, 25cm x 4.6mm
Eluent: Hexane:DCM:*t*BuOH 76:20:4
Flow rate: 1ml/min
UV detection at 254nm

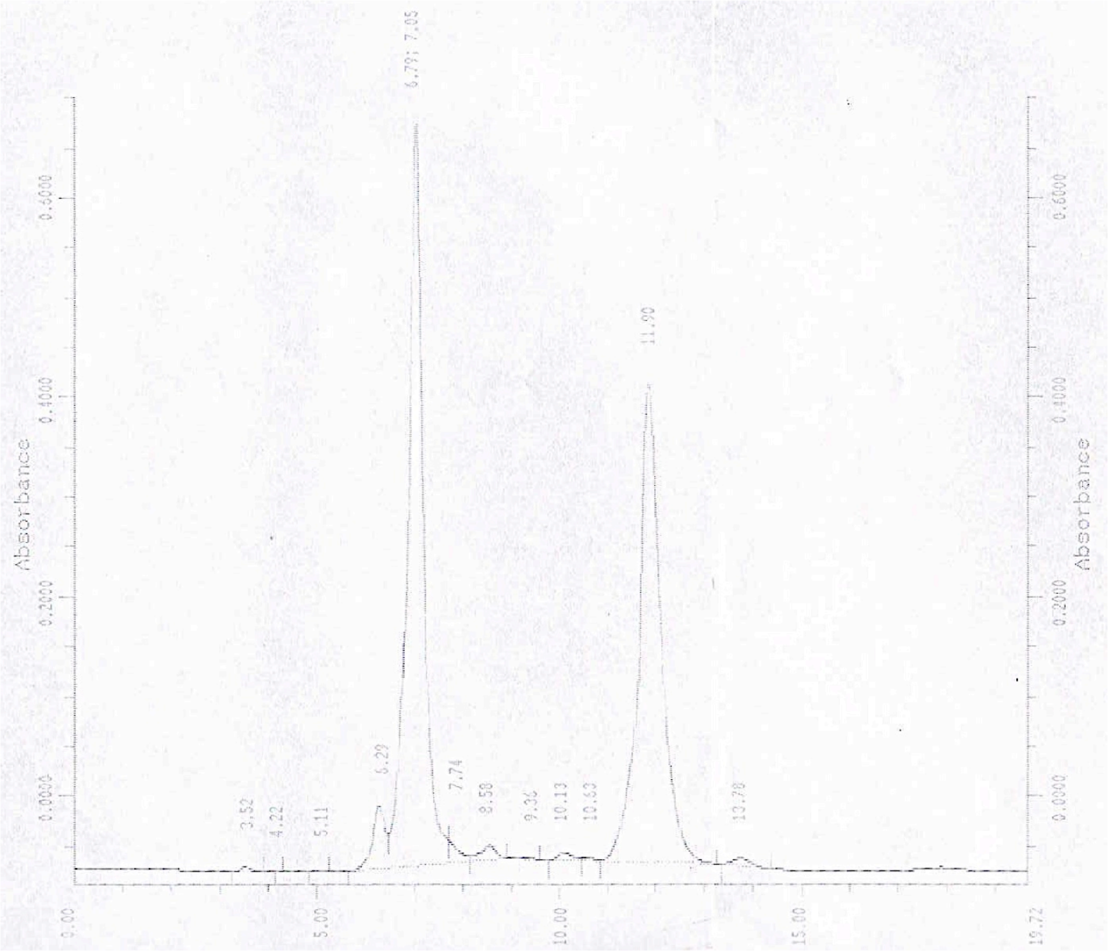

Chiral chromatogram of compound (-)-**8e** ee 99%
Column: (S, S) Whelk-O1, 25cm x 4.6mm
Eluent: Hexane:DCM:*t*BuOH 76:20:4
Flow rate: 1ml/min
UV detection at 254nm

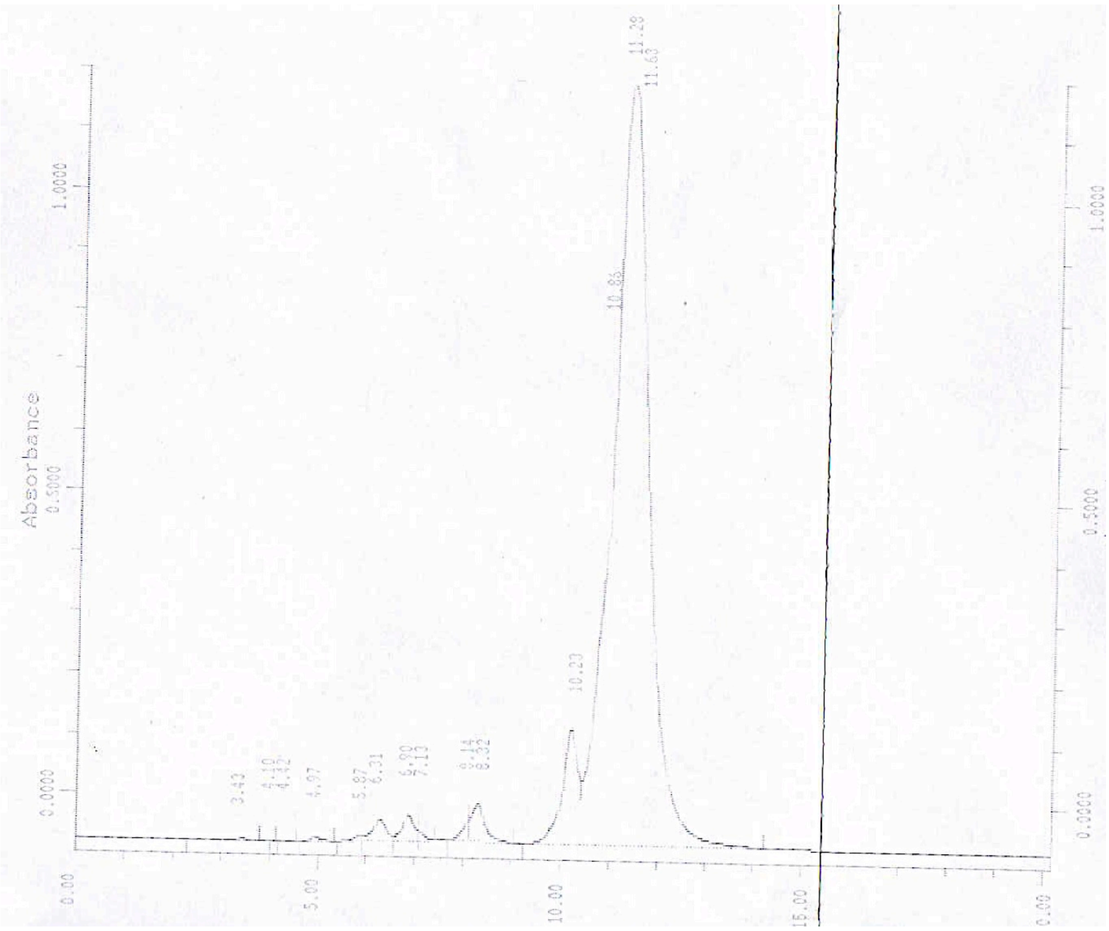

Chiral chromatogram of compound (+)-**8e** ee 99%
Column: (S, S) Whelk-O1, 25cm x 4.6mm
Eluent: Hexane:DCM:*t*BuOH 76:20:4
Flow rate: 1ml/min
UV detection at 254nm

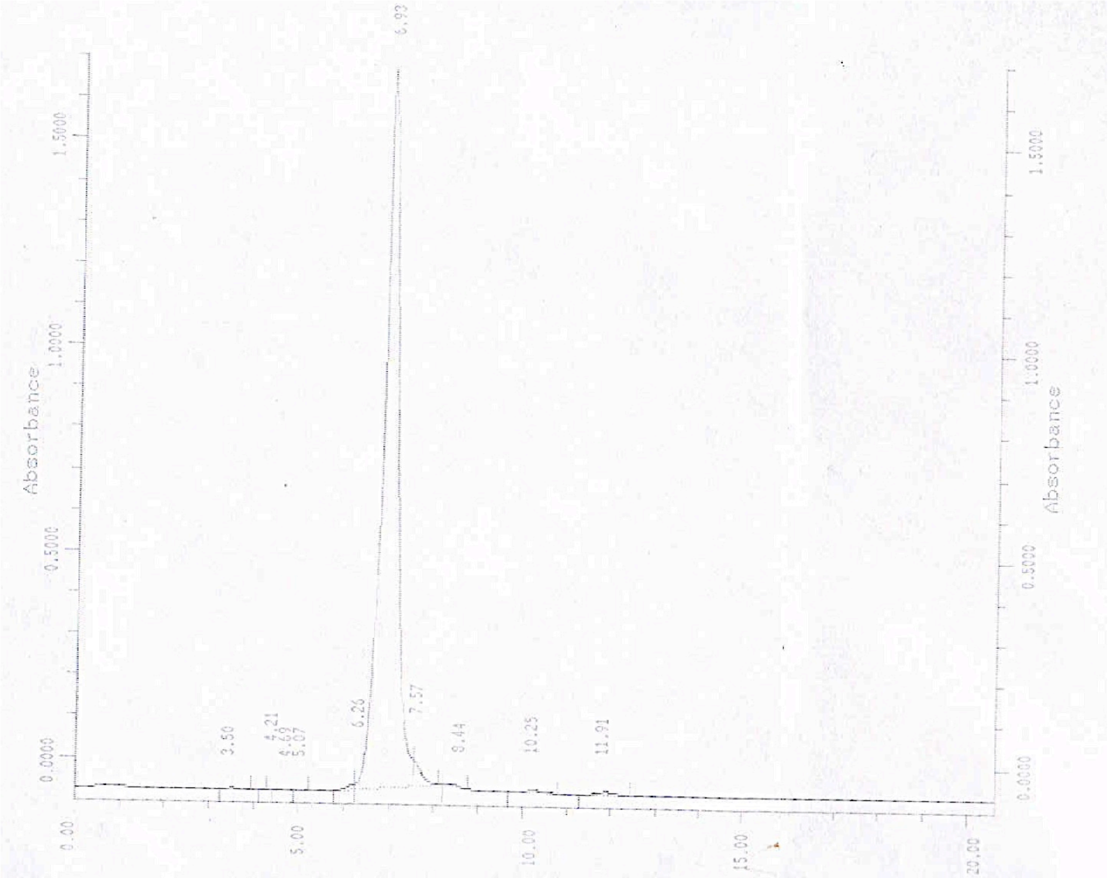

SFC chromatogram of compound (±)-**9b**
Column: Chiralpak AD, 25cm x 4.6mm, 5µm
Eluent: CO_2:MeOH 80:20, 120 bar
Temperature: 40 °C
Flow rate: 4ml/min
UV detection at 220nm

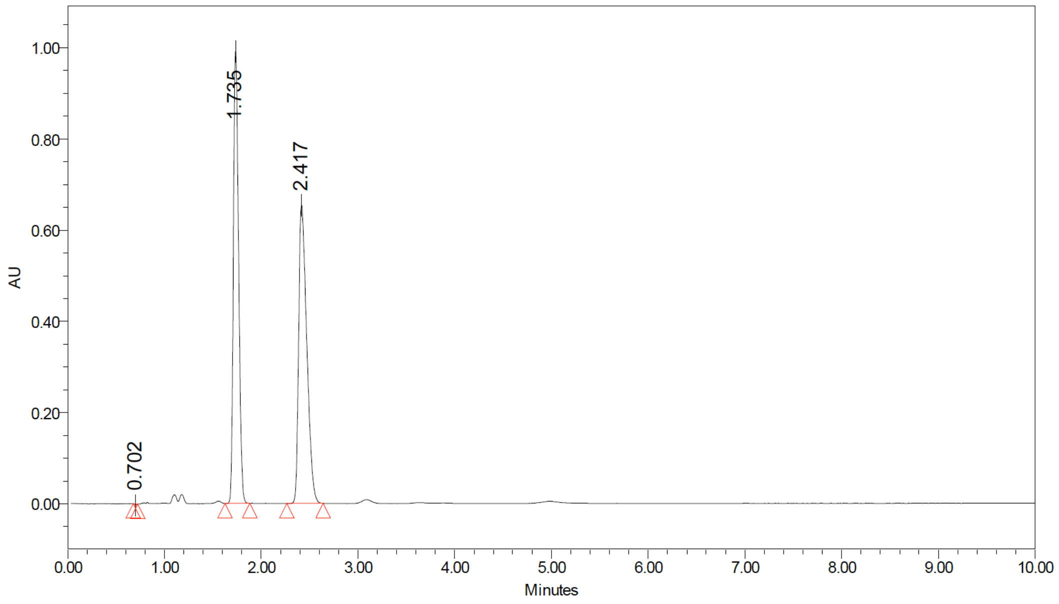

SFC chromatogram of compound (+)-**9b** ee 99%
Column: Chiralpak AD, 25cm x 4.6mm, 5μm
Eluent: CO_2:MeOH 80:20, 120 bar
Temperature: 40 °C
Flow rate: 4ml/min
UV detection at 220nm

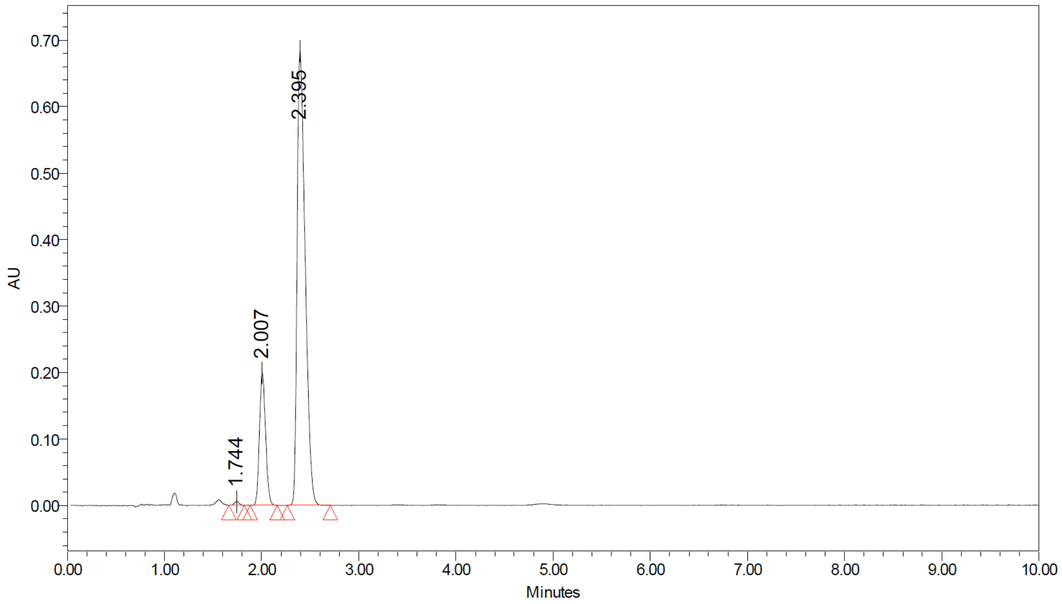

SFC chromatogram of compound (-)-**9b** ee 99%
Column: Chiralpak AD, 25cm x 4.6mm, 5µm
Eluent: CO_2:MeOH 80:20, 120 bar
Temperature: 40 ^{o}C
Flow rate: 4ml/min
UV detection at 220nm

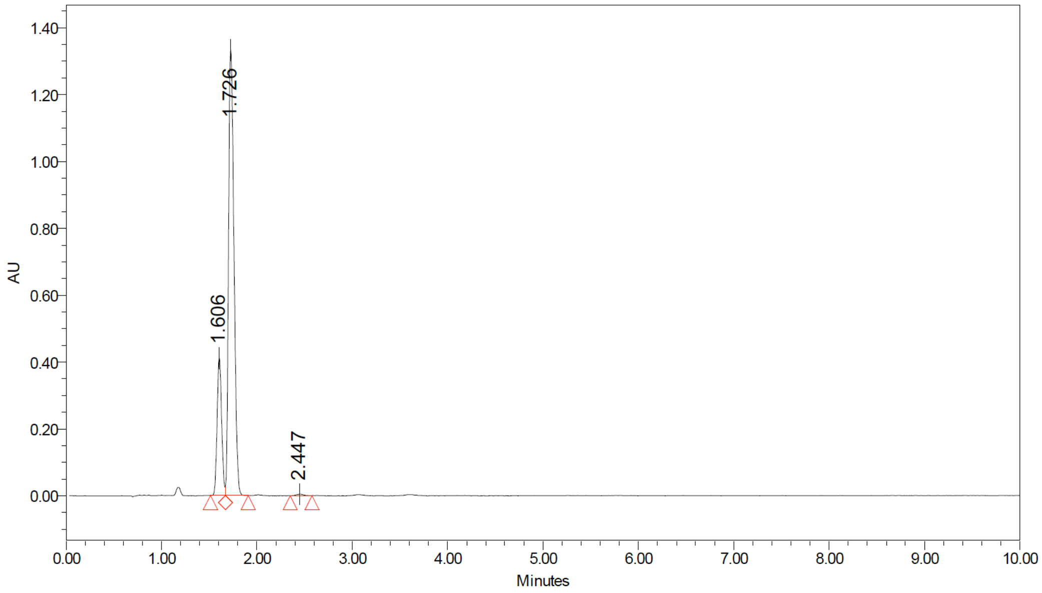

SFC chromatogram of compound (±)-**9e**
Column: Lux C4, 25cm x 4.6mm, 5µm
Eluent: CO_2:MeOH 85:15, 120 bar
Temperature: 40 °C
Flow rate: 1ml/min
UV detection at 220nm

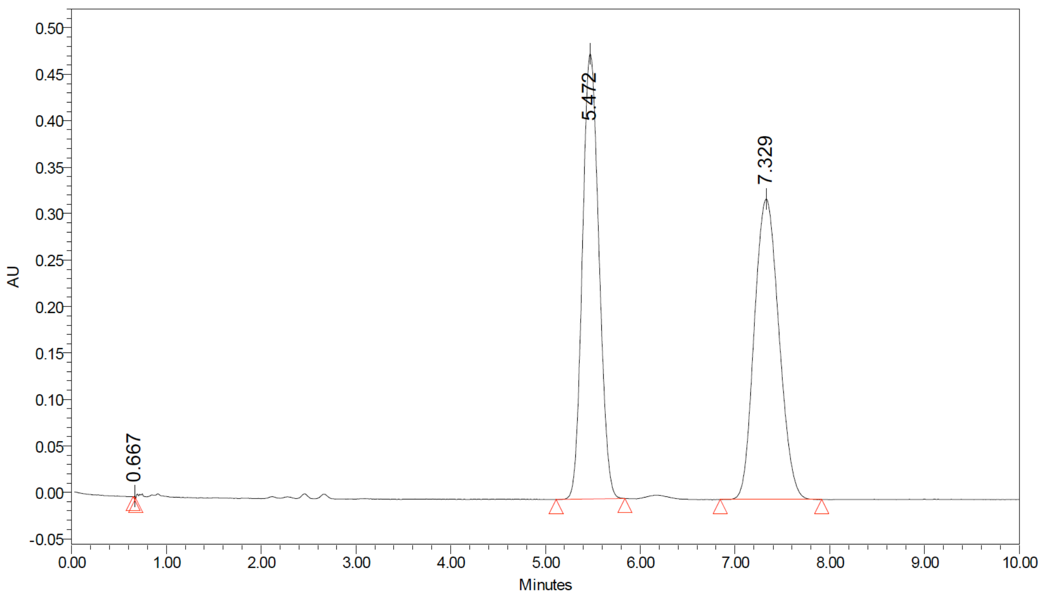

SFC chromatogram of compound (+)-**9e** ee 99%
Column: Lux C4, 25cm x 4.6mm, 5µm
Eluent: CO_2:MeOH 85:15, 120 bar
Temperature: 40 °C
Flow rate: 1ml/min
UV detection at 220nm

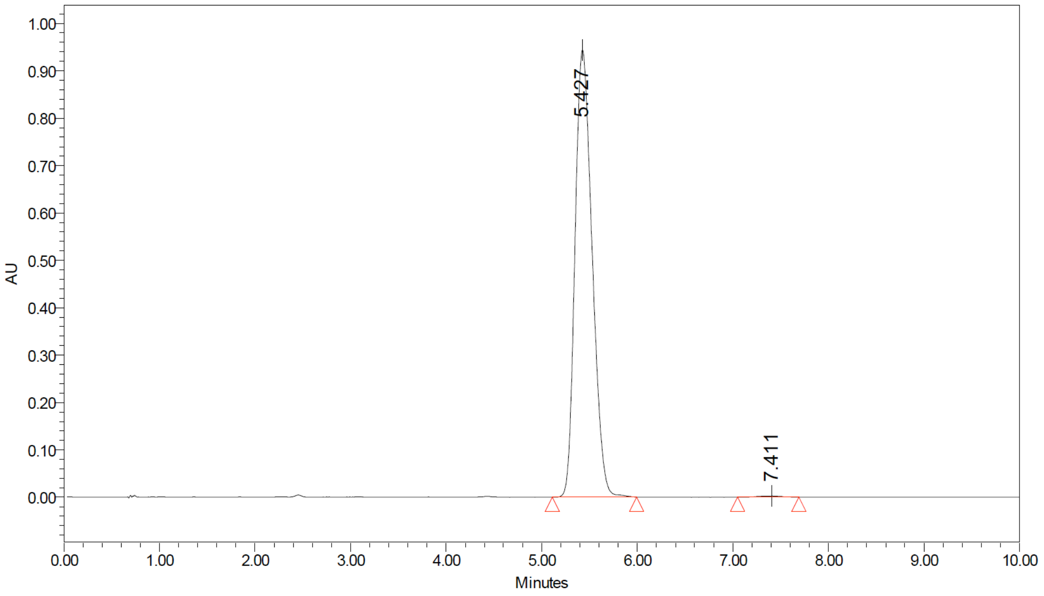

SFC chromatogram of compound (-)-**9e** ee 99%
Column: Lux C4, 25cm x 4.6mm, 5µm
Eluent: CO_2:MeOH 85:15, 120 bar
Temperature: 40 °C
Flow rate: 1ml/min
UV detection at 220nm

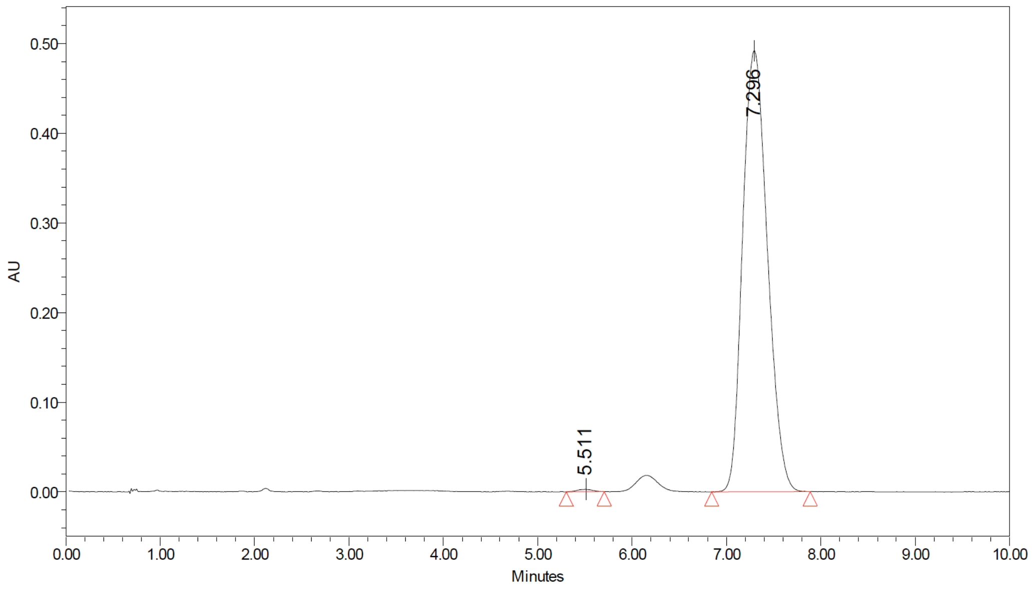

Chiral chromatogram of compound (±)-**10a**
Column: (S, S) Whelk-O1, 25cm x 4.6mm
Eluent: Hexane:DCM:*t*BuOH 76:20:4
Flow rate: 1ml/min
UV detection at 254nm

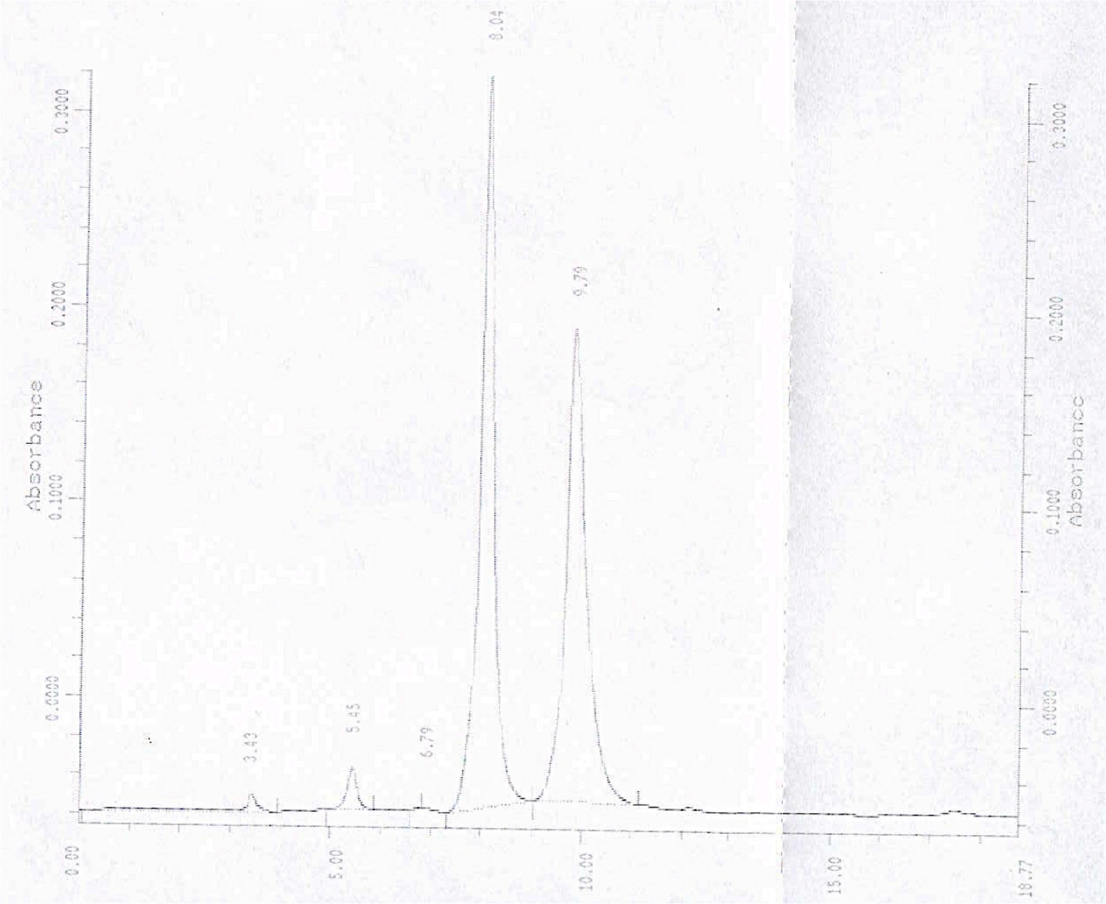

Chiral chromatogram of compound (-)-**10a** ee 80%
Column: (S, S) Whelk-O1, 25cm x 4.6mm
Eluent: Hexane:DCM:*t*BuOH 76:20:4
Flow rate: 1ml/min
UV detection at 254nm

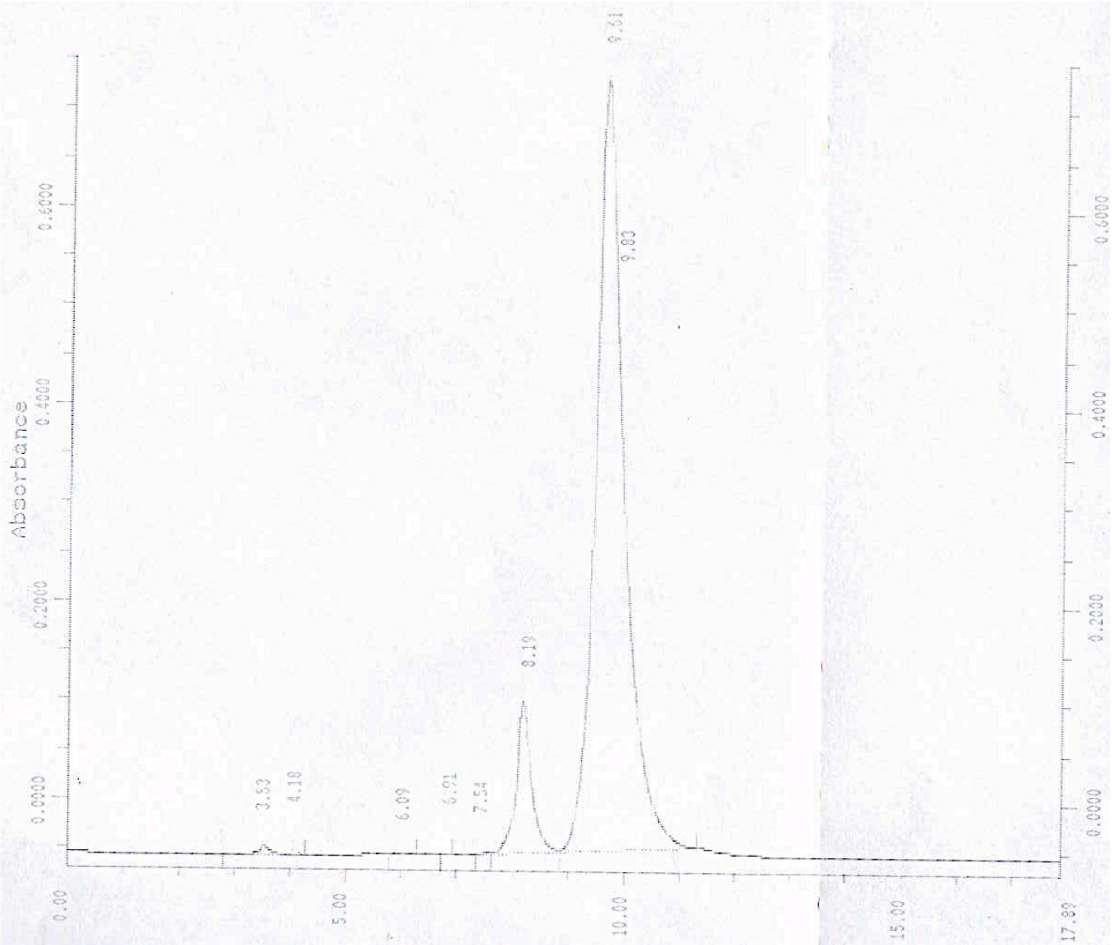

Chiral chromatogram of compound (+)-**10a** ee 71%
Column: (S, S) Whelk-O1, 25cm x 4.6mm
Eluent: Hexane:DCM:*t*BuOH 76:20:4
Flow rate: 1ml/min
UV detection at 254nm

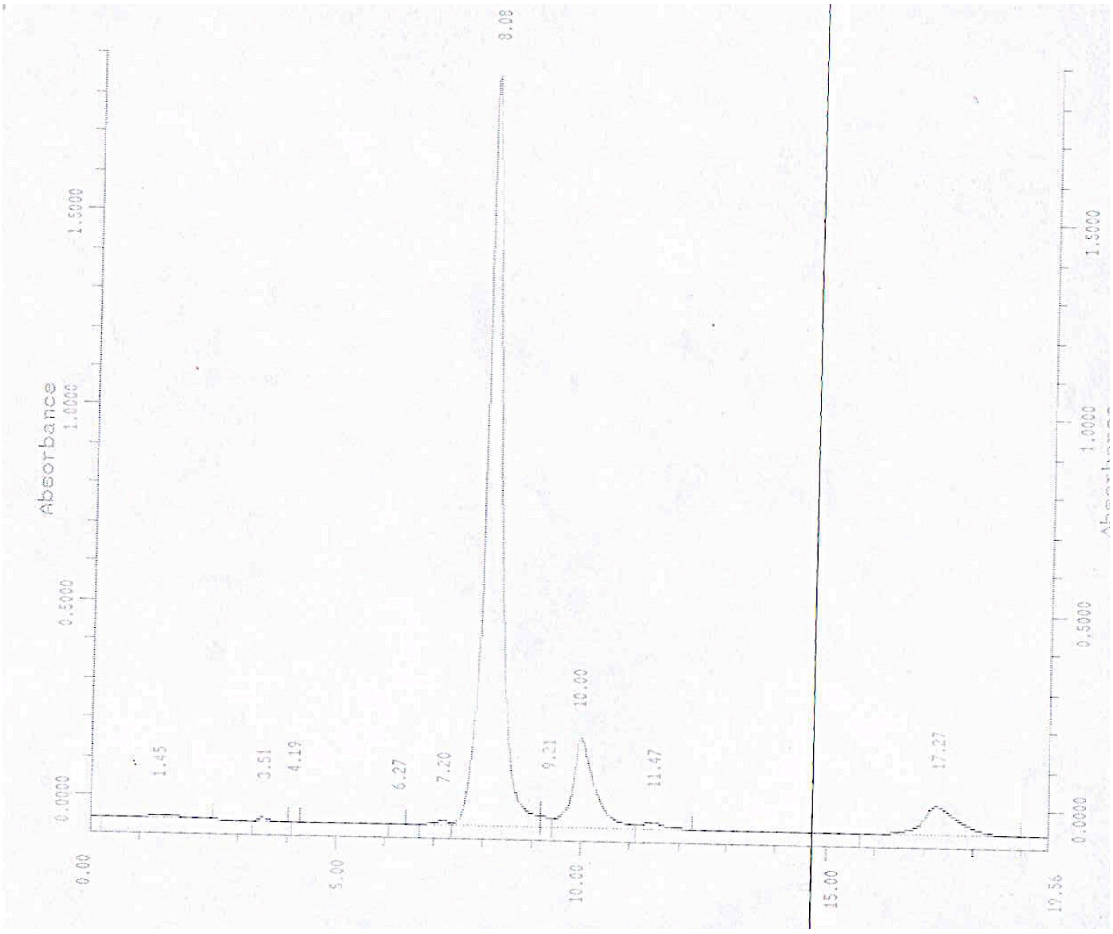

Chiral chromatogram of compound (±)-**10d**
Column: (S, S) Whelk-O1, 25cm x 4.6mm
Eluent: Hexane:DCM:*t*BuOH 76:20:4
Flow rate: 1ml/min
UV detection at 254nm

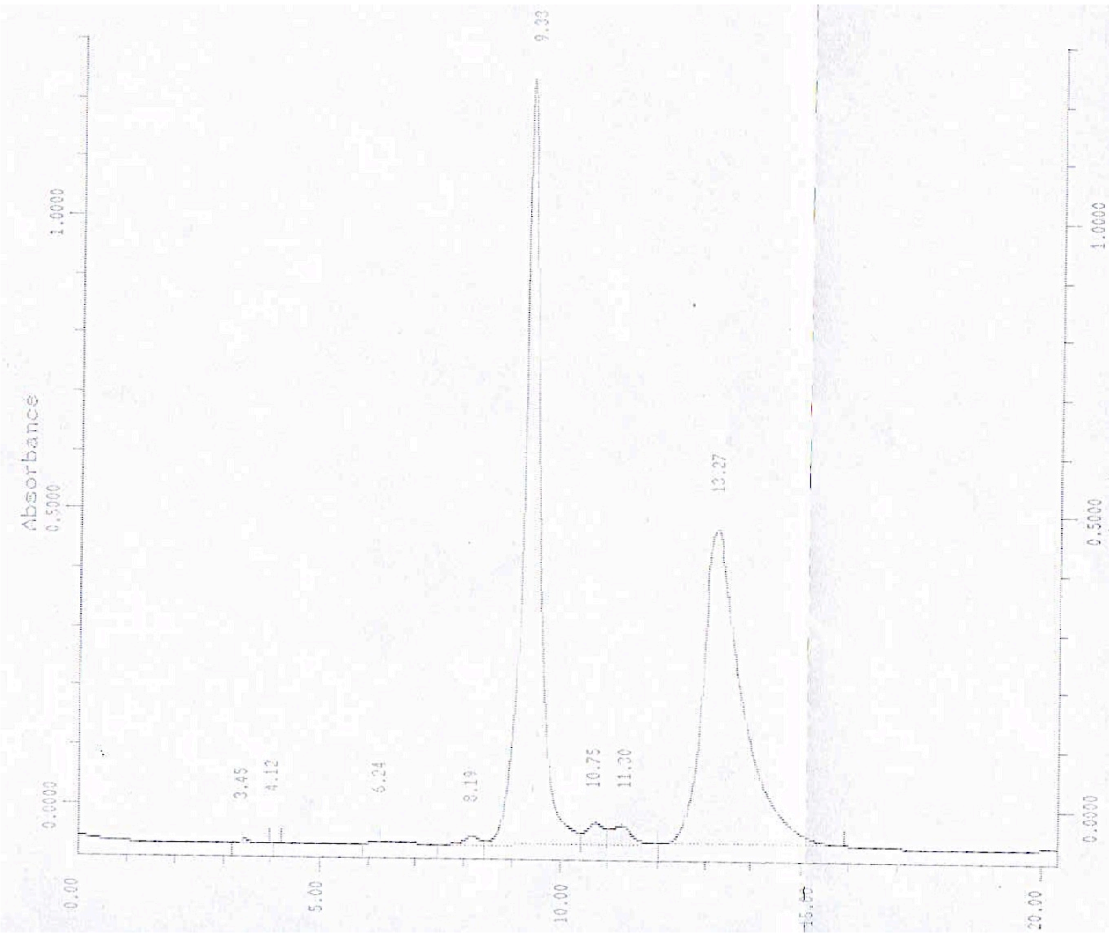

Chiral chromatogram of compound (-)-**10d** ee 88%
Column: (S, S) Whelk-O1, 25cm x 4.6mm
Eluent: Hexane:DCM:*t*BuOH 76:20:4
Flow rate: 1ml/min
UV detection at 254nm

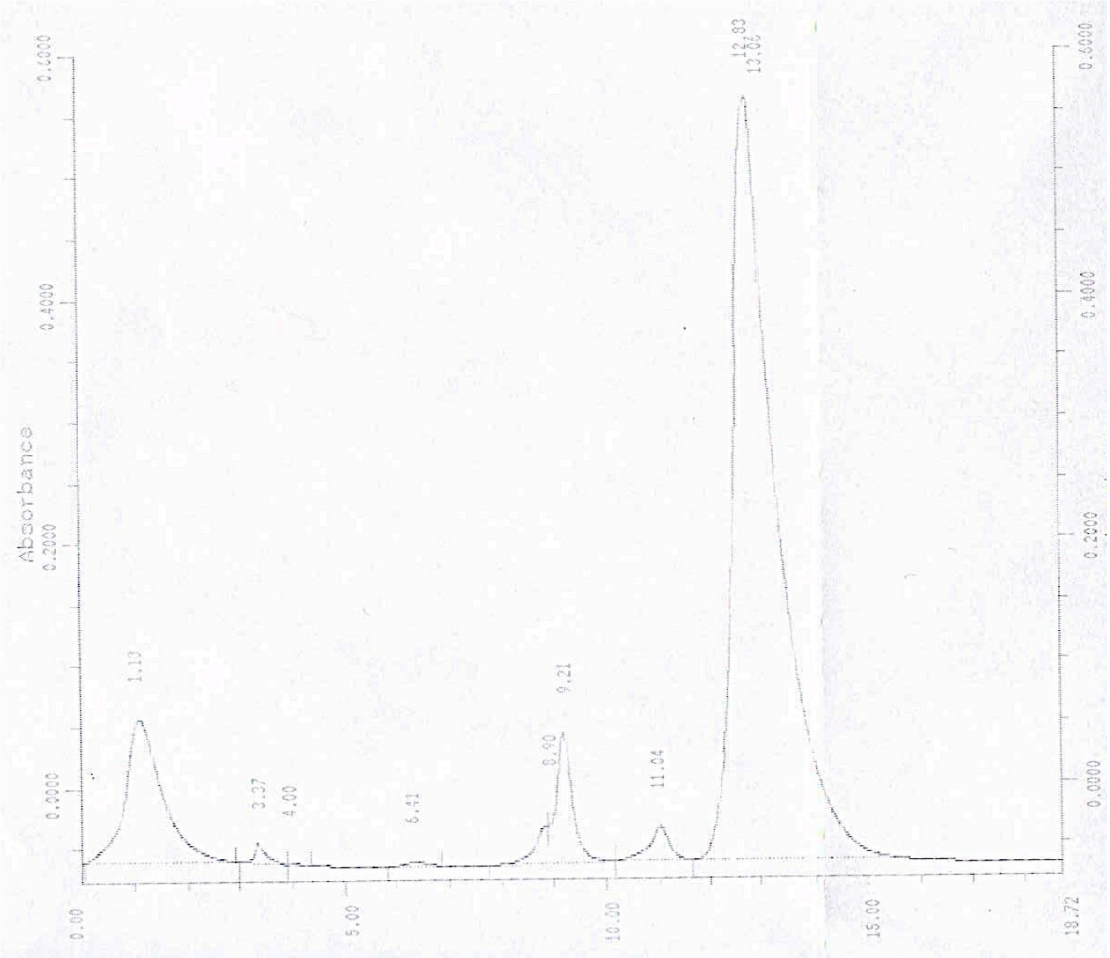

Chiral chromatogram of compound (+)-**10d** ee 83%
Column: (S, S) Whelk-O1, 25cm x 4.6mm
Eluent: Hexane:DCM:*t*BuOH 76:20:4
Flow rate: 1ml/min
UV detection at 254nm

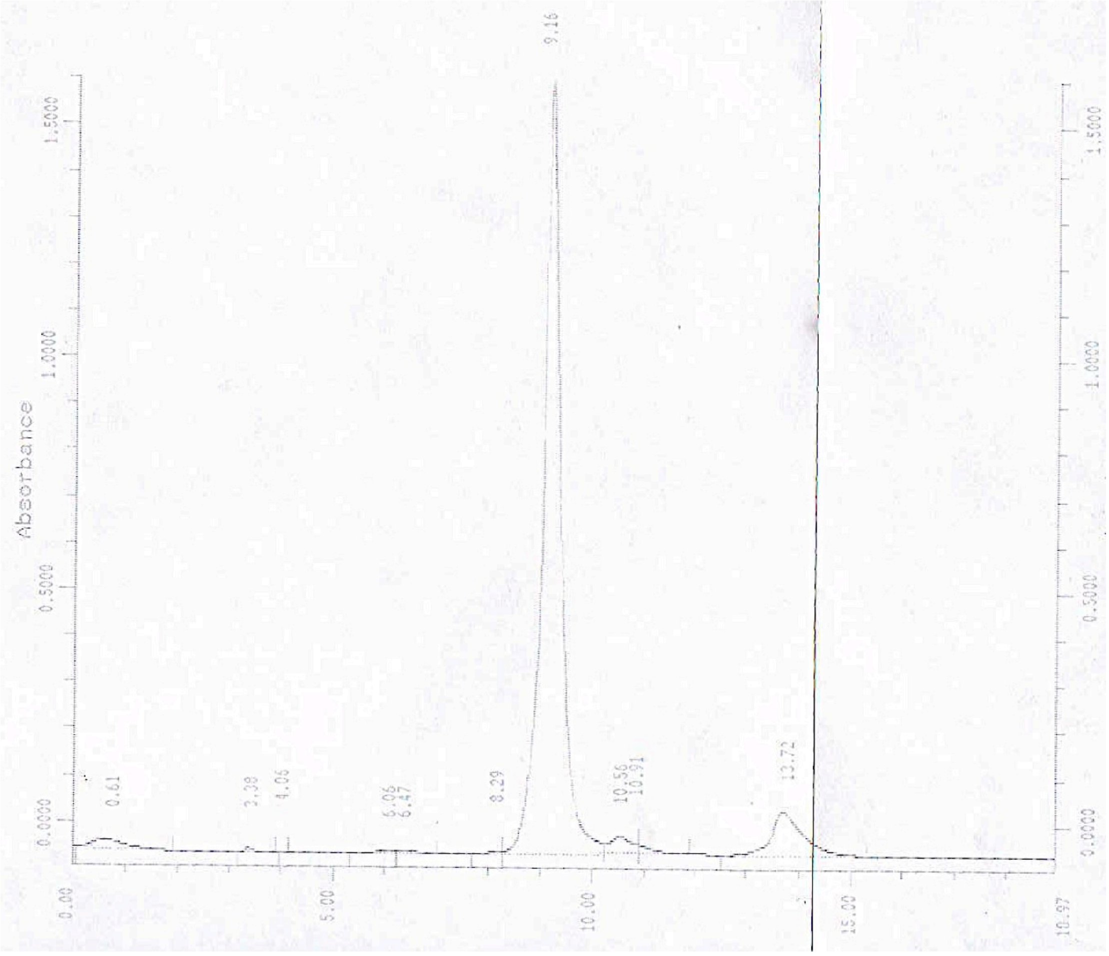

SFC chromatogram of compound (±)-**18a**
Column: Chiralpak AD, 25cm x 4.6mm, 5μm
Eluent: CO_2:MeOH 70:30, 120 bar
Temperature: 40 °C
Flow rate: 4ml/min
UV detection at 220nm

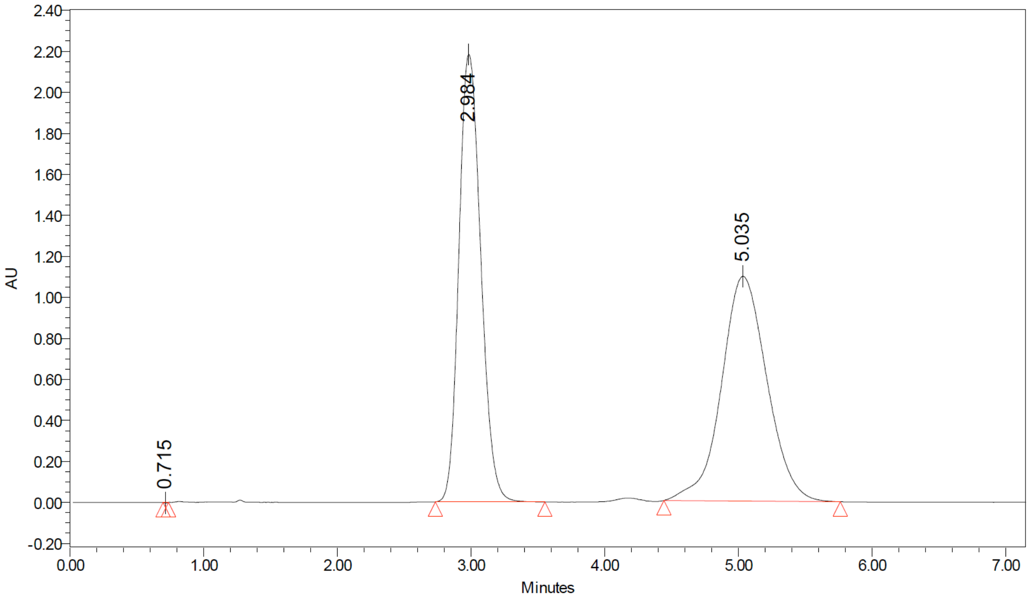

SFC chromatogram of compound (+)-**18a** ee 70%
Column: Chiralpak AD, 25cm x 4.6mm, 5µm
Eluent: CO_2:MeOH 70:30, 120 bar
Temperature: 40 °C
Flow rate: 4ml/min
UV detection at 220nm

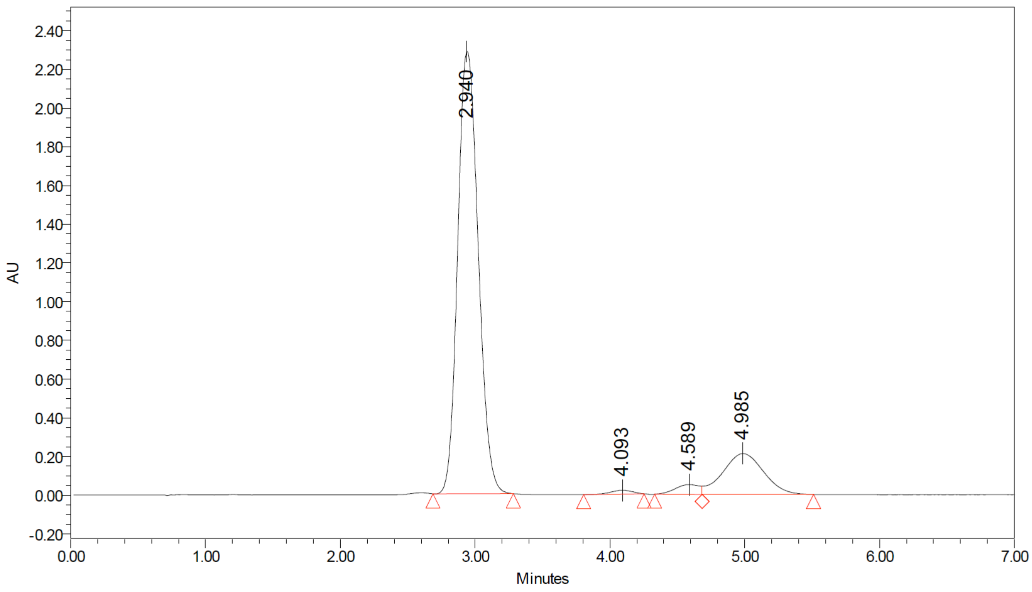

SFC chromatogram of compound (-)-**18a** ee 77%
Column: Chiralpak AD, 25cm x 4.6mm, 5µm
Eluent: CO_2:MeOH 70:30, 120 bar
Temperature: 40 °C
Flow rate: 4ml/min
UV detection at 220nm

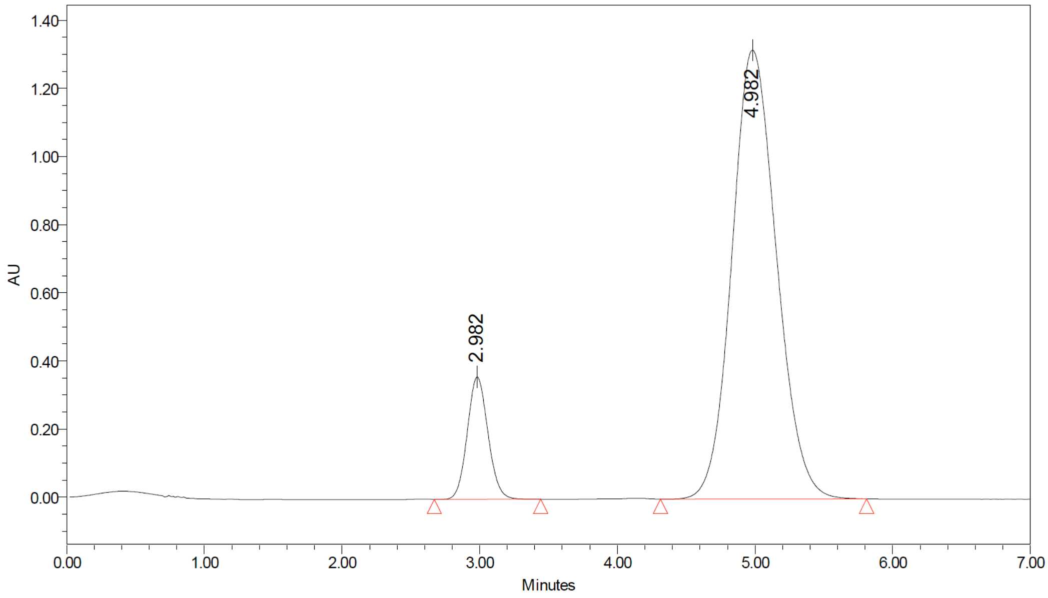

Chiral chromatogram of compound (±)-**18b**
Column: (S, S) Whelk-O1, 25cm x 4.6mm
Eluent: Hexane:DCM:IPA 56:40:4
Flow rate: 1ml/min
UV detection at 254nm

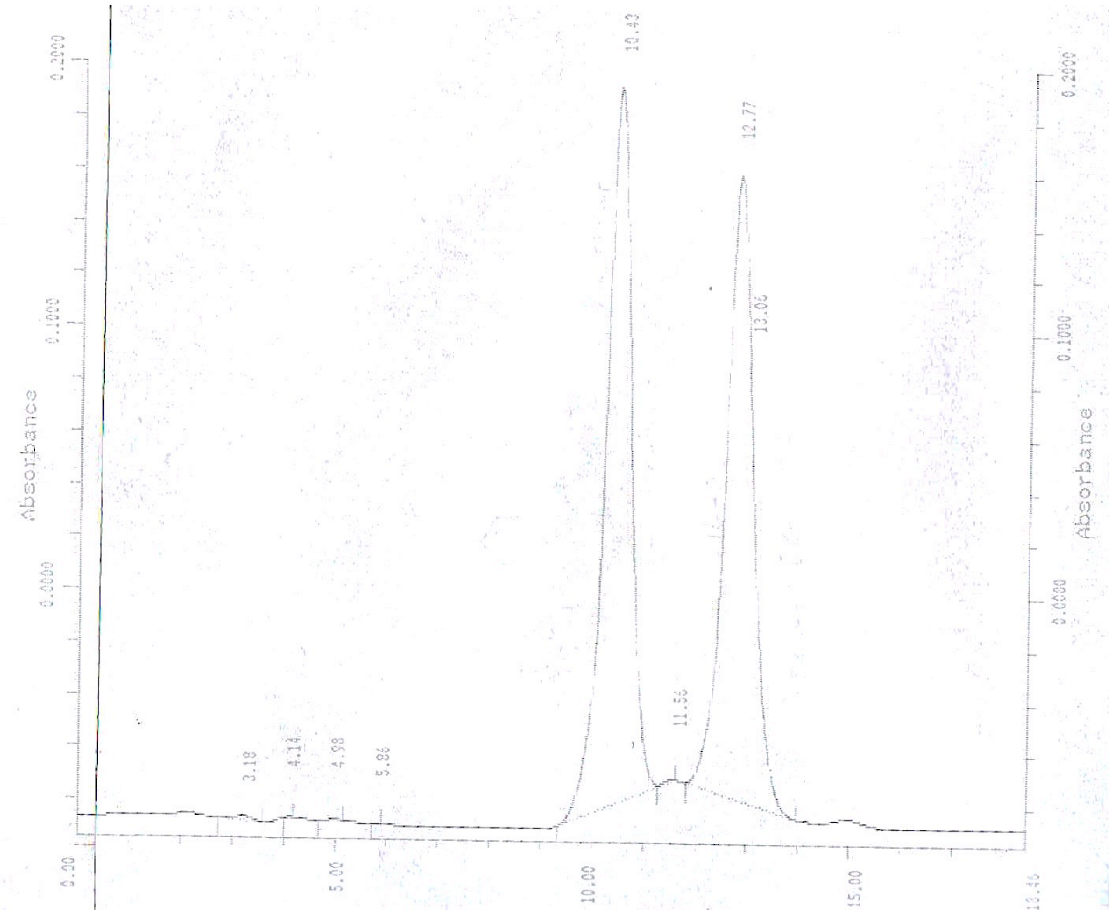

Chiral chromatogram of compound (-)-**18b** ee 88%
Column: (S, S) Whelk-O1, 25cm x 4.6mm
Eluent: Hexane:DCM:IPA 56:40:4
Flow rate: 1ml/min
UV detection at 254nm

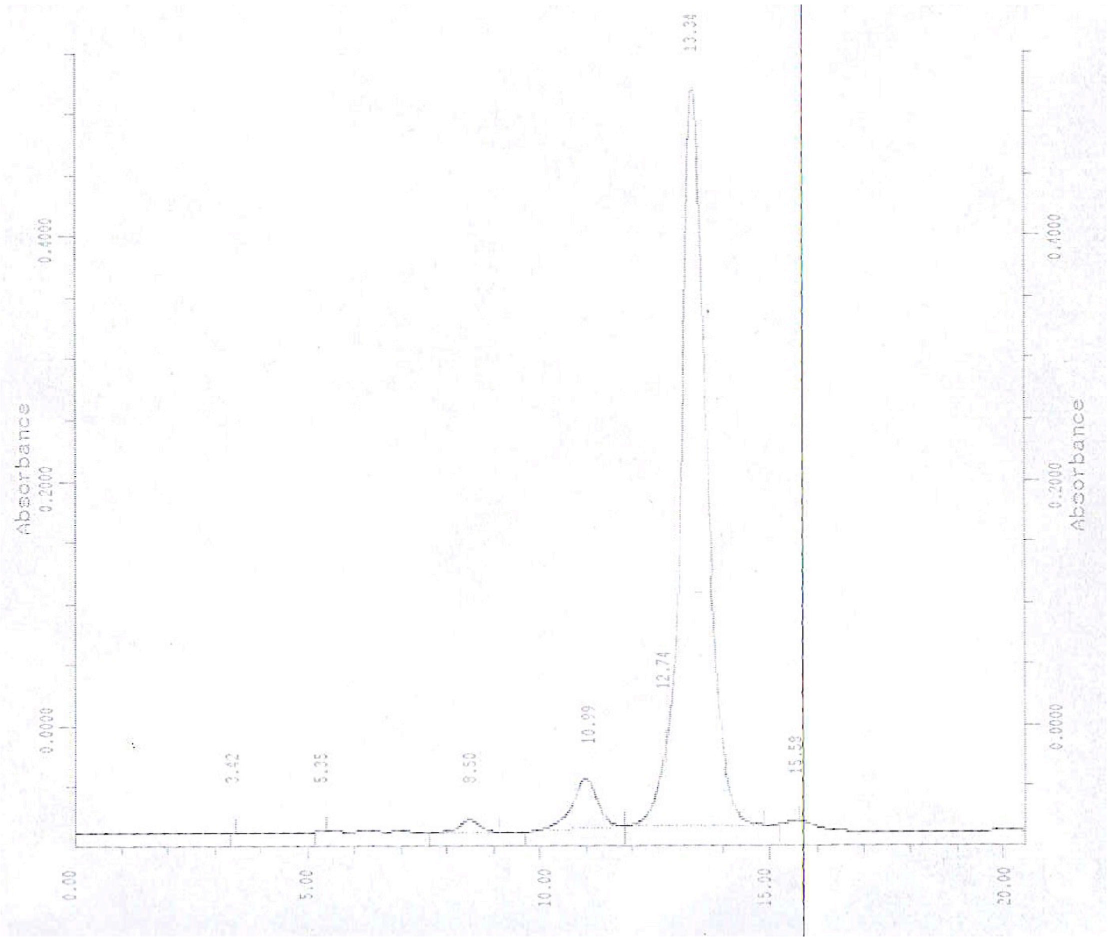

Chiral chromatogram of compound (+)-**18b** ee 82%
Column: (S, S) Whelk-O1, 25cm x 4.6mm
Eluent: Hexane:DCM:IPA 56:40:4
Flow rate: 1ml/min
UV detection at 254nm

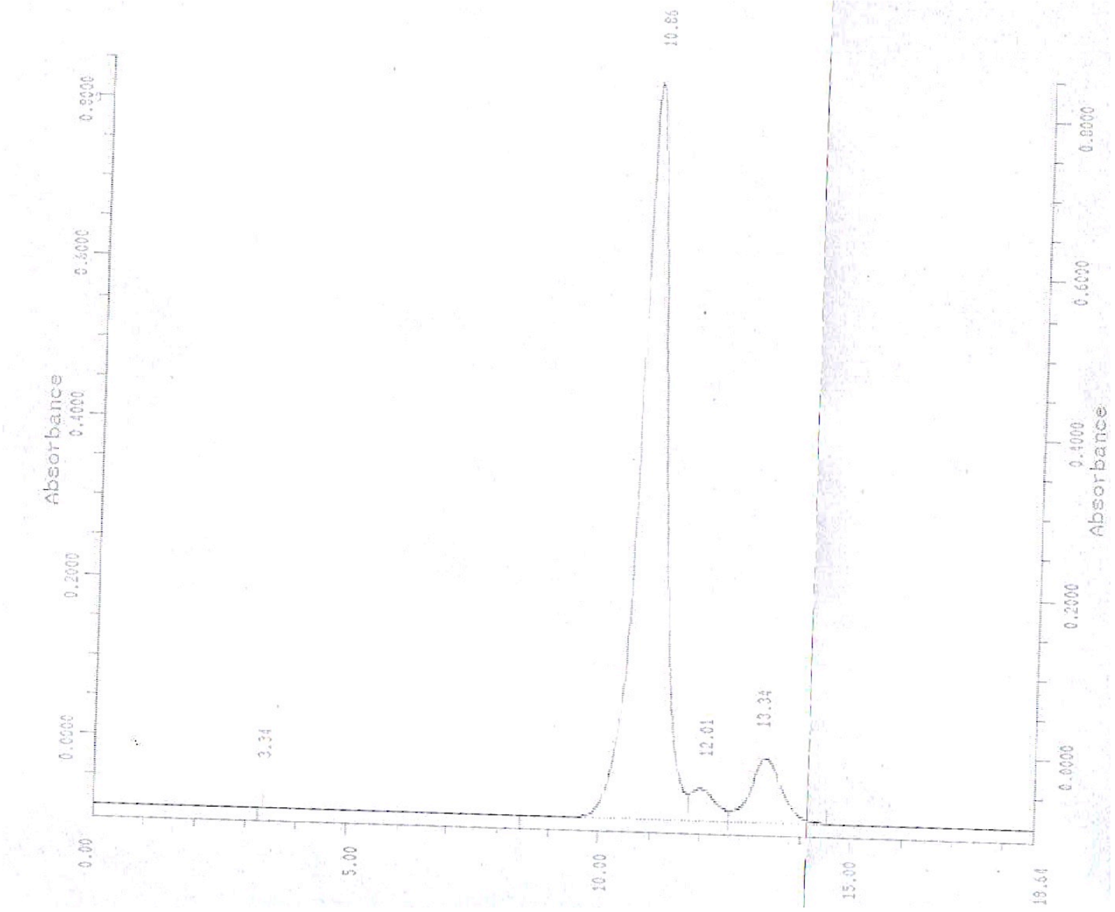

Chiral chromatogram of compound (-)-**10a** when using 1eq of Lawesson's reagent $[\alpha]_D$ (c=1.7, $CHCl_3$) -212, ee 97%
Column: (S, S) Whelk-O1, 25cm x 4.6mm
Eluent: Hexane:DCM:*t*BuOH 76:20:4
Flow rate: 1ml/min
UV detection at 254nm

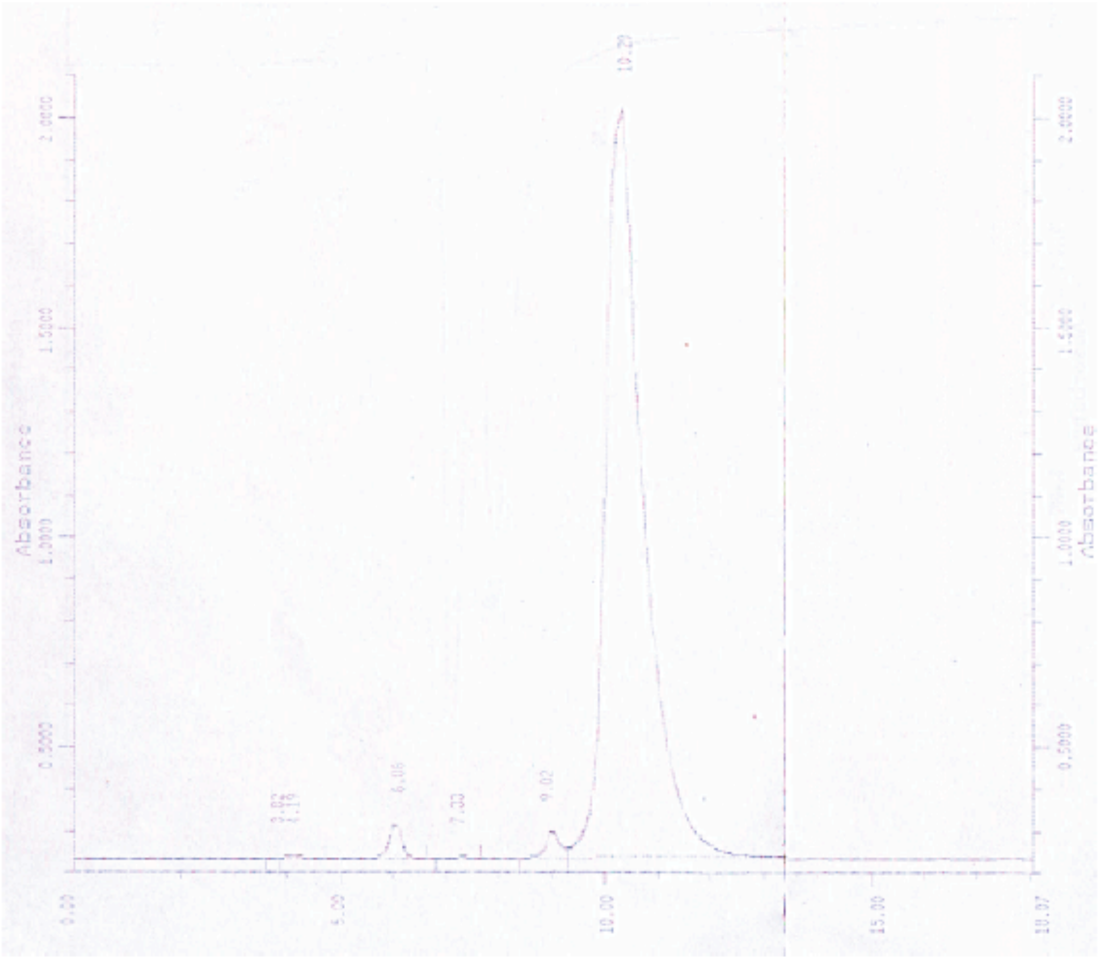

^{1}H- and ^{13}C-NMR of compound (-)-**10a** in $CDCl_3$
when using 1eq of Lawessons reagent

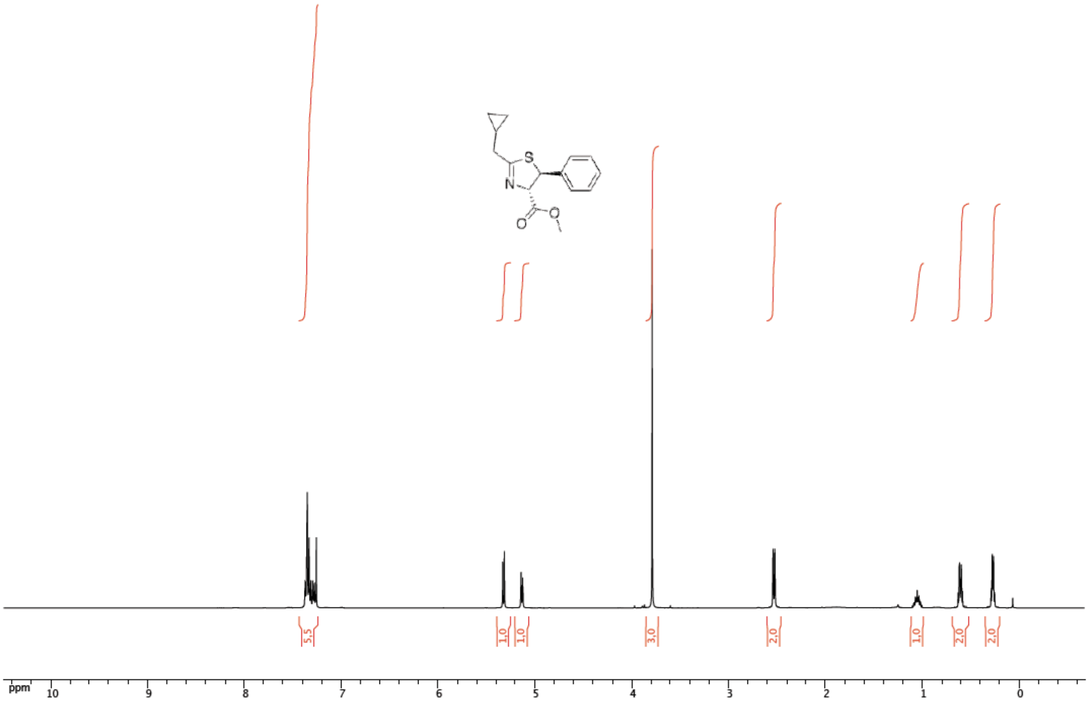

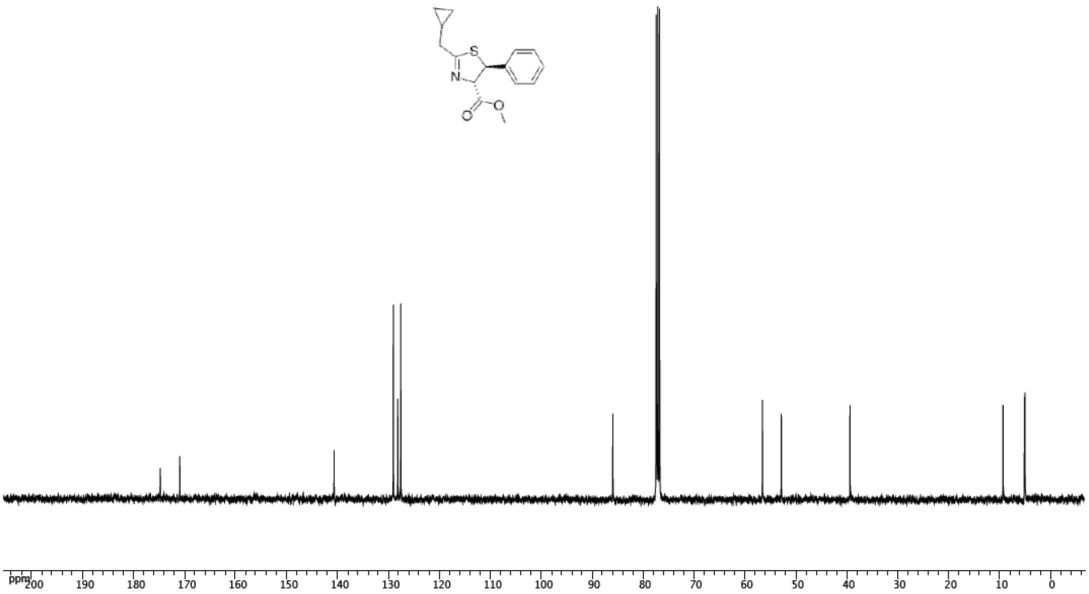

References

1) G. Xie, P. Chellan, J. Mao, K. Chibale, G. S. Smith *Adv. Synth. Catal*. **2010**, 352, 1641-1647

2) V. R. Chintareddy, A. Ellern, J. G. Verkade *J. Org. Chem.* **2010**, 75, 7166-7174

3) A. Fürstner, R. Martin, H. Krause, G. Seidel, R. Goddard, C. W. Lehmann *J. Am. Chem. Soc.* **2008**, 130, 8773-8787

4) A. El-Batta, C. Jiang, W. Zhao, R. Anness, A. L. Cooksy, M. Bergdahl *J. Org. Chem.* **2007**, 72, 5244-5259

5) A. Zhdanko, A. Schmauder, C. I. Ma, L. D. Sibley, D. Sept, F. Sasse M. E. Maier *Chem. Eur. J*. **2011**, 17, 13349-13357

6) S. Sato, M. Tetsuhashi, K. Sekine, H. Miyachi, M. Naito, Y. Hashimoto, H. Aoyama *Bioorg. Med. Chem.* **2008**, 16, 4685-4698

7) X. Lu, G. Yang *Org. Proc. Res. Dev.* **2000**, 4, 575-557

8) P. R. Fleming, K. B. Sharpless *J. Org. Chem.* **1991**, 56, 2869-2875

9) E. Chorell, P. Das, F. Almqvist *J. Org. Chem.* **2007**, 72, 4917-4924
